# LaF_3_ doped with Ce/Gd/Eu: energy transfer and excitation dependence of photoluminescence rise-and-decay kinetics

**DOI:** 10.3389/fchem.2025.1501039

**Published:** 2025-04-22

**Authors:** Andrii Shyichuk, Daria Szeremeta, Marcin Runowski, Eugeniusz Zych, Stefan Lis

**Affiliations:** ^1^ Faculty of Chemistry, University of Wroclaw, Wrocław, Poland; ^2^ Faculty of Chemistry, Adam Mickiewicz University in Poznań, Poznań, Poland; ^3^ Biophysics Department, Helmholtz-Zentrum Dresden-Rossendorf, Bautzner Landstraße Dresden, Germany

**Keywords:** multiexponential decay, energy transfer, photoluminescence rise, rise-and-decay kinetics, decay fitting lanthanides, luminescence, curve fitting, exponential decay

## Abstract

In this paper, we analyze time-domain luminescence measurements using multiexponential rise-and-decay functions. The relationships between these functions and the physics behind the analyzed photoemission kinetics are shown using several basic arbitrary photoluminescence systems. The advantages and disadvantages of the different types of functions mentioned are discussed. The paper is focused on peculiarities of the fitting process, such as the role of initial guess, under- and overfitting problems, and estimating fit quality (using patterns in the fit residual). Systems of differential equations are used to analyze selected cases by adjusting certain parameters. Hydrothermally treated LaF_3_:Ln^3+^ nanoparticles (where Ln^3+^ = Gd^3+^; Gd^3+^,Ce^3+^; Eu^3+^; Ce^3+^,Eu^3+^; Gd^3+^,Eu^3+^; or Ce^3+^,Gd^3+^,Eu^3+^) were used as a test case in which the role of interionic charge transfer was investigated by direct experimental measurements only, without the underlying theory. The methodological tips contained in this paper, although applied to the lanthanide (III) luminescence, should be interesting and useful for a much broader audience, for everyone working with smooth rise-and-decay curves.

## 1 Introduction

One of the key factors characterizing a photoluminescence system is the lifetime (*τ*) of a particular transition. Numerically, the lifetime is the time after which the intensity of emission reaches a fraction of 1/*e ≈* 0.3679 of its initial intensity, where *e* ≈ 2.718 is the base of the natural logarithm, also called Euler’s number. In the most basic case, without non-radiative relaxation, the lifetime is the reciprocal of the emission rate (transition rate), which is the fundamental property of the transition. The transition rate is, in turn, a function of the transition probability, determined by the quantum nature of the system, using Fermi’s golden rule (Dirac, 1927). In photoluminescence spectroscopy, lifetimes are used to find the quantum efficiency of a transition, as well as its population and relaxation pathways (de Sá et al., 2000).

Experimentally, the obtained plots of the temporal evolution of luminescence intensity are typically fitted using single- or multiexponential functions. The simplest exponential decay is described as *I*(*t*) = *A* exp (*−t*/*τ*), where *I* is the emission intensity at time *t*, *A* is the amplitude, t is time, and *τ* is the lifetime. Depending on the number of such components, terms such as “exponential decay,” “monoexponential decay,” “biexponential decay,” “multiexponential decay,” and “nonexponential decay”, are often used [e.g., Lakowicz (2006)]. The general rule is that the number of exponential components corresponds to the number of independent (distinct) photoluminescence species (centers) that participate in a given emission. The exponential decay function is an analytical solution to the differential rate equation describing the simplest photoluminescence system, composed of one excited state and one ground state. It is worth noting that in this model system, there may be several pathways of deactivating the excited state, but because all of them depend solely on the population of the excited state, the dynamics of such a state will be described by a monoexponential decay. The lifetime in such a case will be the reciprocal of the sum of the rates of all radiative (and non-radiative, if any) processes considered. In other words, regardless of the number of processes occurring in the excited state, as long as no other excited states are involved, a single-exponential decay and a single lifetime will be obtained in the photoluminescence measurement of the corresponding emission. This property is essentially the basis of the “one exponent–one site” principle.

The situation becomes more complex when more excited levels are involved. In a system where two excited states contribute to a single emission, two options are possible. In one scenario, the upper excited level may decay to a lower excited level from which emission is observed. A typical example is the Gd^3+^ ion, excited at 272 nm, emitting at 312 nm, and analyzed with a manifold-to-manifold degree of precision. In such a system, there are three levels, two of which (the excited states) are independent, while the population of the ground state depends on the populations of excited states. Alternatively, the emitting level can be populated from another photoluminescence center via energy transfer. In such a system, there are four states (two excited and two ground), of which (again) only two are independent. An example is a pair of Yb^3+^ ions, one of which is in its excited manifold. Both cases can be described by analytically solvable systems of rate equations. As a solution, the pulse kinetics is obtained: *I* = *A* (1 − exp (−*t*/*τ*_sens._)) exp (−*t/τ*_em._), where *τ*_sens_ is the sensitizer decay rate, and τ_em._ is the emitter decay rate. The mentioned rule of thumb still stands: two independent levels result in two exponents in the emission temporal evolution, with a clear correspondence: τ_sens._ is the reciprocal of the sensitization rate, while τ_em._ is the reciprocal of the emission rate.

In this paper, we emphasize the importance of high-quality numerical processing of photoluminescence data, paying attention to residuals and the number of exponents while tracking the physical meaning of the obtained results. The justification for the use of the pulse functions is presented. It will be shown that even simple systems of rate equations can be of great importance and are easy to construct and solve using available free software. Numerous issues regarding multiexponential rise-and-decay curve fitting are addressed, along with some practical advice.

We chose a well-known matrix material, namely, lanthanum fluoride, as a research case. LaF_3_ doped with other lanthanide Ln^3+^ cations as photoluminescence activators is one of the most studied Ln-based phosphor systems (Jacobsohn et al., 2010; Zhang and Huang, 2010). The famous and still-used Carnall, Carnall, and Crosswhite tables (Carnall and Crosswhite, 1978) refer to Ln^3+^ in LaF_3_. The materials are actually good phosphors, characterized by high chemical stability, insolubility, low phonon energy (meaning low non-radiative quenching), as well as simplicity of composition, crystal structure, and synthesis (Grzyb and Lis, 2009; Grzyb et al., 2016; Runowski and Lis, 2016). LaF_3_ is used in laser crystals (Li et al., 2019a; Li et al., 2019b; Hu et al., 2019a; Hizhnyakov and Orlovskii, 2017), glass-ceramic materials (Xia et al., 2019; Pawlik et al., 2019), composite materials (Secco et al., 2018; Ma et al., 2018), thin films (Gulina et al., 2017) nanoparticles and core-shell structures (Shao et al., 2017; Liu et al., 2015), and various kinds of sensors (Cho et al., 2019; Yu et al., 2018), including thermometric ones. In doped LaF_3_, regular (UV–Vis excited) luminescence (Grzyb and Lis, 2009; Shao et al., 2017; Poma et al., 2017), upconversion, or scintillation (Tang et al., 2018) can be obtained. The emission color can be tuned by the dopant composition (Poma et al., 2017). Quantum entanglement of Nd^3+^ ion pairs in the LaF_3_:1%Nd^3+^ crystal was discovered (Orlovskii et al., 2020). Recent studies regarding the materials include their preparation via wet chemistry processes such as co-precipitation and hydrothermal routes. There is a strong focus on biological and medical applications, such as *in vivo* bioimaging (Cheng et al., 2019), photodynamic therapy (Tang et al., 2018), etc.

The sensitizing Eu^3+^ emission with Gd^3+^ co-dopant is also typical in Ln^3+^-based phosphors. Gd^3+^ ions absorb light with wavelengths approximately 272 and 312 nm and can efficiently transfer the excitation energy to Eu^3+^ excited levels with appropriate energies. The excitation energy can jump from one Gd^3+^ to another Gd^3+^ several times before reaching the emission centers. This phenomenon is called energy migration and is another reason for using Gd^3+^ as a sensitizer (Zhang and Huang, 2010; van Schaik et al., 1993; van Schaik and Blasse, 1994; van Schaik et al., 1995). Absorption in the UV range of Ce^3+^ is even more efficient due to its parity-allowed f-d transitions. The excitation energy can be transferred from Ce^3+^ directly to Eu^3+^. Gd^3+^ may act as an intermediate sensitizer to avoid the undesired emission quenching caused by the possible intervalence charge transfer (Ce^3+^ + Eu^3+^ → Ce^4+^ + Eu^2+^) (Blasse, 1987; Li et al., 2017).

Energy transfer rates between ions depend on the distance between them and, in principle, can be calculated using complex approaches that require some experimental parametrization and/or *ab initio* calculations (Malta, 2008; Shyichuk et al., 2016a; Moura et al., 2016; Shyichuk et al., 2016b). One of the goals of the study was to test whether energy transfer rates could be estimated experimentally by comparison of the photoluminescence rise-and-decay lifetimes in Eu-doped, Ce-Eu-, Gd-Eu-, and Ce-Gd-Eu-codoped phosphors. The gathered experimental data were analyzed via numerical fitting of multiexponential functions to the experimental emission intensity over time rise-and-decay curves. As will be shown, it is of crucial importance to obtain fits that are unambiguous and give meaningful results (within a certain interpretation framework). During the analysis of data for the presented research, interesting information regarding curve fitting peculiarities was collected. Although this paper focuses on LaF_3_:Ce/Gd/Eu materials, the curve fitting conclusions obtained should be very helpful in any other field that involves processes with exponential rise and decay, the most topically related field being upconversion emission in systems involving energy transfer, such as phosphors co-doped with Yb^3+^/Er^3+^, Yb^3+^/Ho^3+^, and Yb^3+^/Tm^3+^.

## 2 Experimental procedures and methods

### 2.1 Synthesis and characterization

A hydrothermal route with parameters optimized for larger nanocrystals was selected. The hydrothermal route (compared to the co-precipitation technique) provides better crystallinity and higher luminescence quantum yield (Runowski and Lis, 2016; Vanetsev et al., 2017). Rare earth (RE) oxides: La_2_O_3_, Ce_2_O_3_, Eu_2_O_3_, and Gd_2_O_3_ (99.99%, Stanford Materials, United States) were dissolved separately via dropwise addition of HNO_3_ to the oxide powder. The resulting solutions were dried at 60°C, transferred to volumetric flasks, and solved in distilled water.

Ammonium fluoride, NH_4_F (98+%, Sigma-Aldrich, Poland), was used as a source of fluoride ions. Citric acid trisodium salt dihydrate (Sigma-Aldrich, 97%, Poland) was used as received without further purification. Deionized water was used for the synthesis.

Appropriately calculated (for 0.5 g of the product) amounts of aqueous solutions of metal nitrate were mixed in a beaker. Water was added to give a total volume of 50 mL (Solution 1). In another beaker, 150% of the stoichiometric amount of NH_4_F was dissolved by stirring in 50 mL of water at 60°C–70°C (Solution 2). After the complete dissolution of NH_4_F, Solution 1 was added dropwise to Solution 2, under continuous stirring, without temperature control. After the addition, the mixture was left under stirring for another 15 min. The mixture was then allowed to precipitate, and the clear portion of the solution was decanted. The precipitate was purified via sedimentation in a centrifuge, decantation, and washing with distilled water: sedimentation for 3 min at 3,000 rpm, decanting, adding 35–40 mL of water, sedimentation for 6–7 min at 4,000–4500 RPM, decanting, addition of 35–40 mL water, and sedimentation for 12 min at 5,500 rpm. The purified precipitate was redispersed in approximately 50 mL of water and subjected to hydrothermal treatment in a Teflon vessel (20 h, 200°C). Then, the product was sedimented, the water was decanted, and the sediment was dried in an oven at 60°C–70°C.

X-ray diffraction (XRD) patterns were recorded using a Bruker AXS D8 Advance diffractometer in the Bragg–Brentano setup, with Cu K_α1_ radiation (λ = 1.5406 Å), in the 6°–60° of 2Θ. Transmission electron microscopy (TEM) images were recorded using an FEI Tecnai G2 20 X- TWIN transmission electron microscope, which used an accelerating voltage of 200 kV. The photoluminescence decay curves and excitation spectra were measured under pulsed laser excitation (Opolette 355LD UVDM tunable pulsed laser (Opotek Inc.) operating at 20 pulses per second) QuantaMaster™ 40 spectrophotometer (Photon Technology International) with an R928 photomultiplier as a detector (from Hamamatsu). All spectra were measured at room temperature (293 K) and were appropriately corrected for the instrumental response. The excitation laser was characterized by a pulse length of 7–10 ns.

### 2.2 Numerical methods: curve fitting

All of the fitting operations were performed using the custom code we developed. The code was written in the Python programming language, and the SciPy module (version 0.19.0) was used for the actual fitting routine. The code is available in its current form upon request. The code was used to read the input files, normalize the curves, downsample the data if necessary, and plot the fitted functions and the fit residual. Most importantly, the code provides a command-line-based user interface, where a guess for the fit can be typed in manually, copied and pasted, or called from the history of previous fits. In a typical procedure, the user loads a file and specifies the guess for the fit in the form of function name, list of parameters, another function name, list of parameters, and so on. The different functions (if there is more than one) are summed. It is possible to specify an ambiguously long set of any of the functions known to the code and to easily create new functions.

In the interface, fit results appear in the history together with guesses, meaning that the result of any of the fits can easily be used as a guess for a new fit; below, we shall call such an operation re-fitting. Re-fitting was used to test the results for stability. If a given set of fitted parameters is indeed optimal, then using it as a guess must, first, result in nearly identical parameters after fitting, and second, the fitting must converge after a small number of function evaluations (typically three to seven). Least squares fitting is a variational method of minimizing the difference between the experimental data and the fitted curve (i.e., minimizing the residual) and involves searching for a local minimum in an abstract space of fitting parameters. The fact that the result does not change significantly between cycles means that a given set of parameters corresponds to a certain stable minimum and is considered optimal. A small number of function evaluations means that the minimum is “deep” enough and is indeed a minimum. Another way to look at it is that a “correct” result must be repeatable; otherwise, it is not “correct.” The quotation marks are used because no fitted result is exact: all experimental data contains noise and uncertainties that can affect the fits.

Of the available least squares methods, the setup described below proved to be optimal. Other methods seemed more sensitive to the initial guess and more prone to sticking to local minima corresponding to obvious bad fits. In particular, least squares (rather than some more advanced minimization/optimization approach) was selected for historical and reverse-compatibility purposes: it is something people have used, are still using, and will likely use for the foreseeable future. Furthermore, least squares is the most basic approach and is readily available in commonly used software such as Origin, Matlab, Gnuplot, and Python. Finally, for the problem at hand, least squares gets the job done quite quickly.

Prior to the fitting, the decay curves were normalized by dividing by the peak value of the raw curve. The fitting was performed using the *least_squares* module of SciPy, with the Trust Region Reflective (“trf”) method (Branch et al., 1999; Byrd et al., 1988), 3-point Jacobian, *lsmr* solver (Fong and Saunders, 2011), and *x_scale* option. A description of the module can be found in SciPy (2011). In the *least_squares* module, the input is a vector of guess values of the fitting parameters; for example, *x* = [ *t*_*0*_, *I*_*0*_, *A*_*0*_, *τ*_*1*_, *τ*_*2*_]. The module calculates the gradient in each parameter, changing it by 1 × 10^−8^ and recalculating the residual function. Because the absolute initial values of the variables may differ by several orders of magnitude (e.g., *τ*_*rise*_ = 10 and *A*_3_ = 0.0002), they are changed in relatively different steps. However, if we define the *x_scale* vector equal to *x*, the problem is redefined in *xs = x/x_scale*. The guess values become coefficients, and the code manipulates the initial vector of ones, (Dirac, 1927), changing them (initially) by 1 × 10^−8^. Therefore, each parameter is changed proportionally to its value and has a similar effect on the cost function (Moré, 1978). The use of *x_scale* resulted in much greater stability of the fits, that is, faster convergence, faster change toward a “correct” fit, fewer incorrect fits, and smaller chance of getting “strange” (way too small or way too big) values of the fitted parameters.

Most often, the sum of the squared residuals (the residual being the fitted curve minus the experimental curve) was used as the loss function. However, in a few problematic cases, the Cauchy loss function was used.

Because there were 50,000 data points on each curve, and the functions included many exponential components, it would take a long time to fit completely from scratch. That is why downsampling was used: for each group of subsequent *n* points, the average values of both time and intensity were calculated, resulting in one data point instead of the original *n*. Noise was also effectively reduced in this procedure. The fits were performed at *n* = 30 (the original number of points was reduced by a factor of 30), then refits were performed at *n* = 20, 10, 5, and finally at *n* = 1, that is, without downsampling. In other words, the hardest part of trial-and-error search for optimal function was performed using smaller data sets, and the resulting fit was then corrected by re-fitting with a gradually increasing number of points.

One of the crucial indicators of fit consistency is the residual plot. Ideally, such a plot cannot contain any signal; only noise must remain (that is, points randomly oscillating up and down from zero intensity). Such residuals are referred to as “flat” because they are essentially (noisy) horizontal lines, with *I* = 0. In the case of incomplete fits, the residual plot contains some signal in addition to the noise; some pattern (e.g., rise, decay, rise, and decay, waves) is obvious (Lakowicz, 2006). Often, the pattern can be described as sine-like waves (variable-amplitude chirps, also called chirplets): a noisy residual curve going up and down with a gradually changing period.

Summarizing, for each curve considered, the fitting procedure and search for the optimal function was continued until a flat (signal-free, noise-only) residual was obtained. The fitting started with downsampled curves; once a suitable solution was found, refits were performed with gradually smaller downsampling and, finally, for the entire dataset. At each step, subsequent refits were performed several times until it was clear that the solution was stable; that is, the result very similar to the guess is obtained after a small number of function evaluations, namely, three to seven.

### 2.3 The model systems

The functions used to fit the curve were linear combinations of the pulse functions of the kind *I* = *A* (1 − exp (−*t*/*τ*_1_)) exp (−*t*/*τ*_2_). In order to justify the use of such functions, several simple model systems were proposed and described using differential equations. The sets were solved analytically, and the solutions were pulse functions. We have obtained the solutions using the WolframAlpha mathematical engine (Wolfram Alpha LLC, 2018).

#### 2.3.1 One-level luminescence after pulse excitation

**FIGURE 1:**
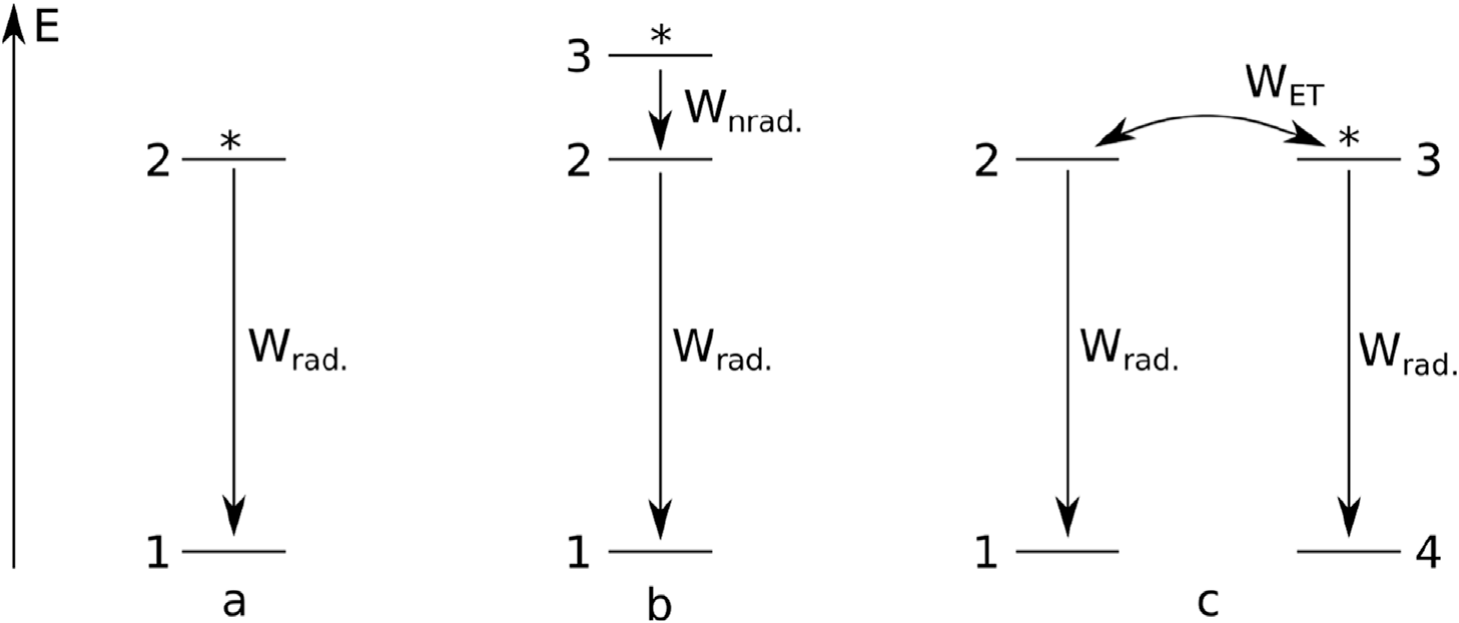
Simple models of photoluminescence systems with: a single emitting level **(a)**, an emitting level and a sensitization level **(b)**, an energy transfer **(c)**. Asterisks indicate initially excited levels.

In the simplest case, luminescence is a spontaneous process characterized by a first-order differential equation kinetics. In such a process, the initial population of some species (atoms in excited states) is reduced at a constant ratio independent of the population. It is convenient to represent the set of (atoms in their) excited states by a single level, while another level represents the set of (atoms in their) ground states. Such a simple system is visualized in Figure 1a. It is convenient to normalize the total population of the levels to 1. Then, it is clear that the population of the ground state *n*_1_ is a function of the population of the excited state *n*_2_, namely, *n*_1_ = 1 − *n*_2_. There is thus only one independent level in the system; only one rate equation is needed, and the system is named “one-level.” Without excitation, *n*_1_ = 1, and *n*_2_ = 0. If we consider such a system in a condition after a short pulsed excitation, *n*_2_ would be non-zero. The corresponding luminescence intensity (which is proportional to the population of the excited level) temporal evolution could be described by the following equations:

$$dn_{2}/dt=–\;n_{2}\left(t\right)\;W_{\text{rad}}.$$ (1)

$$I=I_{0}\;\exp\left(\;–t\;W_{\text{rad}.}\right)=I_{0}\;\exp\left(\;–t/\tau_{\text{rad}.}\right).$$ (2)

Above, *n*_2_ is the emitting level population, *W*_rad._ is the radiative rate of the emission, *t* is time, and *τ* = 1/*W*_rad._ is the photoluminescence lifetime. Equation 2 is the integrated form of Equation 1, and can be derived analytically. For simplicity, we assume a purely radiative process in Equation 1.

We would like to point out that Equations 1, 2 clearly indicate the “one exponent–one site” principle. In other words, two-exponent decays absolutely cannot be interpreted as resulting from two processes occurring at the same level (provided that the participation of other levels is definitely excluded). Let us assume that level 2 also has a non-radiative decay associated with it, with a *W*_nrad._ rate. We would need to extend the right-hand side of Equation 1 with another term, − *n*_2_(*t*) *W*_nrad._. It is clear that−*n*_2_(*t*) *W*_rad._ − *n*_2_(*t*) *W*_nrad._ = − *n*_2_(*t*) (*W*_rad._ + *W*_nrad._) (Lakowicz, 2006), and we are back to Equation 1. This modified two-process one-level system will exhibit monoexponential decay, although this time with a lifetime *τ* = 1/(*W*_rad._ + *W*_nrad._). The same would be true for an arbitrary number of processes and even for different final states (Chambers et al., 2009). For instance, the 4f-4f emission of the Ln^3+^ ion in a material with only one kind of coordination geometry [e.g., LaF_3_ (Li et al., 2019a; Li et al., 2019b; Orlovskii et al., 2020; Li et al., 2020; Hu et al., 2019b; Hong et al., 2017; Liu et al., 2014; Hong et al., 2015), GdAl_3_(BO_3_)_4_ (GAB) (Liao et al., 2004; Liao et al., 2005)] and low dopant concentrations must exhibit single-exponential decays unless cross-relaxation processes occur [including dopant ion clustering (Kurz and Wright, 1977)].

#### 2.3.2 Two-level luminescence with non-radiative relaxation

Equation 2 assumes a constant initial population of the emitting level, which is feasible in the case when atoms are excited with a short pulse, and the excitation level is also the emission level. In the case when the emitting level is populated by some process from a different level, the *I*_0_ in Equation 2 is no longer constant and changes according to the population rate. It is acceptable to simply replace *I*_0_ with an exponential rise of the (1 − exp (−*t W*_rise_)) kind and obtain a two-exponential rise-and-decay kinetics. Yet, we present analytical solutions of the respective system of rate equations.

In the simplest two-level example, atoms can be excited to a level above the emitting level. Here, again, the population *n*_1_ of the ground state is the dependent parameter, *n*_1_ = 1 − *n*_2_ − *n*_3_. The case is illustrated in Figure 1b, and Equations 3, 4 are as follows:

$$dn_{2}/dt=–\;n_{2}\left(t\right)\;W_{\text{rad}.}+n_{3}\left(t\right)\;W_{\text{nrad}.}$$ (3)

$$dn_{3}/dt=–\;n_{3}\left(t\right)\;W_{\text{nrad}.}$$ (4)

Such a set is also solvable analytically. Level 3 would experience a single-exponential decay, while the solution for level 2 reads:

$$n_{2}\left(t\right)=n_{3‐0}\;W_{\text{nrad}.}\;\left(\exp\left(–t\;W_{\text{nrad}.}\right)\;–\;\exp\left(\;–t\;W_{\text{rad}.}\right)\right)\;/\;\left(\;W_{\text{rad}.}\;–\;W_{\text{nrad}.}\right).$$ (5)

$$n_{2}\left(t\right)=A\;\left(\;1\;–\exp\left(–t\;W_{\text{nrad}.}\right)/\;\exp\left(\;–t\;W_{\text{rad}.}\right)\;\right)\;\exp\left(\;–t\;W_{\text{rad}.}\right)=A\;\left(\;1\;–\exp\left(–t\;\left(W_{\text{nrad}.}\;–\;W_{\text{rad}.}\right)\right)\right)\;\exp\left(\;–t\;W_{\text{rad}.}\right).$$ (6)

Where *A* = − *n*_3-0_ *W*_nrad._/(*W*_rad._ − *W*_nrad._), and *n*_3-0_ is the initial population of level 3, *n*_3_ at *t* = 0. Noteworthy, the rates here are part of the amplitudes. In Equation 6, the radiative decay part, exp (−*t W*_rad._), has been moved from the brackets. Equation 5 was, therefore, transformed into Equation 6 to show the pulse function. Another important notion is that the pulse function lifetimes do not have a one-to-one correspondence with the decay rates—namely, the rise lifetime is a reciprocal of *W*_nrad._—*W*_rad._.

#### 2.3.3 Two-level luminescence with energy transfer

In yet another case (Figure 1c), atoms can become involved in energy transfer processes, during which excited atoms undergo a transition to their ground state and transfer the energy to other atoms (originally in their ground states), promoting them to their excited states. At the same time, all atoms may undergo radiative decay. This system is again characterized by two independent levels (*n*_1_ = 1 − *n*_2_, *n*_4_ = 1 − *n*_3_), which leads to a more complex set of rate equations (Malta, 2008; Shyichuk et al., 2016b):

$$dn_{2}/dt=–\;n_{2}\left(t\right)\;W_{\text{rad}.}+n_{3}\left(t\right)\;\left(1\;–\;n_{2}\left(t\right)\right)\;W_{\text{ET}}\;–\;n_{2}\left(t\right)\;\left(1\;–\;n_{3}\left(t\right)\right)\;W_{\text{ET}.}$$ (7)

$$dn_{3}/dt=–\;n_{3}\;W_{\text{rad}.}\;–\;n_{3}\left(t\right)\left(1\;–\;n_{2}\left(t\right)\right)\;W_{\text{ET}}+n_{2}\left(t\right)\;\left(1\;–\;n_{3}\left(t\right)\right)\;W_{\text{ET}}.$$ (8)

Some assumptions of this model require clarification. Both initial values *n*_1_ and *n*_3_ (*n*_1-0_ and *n*_3-0_) are equal to unity (*n*_1-0_ = *n*_3-0_ = 1), while *W*_rad._ is the same for levels 2 and 3. Such conditions do not affect the overall conclusion, and the solutions are greatly simplified by them. Another important assumption is the **bidirectionality of energy transfer**. Because *n*_2-0_ is zero and *n*_3-0_ is non-zero, the energy will predominantly flow toward level 2. However, because the levels here are in perfect resonance and the transition multipoles are the same, it is much more physically correct to make a transfer bidirectional. (For instance, let us consider the dipole–dipole mechanism and let the transition dipoles be *D*_12_ and *D*_34_. The direct ET rate is proportional to *D*_donor_ · *D*_acceptor_ = *D*_34_ · *D*_12_, while the reverse ET rate is proportional to *D*_donor_ · *D*_acceptor_ = *D*_12_ · *D*_34_). Interestingly, bidirectional transfer also simplifies both the set and its solution because the non-linear parts cancel out. It is also worth noting that the radiative transfer or reabsorption (2-1 emission followed by 4-3 absorption) process can be viewed as an alternative to non-radiative energy transfer. It would be proportional to n_2_, n_3_, and the transition dipoles—that is, the rate equation term would be proportional to n_2_ · n_3_ · D_12_ · D_34_—which is exactly the proportion for the dipole-dipole energy transfer mechanism. Opening the brackets in Equations 7, 8 gives Equations 9, 10:

$$\begin{array}{ll} dn_{2}/dt & =–\;n_{2}\left(t\right)\;W_{\text{rad}.}+n_{3}\left(t\right)\;W_{\text{ET}}\;–\;n_{2}\left(t\right)\;n_{3}\left(t\right)\;W_{\text{ET}}\;–\;n_{2}\left(t\right)\;W_{\text{ET}}\\ & +n_{2}\left(t\right)\;n_{3}\left(t\right)\;W_{\text{ET}}\\ & =–\;n_{2}\left(t\right)\;W_{\text{rad}.}+n_{3}\left(t\right)\;W_{\text{ET}}\;–\;n_{2}\left(t\right)\;W_{\text{ET}}\\ & =n_{2}\left(t\right)\;\left(\;–W_{\text{rad}.}\;–\;W_{\text{ET}}\;\right)+n_{3}\left(t\right)\;W_{\text{ET}.} \end{array}$$ (9)

$$\begin{array}{ll} dn_{3}/dt & =–\;n_{3}\;W_{\text{rad}.}\;–\;n_{3}\left(t\right)\;W_{\text{ET}}+n_{2}\left(t\right)\;n_{3}\left(t\right)\;W_{\text{ET}}\\ & +n_{2}\left(t\right)\;W_{\text{ET}}\;–\;n_{2}\left(t\right)\;n_{3}\left(t\right)\;W_{\text{ET}}\\ & =–\;n_{3}\;W_{\text{rad}.}\;–\;n_{3}\left(t\right)\;W_{\text{ET}}+n_{2}\left(t\right)\;W_{\text{ET}}\\ & =n_{2}\left(t\right)\;W_{\text{ET}}+n_{3}\left(t\right)\;\left(\;–W_{\text{rad}.}\;–\;W_{\text{ET}}\;\right). \end{array}$$ (10)

Setting *n*_2-0_ = 0 and *n*_3-0_ = *A* gives a solution:

$$n_{2}\left(t\right)=½\;A\exp\left(–t\;W_{\text{rad}.}\right)\;\left(\;1\;–\exp\left(–2\;t\;W_{\text{ET}}\;\right)\right)$$ (11)

$$n_{3}\left(t\right)=½\;A\exp\left(–t\;W_{\text{rad}.}\right)\;\left(\;1+\exp\left(–2\;t\;W_{\text{ET}}\;\right)\right)$$ (12)

Equation 11 is clearly a pulse function, while the opening brackets Equation 12 would give a two-exponential decay. It is important to emphasize that the biexponential character on *n*_3_(*t*) is not related to the two processes occurring at the level (radiative decay and energy transfer). This indicates that this level interacts strongly with another independent level. By construction, there are technically three processes that involve the *n*_3_(*t*) population: the decay, the energy transfer, and the back transfer.

Sometimes (Chambers et al., 2009; Li et al., 2010), a slightly different form of the pulse function is used, with an additional parameter corresponding to the initial population of the emitting level. If a section of the fitted experimental curve does not perfectly correspond to the time immediately before the excitation pulse (i.e., some part of the rise is lost), the population of the emitting level may actually be non-zero. However, the temporal offset will compensate for this when fitting the pulse function (Equation 11). A more detailed discussion is provided in Supplementary Data Sheet S2, Sections SI.1, SI.6.

#### 2.3.4 Intermediate summary

Opening the brackets in the pulse function and using the e^*x*^ e^*y*^ = e^*x*+*y*^ identity gives:

$$\begin{array}{ll} I & =A\exp\left(–t\;W_{\text{decay}}\right)\;\left(\;1\;–\exp\left(–t\;W_{\text{rise}}\;\right)\right)\\ & =A\exp\left(–\;t\;/\;W_{\text{decay}}\right)\;–\;A\exp\left(–\;t\;/\;W_{\text{decay}}\right)\exp\left(–t\;W_{\text{rise}}\;\right)\\ & =A\exp\left(–\;t\;/\;W_{\text{decay}}\right)\;–\;A\exp\left(–t\;\left(W_{\text{decay}}+W_{\text{rise}}\;\right)\right). \end{array}$$ (13)

In other words, the pulse function (and also any linear combination of pulse functions) is the sum of exponential functions. “Decay exponents” have positive coefficients/amplitudes, while “rise exponents” have negative amplitudes (Equation 13). This property can be useful in difficult curve fitting cases, where it is hard to find a matching function. Consecutive fits with increasing numbers of exponential components can, at some point, result in a good fit. This way, the required number of exponential components (i.e., the number of independent levels) is found, and the lifetimes are approximated.

One problem with this approach is its susceptibility to overfitting. The exponential decay can be perfectly fitted to one, two, three, or more exponents without clearly identifying overfitting (see Supplementary Data Sheet S2, Section SI.2). In a zero-noise virtual case, such a fit would result in identical lifetimes, but even small amounts of noise change this. Pulse functions are less prone to overfitting than the multiexponential decay: if the noise contains no signal, the fitting is complete, period.

Another problem is the obvious intermixture of the rates (Equation 13). In a pulse function, as in Equation 11, there is a clear physical sense of its rise-and-decay lifetimes and the corresponding rates. In a plane sum of exponential components, the origin of the lifetimes is not clear. It is worth noting that we created such an intermixture artificially in Equation 6 to enforce the pulse function as a solution. However, we believe that energy transfer processes are much more important and defining. Consequently, recognizing the pulse function as a solution to the two kinds of sensitization (relaxation and energy transfer) provides a framework for further fitting and interpretation.

As the number of independent levels and processes in the system grows, solutions become more complex and impractical. From a practical point of view, linear combinations of pulse functions were found to be the most feasible. Therefore, we did not analyze more complex model systems. The above examples provide a solid justification for the use of pulse functions.

### 2.4 The functions

In the examples, we considered processes with monoexponential rise and monoexponential decay. There may be more complicated cases where there are several emitting sites characterized by different decay rates. Considering the same rise lifetimes, the total observed emission intensity can be described by Equation 14, which is basically an exponential rise multiplied by the sum of two exponential decays (a biexponential decay):

$$I=A_{0}\;\left(1\;–\;\exp\left(\;–\;t\;/\;\tau_{\text{rise}}\;\right)\right)\;\left(\exp\left(–\;t\;/\;\tau_{1\;\text{decay}}\right)+A_{2}\exp\left(–\;t\;/\;\tau_{2\;\text{decay}}\right)\right).$$ (14)

Note the missing *A*_1_ coefficient before the *τ*_1 decay_ exponent (which is actually held constant and equal to unity). It can be shown that *A*_1_ is absorbed by the *A*_0_ coefficient and is thus redundant from the fitting point of view; freezing *A*_2_ = 1 results in essentially the same fit result with one less degree of freedom, thus improving the fit stability.

Similarly, we can define a two-rise-one-decay kinetics, which can be interpreted as two sites that differ in population rates but have the same decay rates:

$$I=A_{0}\;\left(1\;–\;\exp\left(\;–\;t\;/\;\tau_{1\;\text{rise}}\right)+A_{2}\;\left(1\;–\;\exp\left(–\;t\;/\;\tau_{2\;\text{rise}}\;\right)\right)\;\right)\exp\left(–\;t\;/\;\tau_{\text{decay}}\right).$$ (15)

Again, one of the coefficients is redundant.

Functions like Equations 14, 15 have been successfully used, for example, by Bartkowiak et al. (2019) and Runowski et al. (2018).

Things become more complicated if we consider two (or more) rise components and two (or more) decay components. Consider Equation 16 and compare it with Equations 17, 18. Although Equation 16 is a multi-rise-multi-decay function, the other two are sums of one-rise-one-decay functions.

$$I=A_{0}\;\left(1\;–\;\exp\left(\;–\;t/\tau_{1\;\text{rise}}\right)+A_{2}\;\left(1\;–\exp\left(–\;t/\tau_{2\text{rise}}\;\right)\right)\;\right)\;\left(\exp\left(–\;t/\tau_{3\;\text{decay}}\right)+A_{4}\exp\left(–\;t/\tau_{4\;\text{decay}}\right)\right).$$ (16)

$$I=A_{0}\;\left(1\;–\;\exp\left(\;–\;t/\tau_{1\;\text{rise}}\;\right)\right)\exp\left(–\;t/\tau_{3\;\text{decay}}\right)+A_{1}\;\left(1\;–\;\exp\left(\;–\;t/\tau_{2\;\text{rise}}\;\right)\right)\exp\left(–\;t/\tau_{4\;\text{decay}}\right).$$ (17)

$$I=A_{0}\;\left(1\;–\;\exp\left(\;–\;t/\tau_{1\;\text{rise}}\;\right)\right)\exp\left(–\;t/\tau_{4\;\text{decay}}\right)+A_{1}\;\left(1\;–\;\exp\left(\;–\;t/\tau_{2\;\text{rise}}\;\right)\right)\exp\left(–\;t/\tau_{3\;\text{decay}}\right).$$ (18)

While Equations 17, 18 look essentially similar, they represent two ambiguous solutions. Fitting the same curve using Equations 16, 17 or Equation 18 gives approximately the same *τ*_1–4_, *R* ^2^, and residual pattern. However, there are two different ways to distribute the rise-and-decay lifetimes between the two one-rise-one-decay functions, namely, (rise-1 times decay-3; rise-2 times decay-4) or (rise-1 times decay-4; rise-2 times decay-3). They may correspond to a site with *τ*_1_ rise lifetime, *τ*_3_ decay lifetime, and another site with *τ*_*2*_ rise lifetime, *τ*_4_ decay lifetime. Alternatively, there might be a site with *τ*_1_ rise lifetime, *τ*_4_ decay lifetime, and the other with *τ*_2_ rise lifetime, *τ*_3_ decay lifetime. Equation 16 is apparently less ambiguous, stating that there are two rise and two decay components in the fitted kinetics.

Opening the brackets shows clearly that Equation 16 is the sum of four pulse functions, where the interdependence of the parameters is obvious: all of the amplitudes affect more than one of the exponent components (Equation 19). Consequently, none of the components can reach 0 and not affect the others at the same time. Such functions, although they result in stable fits, can be very difficult to interpret. Functions of this kind will be referred to as convoluted below.

$$\begin{array}{ll} I= & A_{0}\;\left(1\;–\;\exp\left(\;–\;t/\tau_{1\;\text{rise}}\;\right)\right)\exp\left(–\;t/\tau_{3\;\text{decay}}\right)\\ & +A_{0}\;A_{4}\;\left(1\;–\;\exp\left(\;–\;t/\tau_{1\;\text{rise}}\;\right)\right)\exp\left(–\;t/\tau_{4\;\text{decay}}\right)\\ & +A_{0}\;A_{2}\;\left(1\;–\;\exp\left(\;–\;t/\tau_{2\;\text{rise}}\;\right)\right)\exp\left(–\;t/\tau_{3\;\text{decay}}\right)\\ & +A_{0}\;A_{2}\;A_{4}\;\left(1\;–\;\exp\left(\;–\;t/\tau_{2\;\text{rise}}\;\right)\right)\exp\left(–\;t/\tau_{4\;\text{decay}}\right). \end{array}$$ (19)

Alternatively, consider functions of the following kind:

$$\begin{array}{ll} I= & A_{13}\;\left(1\;–\;\exp\left(\;–\;t/\tau_{1\;\text{rise}}\;\right)\right)\exp\left(–\;t/\tau_{3\;\text{decay}}\right)\\ & +A_{14}\;\left(1\;–\;\exp\left(\;–\;t/\tau_{1\;\text{rise}}\;\right)\right)\exp\left(–\;t/\tau_{4\;\text{decay}}\right)\\ & +A_{23}\;\left(1\;–\;\exp\left(\;–\;t/\tau_{2\;\text{rise}}\;\right)\right)\exp\left(–\;t/\tau_{3\;\text{decay}}\right)\\ & +A_{24}\;\left(1\;–\;\exp\left(\;–\;t/\tau_{2\;\text{rise}}\;\right)\right)\exp\left(–\;t/\tau_{4\;\text{decay}}\right). \end{array}$$ (20)

As in Equation 16, in Equation 20, there are four lifetimes—two rises and two decays. Amplitudes are now independent. Functions of this kind will be referred to as non-convoluted below.

Equation 20 has eight independent parameters instead of seven in Equations 16, 19; however, it is much more flexible and clear. In Equation 20 (taking into account the special cases where some amplitudes are close to 0), it is possible to distinguish between the cases of Equations 17, 18. The presence of one-rise-two-decay or two-rise-one-decay sites can be identified using amplitude similarities. However, fitting the same experimental curve with four independent one-rise-one-decay functions (12 parameters in total) may prove to be overfitting.

Consequently, in this study, we used two kinds of functions: the type of functions from Equation 20 and the type of functions from Equation 16 (non-convoluted and convoluted, respectively). The functions are labeled by the number of rise-and-decay components. For instance, the pulse function is r1d1 (one-rise-one-decay), Equation 14 is r1d2 (one-rise-two-decays), Equation 15 is r2d1 (two-rise-one-decay), and so on. A full list is provided in Supplementary Data Sheet S2, Section SI.1.3.

From a practical point of view, it is convenient to start with the functions of Equation 16 kind (the convoluted ones, fewer parameters), find a stable solution, and express the solution in the form of the corresponding function of Equation 20 (the non-convoluted kind), and refit. As the functions of the convoluted kind have fewer degrees of freedom, in the present study, it was usually more difficult to find a solution using the functions of the non-convoluted kind from scratch—hence the two-step approach.

### 2.5 Numerical solutions of rate equations

Systems of rate equations were used to build a function (subroutine) that calculated the d*n*_i_/d*t* derivatives based on the current values of *n*_i_ (at a given time) and the considered process rates. Taking into account the derivative subroutine and rates, the equations were integrated using the scipy.integrate.odeint module from SciPy (SciPy, 2011) with default settings [which is a wrapper of the ODEPACK (Hindmarsh, 1983) library], and call the Livermore Solver for Ordinary Differential Equations (LSODE) Adams/Backward Differentiation Formula method with automatic stiffness detection and switching (Hindmarsh, 1983; Petzold, 1983). As a result of integration, time evolution curves were obtained for all levels of the model. The selected curves could thus be compared with their respective experimental counterparts. One can, therefore, treat the system of equations variationally, manipulating the values of the rates in order to minimize the residual between the simulated and the experimental curves. In such a case, integration is performed from scratch at each fitting step with appropriate parameters. The time axis is defined as an array of *t* values with a specified step. In the case where rates were found variationally, the time axis was the one from the experimental sample, extrapolated to *t* = 0 with the same step as in the experimental curve.

## 3 Results and discussion

### 3.1 Physical properties of the samples

According to X-ray diffraction data, the obtained materials corresponded to hexagonal *P 6*_*3*_ */m m c* (space group nr. 194) LaF_3_. In the material, cell angles α and β are 90°, while γ is 120°. The unit cell comprises two fluoride formula units, with one kind of La position and two kinds of F positions. The La atoms are surrounded by 11 F atoms in a D_3h_ local symmetry. The main C_3_ axis coincides with the cell Cartesian *z*-axis. Two fluoride atoms occupy the axial positions, three form an equilateral triangle in the equatorial plane, and the other six form two equilateral triangles in the planes parallel to the equatorial one. These triangles are rotated 30° with respect to the equatorial triangle around the C_3_ axis. The site has no inversion symmetry. As this geometry is not further discussed in this paper, the visualization is provided in Supplementary Data Sheet S2, Figure S3. Substitution of the dopant ions in the La sites is assumed.

Scherrer analysis estimates the average particle size to be approximately 50 nm. The XRD plots are shown in Supplementary Data Sheet S2, Figure S4. According to TEM, the materials are composed of well-defined nanocrystals. The widths start at 20–30 nm and span up to 50 nm. The lengths also start at 20–30 nm and span up to 80–100 nm. The material is thus a mixture of somewhat spherical particles and variable-length thick rods. Among other particles, rhombic-shaped nanocrystals (characteristic of hexagonal structures) are detectable. The TEM images are shown in Supplementary Data Sheet S2, Figures S5, S6.

Particle sizes allow for the estimation of surface site percentage. Depending on the crystal surface, two layers of metal sites correspond to 5–7 Å layer of atoms. For a spherical nanoparticle of 50 nm diameter, the outer layer of 0.6 nm (considered as the surface) would correspond to approximately 7% of the nanoparticle volume, which is considerable. A similar surface layer size was used by Vanetsev et al. (2017). On the other hand, as the metal nitrate solution was added dropwise to the ammonium fluoride solution, the initial precipitation occurred in the effective excess of the fluoride. It is thus likely that the surface metal cations are fully caped with fluoride anions and not with OH groups. The lack of capping agents and drying of the samples should have reduced the amount of OH groups and surface water as well. It is likely that surface quenching should not be strong in the materials. In addition, due to the different crystallization rates of different lanthanide fluorides (Gulina et al., 2017), the dopants might have a tendency to be located closer to the center of the particle (Gulina et al., 2017; Grzyb et al., 2013).

### 3.2 Peculiarities of the fitting process

In the case under study, there were basically two types of curves—decay curves and rise-and-decay curves. The decay curves are the sum of the following components: *A*_*i*_ exp (− *t*/*τ*_i_). Given the curve that is clearly decaying (that is, no apparent rise is present), the first guess was a monoexponential decay. If the result was unsatisfying (the residual was not “flat”), a second decay was added, then a third, and so on, until the residual contained no apparent signal. However, a curve that looks like a decay is actually the late part of a rise-and-decay. Fitting such a curve with a monoexponential decay will be unsatisfactory. With more components, any “hidden” (not obvious in shape) rising part of the curve would be visible as an exponential component with negative amplitude.

Given the curve with an apparent rise, the first guess was r1d1. The fitting process was then continued until a flat residual was obtained by adding more rise and/or decay components. In such a process, the experimenter’s intuition guides the process. It can be said that if the residual goes up at the beginning, it is probable that another decay component will be required, while a rise component will be required if the opposite is true. However, this was not the rule.

In many cases, it was impossible to correctly guess the entire curve. In such cases, the curve was cropped at some point. For instance, only the first 2 ms, 5 ms, or 10 ms were fitted with simple functions such as r1d1 or r2d1. In a separate fit, the last 10 ms, 20 ms, and 30 ms were fitted with mono- or biexponential decay. This step-by-step approach allowed for the correct detection of rise-and-decay lifetimes, as well as the number of the respective components. Then, taking into account the obtained values, a guess was constructed for the entire curve. It is essential that the resulting whole-curve lifetimes match (at least by the order of magnitude) the lifetimes of the partial fits. If the late part of the curve has monoexponential decay with a lifetime of, for example, 10 ms, then the entire curve fitted with a rise-and-decay function must exhibit a decay lifetime of approximately ∼10 ± 1–2 ms. A decay lifetime of ∼2–3 or 40–1,000 ms should raise suspicions.

It turned out that the decay components of the many-rise-many-decay functions cause the most problems. In other words, the rising part of the curves was relatively easy to guess and fit, while the decay parts were unconvincing. Thus, all of the fittings began with partial fits of the latter parts of the curves, where, in most cases, no rise was present—that is, where the curves appeared as decays *and* could be fitted with one to three exponential functions with positive amplitudes.

Finally, for some whole-curve fits with rise-and-decay functions, some of the resulting amplitudes were negative. Such a situation indicates an improperly selected type of component (lifetime) with a negative amplitude: the respective rise component (lifetime) must be, in fact, a decay component (lifetime) or *vice versa*. For example, an r2d3 fit exhibiting a negative amplitude for one of the decay components indicates that r3d2 must be used instead.

It also happened that some of the rise lifetimes approached some of the decay lifetimes. Both effectively cancel out, which in practice means that either the respective rise is redundant and should be removed; (for example, r2d2 should be used instead of r3d2), or both the respective rise and decay are redundant (e.g., r2d1 must be used instead of r3d2). Another indication of this situation is a rapid increase (especially between the consecutive refits) of the selected amplitudes, sometimes by several orders of magnitude, along with a synchronous change in some rise lifetime and some decay lifetime, both having similar and rather erroneous values (e.g., milliseconds or seconds, when microseconds were expected).

Sometimes, finding a good fit meant looking for trends and patterns. Similar systems must result in similar kinetics. Particularly, the Eu^3+^ emission at a given wavelength must exhibit similar rise-and-decay lifetimes (at least of the same order of magnitude), as well as the same (or similar) numbers of rise-and-decay components. In most cases, such correspondence occurred naturally: a series of good fits *post factum* turned out to exhibit trends and similarities in the values of the variables. However, in the selected cases, there were several options for good fits, while only one had to be selected as “correct”—the one most similar to the other fits of the profiles of emission at the wavelength in question.

### 3.3 The LaF_3_:Gd^3+^ sample

One pulsed-excitation measurement was performed for the LaF_3_:Gd^3+^ sample with λ_ex._ = 272 nm and λ_em._ = 312 nm. The curve was a rise-and-decay type. After the excitation pulse, some of the Gd^3+^ ions are in the ^6^I_17/2, 15/2, 13/2, 11/2, 9/2,7/2_ excited states, which for the convenience of readers will be abbreviated and referred to here as the ^6^I manifold/state/level. Radiative decay to the ^8^S_7/2_ ground state or a non-radiative process to the ^6^P_7/2,5/2,3/2_ levels (^6^P manifold) may occur. Because the energy gap in the latter process is neither large nor small (∼4,000 cm^−1^), it is difficult to say which process will prevail; however, it is clear that the latter process occurs, resulting in the ^6^P manifold population and the ^6^P → ^8^S_7/2_ emission at 312 nm.

Given the populating process: Gd^3+^: ^6^I → ^6^P, the kinetics of the ^6^P manifold must follow a rise-and-decay pattern, as discussed in Section 2.3.2:

$$I=I_{0}+A_{0}\;\left(1\;–\exp\left(–\;\left(t\;–\;t_{0}\right)\;/\;\tau_{\text{rise}}\right)\right)\exp\left(–\;\left(t\;–\;t_{0}\right)\;/\;\tau_{\text{decay}}\right).$$ (21)

Here, *τ*_rise_ = 1/(*W*_rad._ + *W*_nrad._), τ_decay_ = 1/*W*_rad._, W_rad._ is the rate of the ^6^P → ^6^S_7/2_ radiative relaxation, and *W*_nrad._ is the rate of the ^6^I → ^6^P non-radiative process. The vertical (intensity) offset *I*_0_ and horizontal (time) offset *t*_0_ are also featured, giving the equation actually used in the fitting.

**FIGURE 2:**
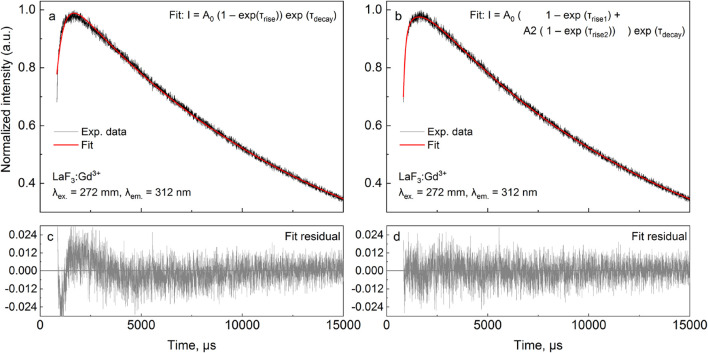
LaF_3_:Gd^3+^ 312 nm emission under 272 nm pulsed excitation, with two different fits.

The r1d1 function (Equation 21, solution S1; Table 1) can be fit to the experimental curve, obtaining a rise time of 363 μs and a decay time of 12,839 μs. However, the result (Figures 2a, c) indicates that a more complex function should be used. Note the wavy residual in Figure 2c. Such a residual clearly contains some signal (significant within 800–5,000 μs after the pulse), indicating an incomplete (bad) fit. A good fit can be unambiguously obtained using the following equation (r2d1 function), with the rise lifetimes of 98.7 µs and 700 µs (the corresponding amplitude ratio was ∼1:0.235, contributions of 81% and 19%, respectively) and the 12,700 μs decay (solution S2, Table 1):

$$I=I_{0}+A_{0}\left[1\;–\exp\left(–\;\left(t\;–\;t_{0}\right)\;/\;\tau_{\text{rise}1}\right)+A_{2}\;\left(\;1\;–\exp\left(–\;\left(t\;–\;t_{0}\right)\;/\;\tau_{\text{rise}2}\right)\right)\right]\exp\left(–\;\left(t\;–\;t_{0}\right)\;/\;\tau_{\text{decay}}\right).$$ (22)

**TABLE 1:** Rise-and-decay lifetimes (μs) of the LaF_3_:Gd^3+^ sample. Amplitudes are given in arbitrary units (curves have been normalized). Values in bold are the most similar (see text)

x_0_, μs	*A*_0_/*A*_1_	*A*_2_	τ_rise1_, μs	τ_rise2_, μs	τ_decay1_, μs	τ_decay2_, μs	Equation	Solution #
367	1.149		362.5		**12,839**		Equation 21	S1
692	0.913	0.235	98.7	700	12,700		Equation 22	S2
692	0.914	0.214	99.3	703	12,466	13,748	2✕ Equation 21	S3
692	0.914	0.214	98.9	705	**12,875**	11,975	2✕ Equation 21	S4
693	0.913	0.215	98.6	700	**12,715**	**12,636**	2✕ Equation 21	S5

The corresponding residual in Figure 2d contains no noticeable signal—at least with amplitudes significantly larger than the noise. It can also be seen in Figure 2b that Equation 22 sits on the experimental points much better. Thus, there are two exponential components in the rise in Figure 2. One of them must be the Gd^3+^ ^6^I → ^6^P relaxation, while the nature of the second is discussed below. All of the fits discussed below resulted in the overall picture shown in Figures 2a, c or Figures 2b, d and were considered as bad or good fits, respectively.

There is another, more complex way to fit a given kinetics. Namely, two independent r1d1 equations (Equation 21) can be used. In this case, the *I*_0_ and *A*_0_ variables are replaced by *I*_1_, *A*_1_, and I_2_, *A*_2_ in the first and second functions, respectively. The S3 solution (Table 1) was obtained from the S1 solution by adding another r1d1 component. The associated solution S4 was obtained from S3, using the τ_decay2_ < τ_decay1_ in the initial guess. The fit of the S4 is only as good as the S3. Finally, the solution S5 guess was obtained from solution S3 by swapping the decay lifetimes in the two components and setting both amplitudes to 0.5. Once again, it was a good fit of the Figures 2b, d kind. The resulting decay lifetimes are very close and consistent with the S2 solution. The decay lifetimes of solutions S3 and S5 differ from the decay lifetimes of solutions S1 and S2.

In solution S4, *τ*_decay1_ is very close to *τ*_decay1_ in S1, which means that *τ*_decay2_ of solution S4 is redundant. Thus, this solution is incorrect. This was shown as an example of an error. Another bad solution is S5: its similar values of *τ*_decay_ indicate that only one decay component had to be used in the summary function (i.e., r2d1 had to be used), which is consistent with solution S2.

Solution S3 provides a different interpretation than S2. In S2, the two species of Gd^3+^ are considered to interact with each other, as described in the next section. Two independent equations are used in S3, both corresponding to an isolated Gd^3+^ site. They can be considered a surface site with faster ^6^I → ^6^P relaxation due to the presence of OH groups and a shorter radiative lifetime originating from the less symmetric coordination geometry (τ_rise_ = 99.3 μs, τ_decay_ = 12,466 μs). The second site, with a longer radiative lifetime and weaker non-radiative quenching (τ_rise_ = 703 μs, τ_decay_ = 13,748 μs), must be the bulk site. However, this interpretation implies that the ratio of surface to bulk sites is 0.914:0.214 ≈ 4.27:1. In other words, 81% of emissions come from the surface sites, which is possible albeit unexpected. However, considering the S4 and S5 solutions, the S3 solution is likely a redundant solution and not a true indication of the dominant surface sites.

It is worth noting that the S2 and S3 fits are equally good. The S2 solution contains six fit parameters, while S3 contains seven fit parameters. From the point of view of numerical complexity, S2 is preferred. However, the two solutions assume different physics of the process. In S2, there is only one decay lifetime, while in S3, there are two. As mentioned in Section 3.2, of two equally good solutions, the one with the smaller number of decay components should be selected.

This section illustrates the care that should be taken when fitting multiexponential functions. The functions are guess-sensitive and (to some extent) flexible, providing opportunities for mistakes and misinterpretations (including intentional ones). Care should be taken during fitting routines, during interpretation, and when describing the result. In particular, as we illustrate here, **it is a good and recommended practice to show the residuals** (Debasu et al., 2011) and **describe the fitting peculiarities**.

#### 3.3.1 Model system: Gd^3+^-Gd^3+^ energy transfer

**FIGURE 3:**
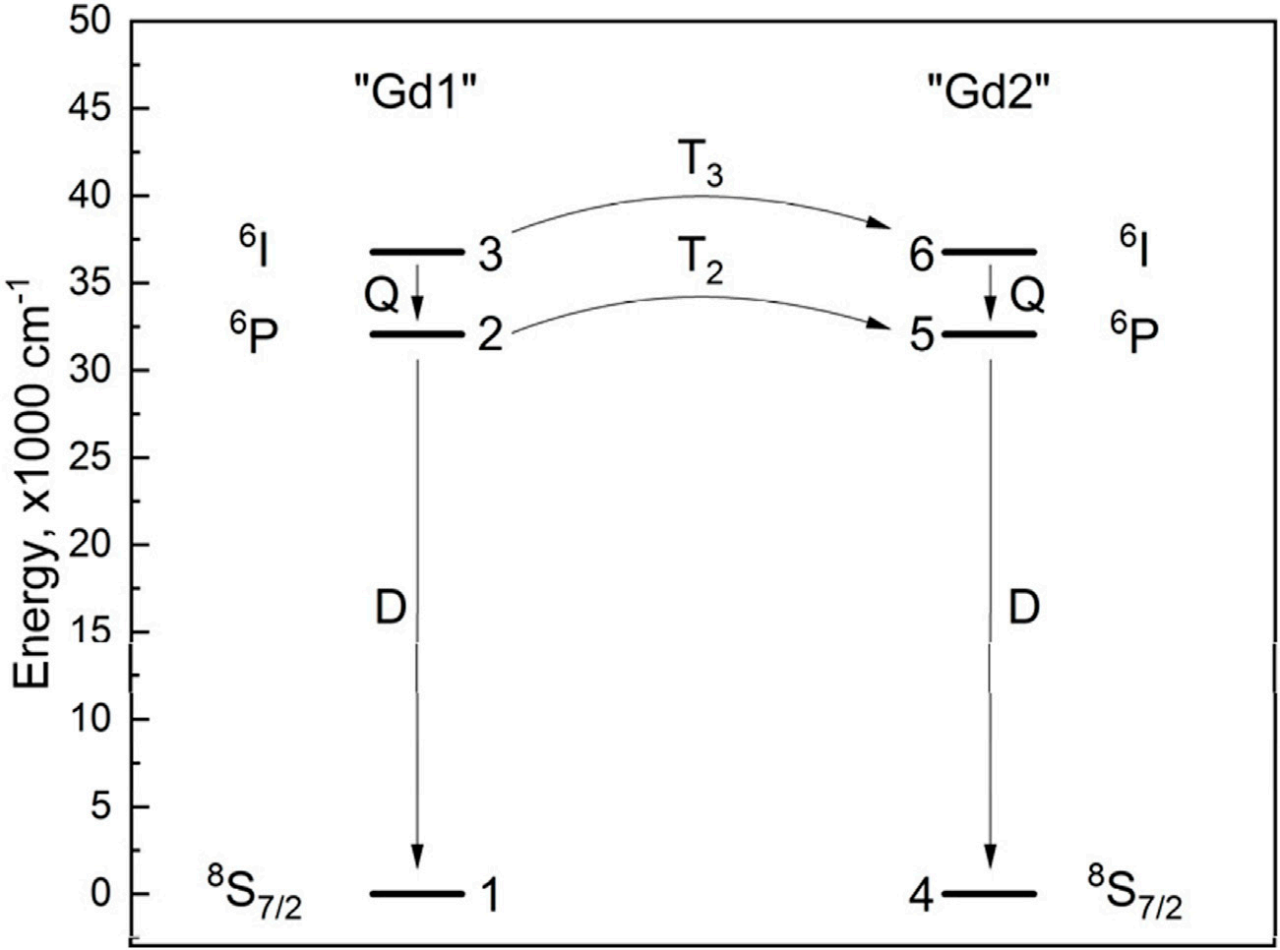
Energy level diagram and energy transfer scheme of Gd^3+^-Gd^3+^.

Another explanation of the observed kinetics is the interaction between two groups of Gd^3+^ ions (Gd–Gd pair are also plausible) via non-radiative energy transfer. In order to describe such interactions, the energy level scheme must include at least two “Gd” species. Otherwise, there would be no energy transfer. The simplest acceptable scheme is shown in Figure 3. In LaF_3_, all crystallographic sites for La^3+^ (and, therefore, most likely Gd^3+^) are the same. Consequently, the non-radiative relaxation rate Q from the ^6^I manifold to the ^6^P manifold of Gd^3+^ must be the same in both species. The radiative relaxation rates D (^6^P → ^8^S_7/2_) are also the same. The T_2,3_ processes are energy transfers between the corresponding levels of “Gd” species; T_2_ and T_3_ may be different.

#### 3.3.2 Constructing the Gd^3+^–Gd^3+^ rate equations

A system of six rate equations corresponding to six energy levels (Figure 3) was constructed. Below, *n*_*i*_ are level populations. Energy transfer processes were considered bidirectional. The energy levels of two different Gd^3+^ ions are in perfect resonance; thus, the back transfer (“Gd2” to “Gd1”) must have the same rate constant as the direct process (“Gd1” to “Gd2”). The rates of elementary energy transfer depend on the corresponding rate constants and the populations of two energy levels: the initial donor level and the initial acceptor level. For instance, W_T3_ is the sum of the direct transfer rate from level 3 to level 6 and the back transfer from level 6 to level 3. Thus, W_T3_ (from the point of view of “Gd1”) equals *T*_3_ *n*_3_ *n*_4_ − *T*_3_ *n*_6_ *n*_1_ = *T*_3_ (*n*_3_ *n*_4_ − *n*_6_ *n*_1_).

$$W_{T3}=T_{3}\;\left(\;n_{3}\;n_{4}\;–\;n_{6}\;n_{1}\;\right).$$ (23)

$$W_{T2}=T_{2}\;\left(\;n_{2}\;n_{4}\;–\;n_{5}\;n_{1}\;\right).$$ (24)

$$\partial n_{1}/\partial t=D\;n_{2}+W_{T2}+W_{T3}.$$ (25)

$$\partial n_{2}/\partial t=–\;D\;n_{2}+Q\;n_{3}\;–\;W_{T2.}$$ (26)

$$\partial n_{3}/\partial t=–\;Q\;n_{3}\;–\;W_{T3.}$$ (27)

$$\partial n_{4}/\partial t=D\;n_{5}\;–\;W_{T2}\;–\;W_{T3.}$$ (28)

$$\partial n_{5}/\partial t=–\;D\;n_{5}+Q\;n_{6}+W_{T2}.$$ (29)

$$\partial n_{6}/\partial t=–\;Q\;n_{6}+W_{T3.}$$ (30)

#### 3.3.3 Initial values of the rate equations

Equations 23, 24 make it clear that energy transfer rates are proportional to level populations and rate constants. Although the populations of individual particular levels at a given time are defined by rate equations, the initial values can either be specified and fixed or treated variationally. Rate constants were variational by definition. Making the initial populations variational would result in competition between parameters: the same rates can be obtained by an infinite number of combinations of different rate constants and initial values. In order to avoid it, we froze the initial populations.

The species “Gd1” was assumed to be Gd^3+^ ions that were excited by a pulse of radiation (i.e., laser pulse). Thus, the initial values of *n*_1–3_ were 0, 0, 1, corresponding to the group of Gd^3+^ ions in their ^6^I manifolds. The species “Gd2” was assumed to be Gd^3+^ that was not excited by the pulse. The initial values of *n*_4-6_ were 2242880, 0, 0, corresponding to Gd^3+^ ions in their ^8^S_7/2_ ground states.

The value of 2,242,880 was selected as follows. Our laser was transmitting an average of approximately 0.1 mJ of energy per pulse. The energy of the 272 nm transition is 7.3 × 10^−19^ J. One pulse is thus enough to excite 1.37 × 10^14^ Gd^3+^ ions, or 2.27 × 10^−10^ mol. A sample of ∼0.1 g corresponds to approximately 5.1 × 10^−4^ mol of LaF_3_:1%Gd (molar mass 196.0841 g/mol) or 5.1 × 10^−6^ mol of Gd^3+^ (1% doping). One pulse can thus excite one Gd^3+^ ion in 22,429, assuming 100% absorption and using unrounded numbers. However, because the Gd concentration is only 1%, and the sample can scatter and reflect light, we have assumed an optimistic excitation ratio of one ion in 2,242,880.

#### 3.3.4 Output kinetics

Given a set of input parameters (amplitude A and rate constants expressed as lifetimes, *τ*_*X*_ = 1/*X*: *τ*_*D*_, *τ*_*Q*_, *τ*_*T*2_, *τ*_*T*3_), our model returned the temporal dependence of *n*_5_ (resulting from the numerical solution of Equations 23–30), multiplied by A. The resulting curve was compared to the experimental one, with the difference between them accounting for the residual. The sum of squares of the residual was minimized by variational modification of five parameters: the four lifetimes and the amplitude. It is worth noting that the exponential rise-and-decay fit also had five parameters. The population of level 5 was compared to the experiment because its peak value is larger than the peak value of *n*_2_; neither *n*_2_ nor *n*_2_+*n*_5_ curves are able to reproduce the experimental kinetics; *n*_2_ decays very fast. Although this approximation is a stretch (some part of the “^6^P” population is ignored), it gives a reasonable result. It is worth noting that on the *n*_2_+*n*_5_ curve, the result of the ^6^P → ^6^P energy transfer is effectively eliminated.

#### 3.3.5 Fitting the parameters of rate equations

During the fitting procedure, variational parameters were allowed to change without restrictions. The rate equations did not contain any parameters other than those described above. In this way, the equations were kept as flexible as possible while representing a model system.

Fitting with the rate equation system as a function with variational parameters seemed to be more sensitive to initial guesses than the multiexponential fits. However, some stable solutions were obtained unambiguously. Many of them were of the kind shown in Figure 2a (“bad”). Such solutions correspond to a simpler rate equation system in which energy transfer processes are irrelevant, and the emission rise is explicitly determined by the ^6^I → ^6^P relaxation rate. The indistinguishable fit can be obtained by the rise-and-decay function, Equation 1. Noteworthy, the values *τ*_*Q*_ from such fits are similar to the *τ*_*rise*_ obtained from Equation 1. This is not surprising, as the rise-and-decay function is an analytical solution to a rate equation system representing only the “Gd1” species (Equations 23–30) as an isolated system without *W*_*T*2,*T*3_.

In order to come from a bad fit to a good one, large values (1 × 10^6^–1 × 10^8^ μs) of *τ*_*T*2_ and *τ*_*T*3_ were used in the guess. After several attempts, the fit converged to a stable, reproducible solution. The following values were found: *τ*_*D*_ = 12,700 μs, *τ*_*Q*_ = 97.95 μs, *τ*_*T*2_ = 1,570 s and *τ*_*T*3_ = 52.2 s. These values would correspond to energy transfer rate constants of 0.000637 s^−1^ and 0.0192 s^−1^ if the energy is transferred from a single Gd^3+^ ion to 2,242,880 nearby ions. Alternatively, we can think of a pair of ions: in that case, the constants must be 1,429 s^−1^ (^6^P ↔ ^6^P) and 42955 s^−1^ (^6^I ↔ ^6^I).

The rise lifetimes obtained from Equation 22 can therefore be assigned as follows. The dominant component of 98.7 μs is the Gd^3+^ ^6^I → ^6^P relaxation rate. The 700 μs component results from the rate equation system as a whole (an emergent component/rate) and does not correspond to a specific transition. This occurs as a result of the energy transfer interaction of excited Gd^3+^ ions with some neighboring Gd^3+^ in their ground states.

#### 3.3.6 The improbable Ce^3+^ contamination

An alternative explanation for the additional rise component would be the presence of a small amount of Ce^3+^ in the LaF_3_:Gd^3+^ sample. In 99.99% La_2_O_3_, the most likely impurity is Ce, whose content is less than 0.01%. Ce^3+^ was excluded based on the excitation spectra of the sample, which do not contain broad bands in the 200–350 nm range, characteristic of the f-d absorption of Ce^3+^. Nerveless, we check this possibility using rate equations, assuming the presence of a Ce^3+^ ion near the Gd^3+^ ion, with a subsequent Gd^3+^ ^6^I → Ce^3+^ f-d → Gd^3+^ ^6^P energy transfer. Model details are provided in Supplementary Data Sheet S2, Section SI.4. Briefly, a model similar to the Gd-Gd pair model above was used, and the parameters of the rate equations were fitted to the experimental kinetics. Many fits failed; that is, they coincided with the result shown in Figure 2a.

Although some good fits (Figure 2b) were obtained, the resulting parameters corresponded to a high content of Ce^3+^, as well as no emission from it—both conditions are highly unlikely and illustrate the inconsistency of the Ce^3+^ contamination assumption. Taken simply, the empty excited level of the allowed d-f transition of Ce^3+^ at energy similar to the Gd^3+^ ^6^P ↔ ^8^S_7/2_ transition should quench the former rather than sensitize it. Therefore, Ce^3+^ contamination cannot explain the 700 μs rise component of the Gd^3+^ ^6^P → ^8^S_7/2_ emission kinetics at 272 nm excitation.

Based on this result and the excitation spectra, Ce^3+^ contamination of the LaF_3_:Gd^3+^ sample was excluded.

### 3.4 The LaF_3_:Ce^3+^,Gd^3+^ sample

**FIGURE 4:**
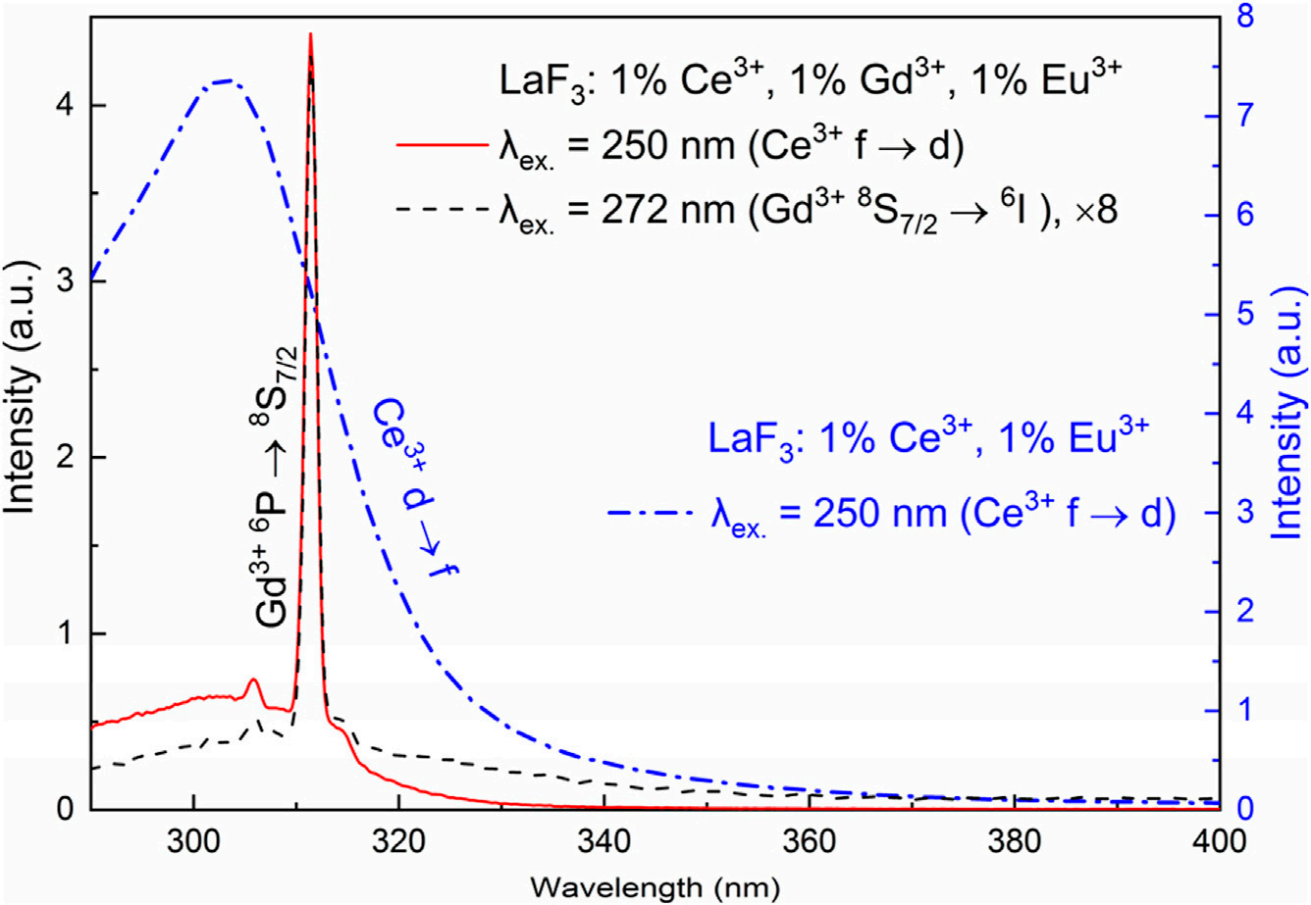
UV emission of LaF_3_:Ce^3+^,Gd^3+^,Eu^3+^ (*y*-axis at the left) at 250 nm and 272 nm excitation and LaF_3_:Ce^3+^,Eu^3+^ (*y*-axis at the right) at 250 nm excitation.

The sample, excited in either the 272 nm f-f band of Gd^3+^ or the 250 nm f-d band of Ce^3+^, shows a sharp emission peak at 312 nm. Much weaker, broad emission bands are observed in the range of 290–400 nm. The bands coincide with the Ce^3+^ d-f emission in LaF_3_:Ce^3+^,Eu^3+^ (Figure 4). Consequently, in the case of 250 nm excitation, not all the energy from Ce^3+^ is transferred to Gd^3+^. Some of the energy is emitted as light, which is not surprising given the allowed nature of the d-f transition of Ce^3+^ and its very short lifetime. In the case of 272 nm emission, the broadband emission indicates that some portion of Ce^3+^ ions become excited, either by direct absorption of the excitation light or by energy transfer.

In contrast to LaF_3_:Gd^3+^, in LaF_3_:Ce^3+^,Gd^3+^ (excited both in the 272 nm f-f band of Gd^3+^ or in the 250 nm f-d band of Ce^3+^, λ_em._ = 312), there are several decay components, while the rise is completely missing. Both decay profiles can be fitted with three or four exponential components; see Table 2.

**TABLE 2:** Decay lifetimes (μs) of LaF_3_:Gd^3+^,Ce^3+^. Amplitudes are given in arbitrary units (curves have been normalized).

t_0_, μs	*I*_0_	*A*_1_	τ_1_	*A*_2_	τ_2_	*A*_3_	τ_3_	*A*_4_	τ_4_
λ_ex._ = 250 nm									
**831.000**	**−0.0166141**	**0.879166**	**12,634**	**0.087047**	**4,410.95**	**0.0204805**	**275.87**	**0.025874**	**35.4218**
691.259	−0.0166254	0.888588	12,636.8	0.0901167	4,426.67	0.03223	286.956	1.05996	37.7924
829.801	−0.0163597	0.886326	12,574.8	0.0815107	4,086.46	0.0351387	137.13		
0.000	−0.0163663	0.946582	12,576.3	0.0999857	4,095.64	13.5707	139.195		
λ_ex._ = 272 nm									
**831.000**	**−0.011197**	**0.789245**	**12,416.8**	**0.100361**	**3,934.44**	**0.0485335**	**828.879**	**0.0638484**	**57.897**
533.131	−0.0111989	0.808329	12,417.4	0.108239	3,938.92	0.0694935	831.094	10.7413	58.1124
788.438	−0.00966774	0.821315	12,099	0.10325	2,331.42	0.096255	121.436		

Bold values indicate “correct” fits.

The fitting results are quite sensitive to the fitting procedure. In particular, there are several options for treating the time axis offset. Recorded curves do not start at zero time; the first point has a time value of 831 μs. One of the options is to not use time offset whatsoever, simply because each piece of the exponential decay profile is the same decay profile, except for amplitude. However, this logic applies under ideal conditions, where noise is exactly 0. Because experimental noise is present, a short-lived component with a lifetime of, say, approximately 50 μs would be practically unnoticeable at 831 μs and would require a ridiculously large amplitude to have any effect. Another option is a variable time offset. However, this option introduces an undesirable degree of freedom, which also renders lifetimes and amplitudes dependent on its value, and results in a form of internal dependence between parameters. Therefore, we used a fixed offset of 831 μs; that is, we assumed that the experimental decay profiles start at the time point of 831 μs. Such fits are shown in bold in Table 2 and are considered “correct.” Note the differences with the other options. This approach also eliminates ambiguity in the number of components. With variable offset, as well as without offset, it is possible to achieve an acceptable fit with only three components. With a fixed offset of 831 μs, four components are clearly visible. We emphasize that all the fits were good, that is, stable, reproducible (although guess-dependent), and resulting in the flat residual.

In the LaF_3_: 1%Eu^3+^, 1%Ce^3+^ sample obtained in the same series of samples as the discussed LaF_3_: 1%Gd^3+^, 1%Ce^3+^ sample, a broad band of the Ce^3+^ f-d emission is observed in the 280–320 nm range, peaking at 303 nm. Consequently, a strong overlap of the Ce^3+^ f-d excitation band with the 272 nm ^6^I ↔ ^8^S_7/2_ band of Gd^3+^ is expected. The Ce^3+^ f-d emission overlaps with the 312 nm ^6^P ↔ ^8^S_7/2_ band of Gd^3+^. Thus, the following energy transfer is possible: Gd^3+^ ^6^I → Ce^3+^ f-d → Gd^3+^ ^6^P. This mechanism is supported by the fact that the 272/312 nm decay profiles of Gd^3+^ in LaF_3_:Gd^3+^,Ce^3+^ do not show any rise, indicating the involvement of Ce^3+^ in the Gd^3+^ ^6^P dynamics. Energy transfer processes with Ce^3+^ as one of the parts must be very fast due to the allowed f-d transitions of the latter.

The Ce^3+^ f-d radiative decay is not forbidden and is characterized by a very short lifetime [we used the value of 29.2 ns (Kroon et al., 2014)], while the Gd^3+^ radiative lifetime is much longer, approximately 12.5–12.6 ms. In other words, the decay rate of the Ce^3+^ f-d transition is approximately half a million times greater than the radiative decay rate of Gd^3+^. Roughly the same ratio is expected for the respective absorption probabilities. Consequently, when LaF_3_:Ce^3+^,Gd^3+^ is excited at 272 nm, mostly Ce^3+^ ions are excited. At 250 nm excitation, only Ce^3+^ ions are excited. Thus, both cases must be characterized by very similar overall kinetics despite the fact that particular excited atoms and groups of atoms may depend on the excitation wavelength. It is worth noting that the excited spot of the sample also varied slightly, depending on the wavelength. Thus, “correct” decay fits satisfy both quality and consistency requirements; that is, the two excitation cases exhibit similar lifetimes and similar amplitudes.

#### 3.4.1 Ce^3+^-Gd^3+^ model system and rate equations

**FIGURE 5:**
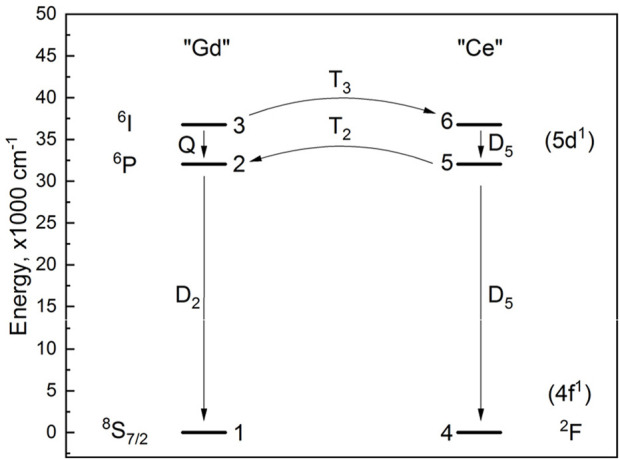
Ce^3+^-Gd^3+^ energy transfer scheme.

Several systems of rate equations were established to analyze the nature of the observed decay components. The simpler one consisted of one “Gd” species and one “Ce” species, as shown in Figure 5.

The rate equations are given in Equations 31–38:

$$W_{T3}=T_{3}\;\left(\;n_{3}\;n_{4}\;–\;n_{6}\;n_{1}\;\right).$$ (31)

$$W_{T2}=T_{2}\;\left(\;n_{2}\;n_{4}\;–\;n_{5}\;n_{1}\;\right).$$ (32)

$$\partial n_{1}/\partial t=\left(–\;P_{\text{Gd}}\;n_{1}\right)+D_{2}\;n_{2}+W_{T2}+W_{T3}.$$ (33)

$$\partial n_{2}/\partial t=–\;D_{2}\;n_{2}+Q\;n_{3}\;–\;W_{T2.}$$ (34)

$$\partial n_{3}/\partial t=\left(+P_{\text{Gd}}\;n_{1}\right)\;–\;Q\;n_{3}\;–\;W_{T3}.$$ (35)

$$\partial n_{4}/\partial t=\left(–\;P_{\text{Ce}}\;n_{1}\right)+D_{5}\;n_{5}\;–\;W_{T2}\;–\;W_{T3}.$$ (36)

$$\partial n_{5}/\partial t=–\;D_{5}\;n_{5}+D_{5}\;n_{6}+W_{T2.}$$ (37)

$$\partial n_{6}/\partial t=\left(+P_{\text{Ce}}\;n_{1}\right)\;–\;D_{5}\;n_{6}+W_{T3}.$$ (38)

Both excited states of Ce^3+^ have the same relaxation rate of *D*_5_, which was fixed at the inverse of 29.2 ns (Kroon et al., 2014). The terms in brackets correspond to optional pump terms. The treatment of the pump underwent a slight evolution. Initially, in the case of steady-state simulations of Ce^3+^ excitation, *P*_Ce_ was changed variationally, while *P*_Gd_ was zero. In the case of steady-state simulations of Gd^3+^ excitation, *P*_Ce_ was changed variationally, while *P*_Gd_ was kept equal to *P*_Ce_·*D*_2_/*D*_5_. In such a way, both pump rates were changed, while their ratio remained the same as the ratio of the corresponding radiative relaxations. This assumption was made due to the fact that both energy transfer rates and absorption rates are proportional to the electric dipoles involved in the transitions. However, *D*_2_ is an emission rate at level 2 (^6^P) of Gd^3+^, while the Gd^3+^ pump populated level 3 (^6^I). Judging by Carnall’s tables and the results of the analysis of the Gd-Gd system, both manifolds have rather different electric dipoles. The *P*_12_ and *P*_13_ pump rates are proportional to the Gd^3+^ ^8^S_7/2_ → ^6^P and ^8^S_7/2_ → ^6^I transition dipoles, which should be approximately proportional to the *T*_2_ and *T*_3_ energy transfer rates. Thus, *P*_2_/*P*_5_ ≈ *D*_2_/*D*_5_, *P*_3_/*P*_2_ ≈ *T*_3_/*T*_2_, *P*_3_/*P*_5_ = (*P*_3_/*P*_2_) (*P*_2_/*P*_5_) ≈ (*T*_3_/*T*_2_) (*D*_2_/*D*_5_). Ultimately, *P*_3_ = *P*_Gd_ was defined as *P*_5_ (*T*_3_/*T*_2_) (*D*_2_/*D*_5_), *P*_5_ = *P*_Ce_. In this way, the initial populations of the levels depended on the very same rate parameters as their following post-excitation evolution, giving a self-consistent model.

#### 3.4.2 Ce^3+^-Gd^3+^ initial values

In order to simulate a population of levels after a short pulse, the corresponding system of ordinary differential equations (ODE, Equations 31–38) was solved in a steady-state mode, with a pump at Gd or Gd and Ce (corresponding to experimental excitation at 272 nm or 250 nm). In the beginning, all excited levels had populations of zero. The system was allowed to evolve for 8 ns (laser pulse length) with a time grid of 0.4 ps. Such a solver subroutine was embedded in a fitting loop. The Ce and Gd pump rates were calculated as described in the previous section. The pump rate was fitted so that the summary population of excited levels after 8 ns was 1. Once again, this approach “evolved” in trial-and-error tests of many different approaches to the initial values and has proven to be the best.

In this system, the assumption from Section 3.3.3 also applied: due to the limited pulse energy, most of the ions remained in their ground states. Under 272 nm excitation, one atom in 2,242,880 was getting excited. At 250 nm, due to the higher energy of the transition, one atom in 2,440,104 was getting excited. Thus, a compromise (average) value of 2,341,492 was utilized so that both 272 nm and 250 nm excitation channels could be used in the simulation. The energy transfer rates in Table 3 correspond to this donor–acceptor ratio.

**TABLE 3:** Fitting results for the LaF_3_: 1%Gd^3+^, 1%Ce^3+^ sample, selected parameters.

λ_ex._, nm	*A*_*ODE*_	*X*_12_	*τ*_*nrad*_, μs	*τ*_*rad*_, μs	*T*_2_, s^−1^	*T*_3_, s^−1^	*X*_P2_
250	6,735	0.0485	100	12,982	7.40·10^−5^	2.79 × 10^−1^	0.0150
272	800	0.492	104	12,799	1.37·10^−4^	1.90 × 10^−1^	0.0128
250, 272	6,944	0.0430	104	13,094	8.94 10^−5^	2.21 × 10^−1^	0.0189
	677	0.574					

#### 3.4.3 ODE fitting

Section 3.4.2 referred to a single step of fitting. In each fitting step, for a specific set of *A*, *D*_2_, *Q*, *T*_2_, *T*_3_ parameters (where A is the emission amplitude), the following took place.

a. The post-pulse level populations were prepared as described in Section 3.4.2 by fitting the pump rate to obtain the sum of excited state populations equal to unity;
b. The post-pulse populations were used as the initial populations in the same ODE system, this time without the pump, simulating post-pulse relaxation;
c. The vector of values representing the population of the level 2 (Gd ^6^P) after the pulse (at the same time grid as the experimental values) was multiplied by A;
d. The difference between the result of step c. and the experimental values was the residual.

The residual was minimized by variational changes in *A*, *D*_2_, *Q*, *T*_2_, *T*_3_. Therefore, throughout the entire procedure, a set of parameters was sought to ensure the best fit of the experimental and theoretical time evolutions of the Gd^3+^ 312 nm emission.

#### 3.4.4 Fitting results

After substantial tests, models with one Ce species and one Gd species (with different parameters) were discarded as not resulting in the observed complexity of the decay patterns. However, such a system supports the idea that Ce^3+^ ions mediate the population of the Gd^3+^ emitting level through a Gd^3+^ ^6^I → Ce^3+^ → Gd^3+^ ^6^P energy transfer loop with a fast relaxation between Ce^3+^ levels. In such a loop, the Ce^3+^ levels are assumed to have the same energies as the Gd^3+^ levels, resulting in perfect resonance conditions. The perfect resonance has been experimentally confirmed on the basis of the spectral overlap mentioned above. Ce^3+^ also acts as a quencher of the 312 nm emission. In such a system, the radiative decay rate at 312 nm is defined by the Gd^3+^-Ce^3+^ distance, while the kinetics of the 312 nm emission is a monoexponential decay with a negligibly short rise. When the Gd^3+^-Ce^3+^ distance is “moderately large,” the species interact weakly, with a long (ms range) decay of the 312 nm emission. When the distance is “short,” the 312 nm emission is efficiently quenched and decays quickly (ns–μs range). However, we did not estimate the actual distance; we only analyzed the energy transfer rates.

In order to solve the experimental pattern, an alternative model was established, comprising two Ce–Gd pairs, which are characterized by the same decay rates in Gd and Ce, with the Ce–Gd energy transfer rates being proportional to each other—assuming the same chemical environment and different Ce–Gd distance for both pairs. In particular, for Pair1, the rates were *T*_2_ and *T*_3_, while for Pair2, the respective rates were *X*_*P*2_·*T*_2_ and *X*_*P*2_·*T*_3_. It was also assumed that ions do not interact between pairs. In other words, in Pair1, Ce1 only interacts with Gd1, while in Pair2, Ce2 only interacts with Gd2.

The amount of each pair was controlled by another variational parameter, *X*_12_. The amplitude of Pair1 was *A*·*X*_12_, while the amplitude of Pair2 was *A*·(1 − *X*_12_). Each pair had its own independent system of ODE. Thus, in each fitting step, the steps in Section 3.4.2 and Section 3.4.3 were performed separately for each pair. The sum of the two obtained level 2 kinetics was compared to the experiment, producing a single vector of residual.

With this two-pair system, good fits can be obtained for part of the curves with the first 400 μs cropped. Alternatively, it is also possible to obtain a good fit on all the data with additional single-exponential decay. In other words, the model suggests that the observed kinetics mainly originates from two kinds of Ce–Gd pairs, of which 5%–7% are shorter-distance pairs, and the rest are longer-distance pairs. However, there is a small percentage of very-short-distance pairs. In other words, most of the dopants are not agglomerated.

It is clear from Table 3 that both fits give similar values for the energy transfer rates. The non-radiative and radiative decay rates of Gd^3+^ levels correspond to the data LaF_3_: 1%Gd^3+^ very well. It is worth noting that the lifetimes in Table 3 do not correspond to the multiexponential decay fit lifetimes, except for the ∼13 ms τ_rad_. Thus, other multiexponential lifetimes are emergent; that is, they result from the interacting system as a whole and not from a particular isolated emitting ion or transition.

The curves resulting from 250 nm to 272 nm excitation differ significantly in *X*_12_, that is, in the fraction of Gd–Ce pairs with shorter distances. In the former, it is approximately 4.6%, while in the latter it is 49%. The values of the *X*_P2_ (energy transfer ratio) in both solutions are similar, indicating similar kinds of pairs.

Noteworthy, the *A*_*ODE*_ values approximately correspond to the experimental ratio of the LaF_3_: 1%Ce^3+^, 1%Gd^3+^, 1% Eu^3+^ emission spectra of the sample at 250 nm and 272 nm excitation, with the emission at 272 nm excitation being roughly eight times less intense (Figure 4).

#### 3.4.5 Two-curve fits

From Table 3, it is clear that the emission profiles under 250 nm and 272 nm excitations result in slightly different fitted parameters. This is acceptable as the two emissions refer to slightly different areas of the actual sample (the excitation laser focal point was dependent on the wavelength). On the other hand, both curves still refer to the same sample. We have thus tried to find a solution that would correspond to two curves at the same time. The same procedure as in Sections 3.4.1–3.4.3 was used, with one exception: the ODE systems for Gd-only excitation and Gd-Ce excitation were solved in parallel (with the same set of *A*, *D*_2_, *Q*, *T*_2_, *T*_3_, *X*_*P*2_ being found variationally), and two theoretical temporal evolution curves were compared with the experimental emission decay profiles, with excitation at 272 nm and 250 nm, respectively. At each step, the two residual vectors were concatenated, and the total residual was minimized with respect to the *A*, *D*_2_, *Q*, *T*_2_, *T*_3_, *X*_*P*2_ parameters. This approach resulted in a worse but acceptable solution, consistent with the conclusion from Section 3.4.4.

We attempted to make such a fit with *X*_12_ as another common parameter, but this approach resulted in the fit converging with solutions similar to either of the two one-curve solutions. Apparently, the two irradiated areas differ significantly in the ratio of the pairs (*X*_12_), and no intermediate solution is possible. This means that the proposed set of rate equations and fitting approach (Sections 3.4.1–3.4.3) can potentially be used in dopant ion clustering mapping.

### 3.5 Eu^3+^ emission in the studied samples

**FIGURE 6:**
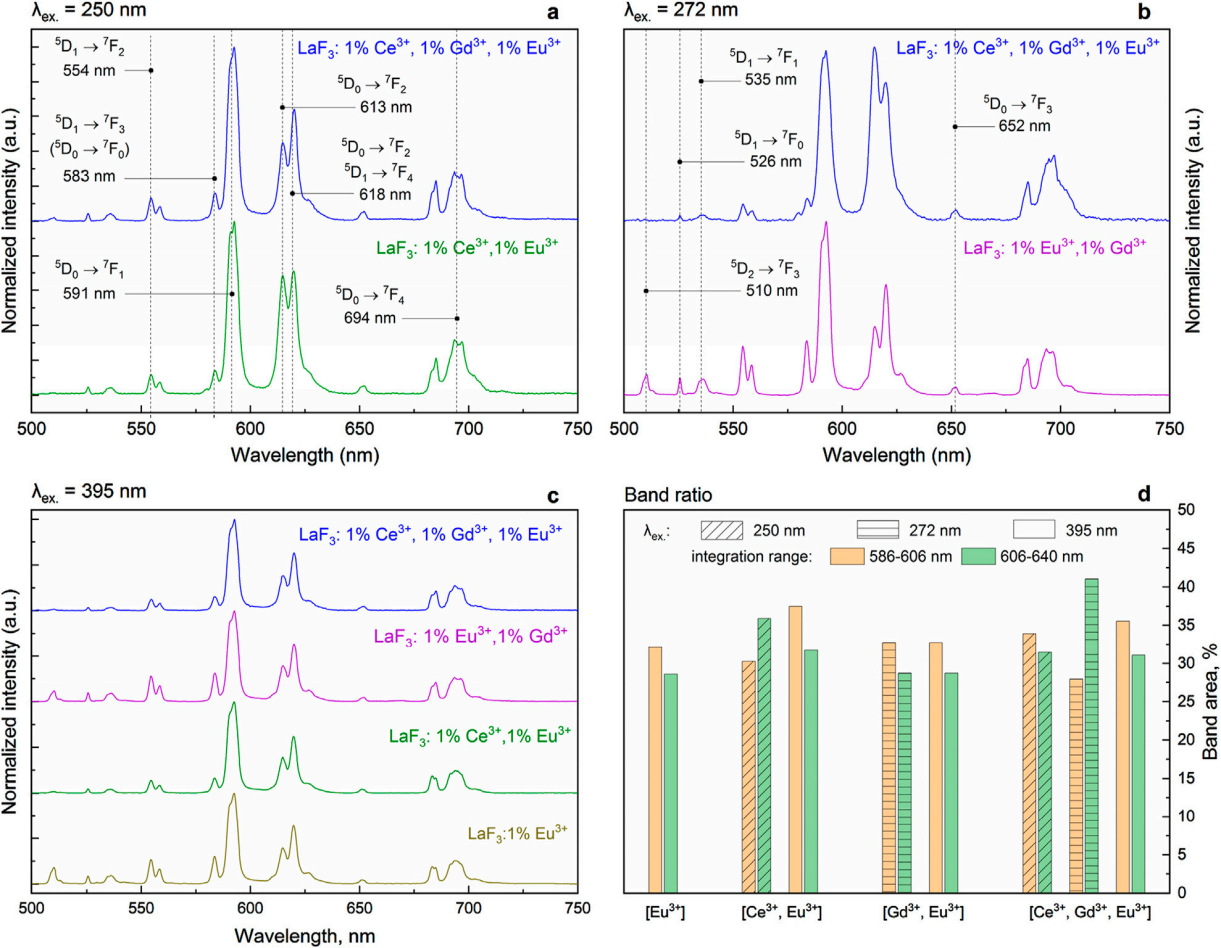
Excitation/sensitizer dependence of the Eu^3+^ emission of the studied samples. Panel d shows the ratios of selected band areas.

In the LaF_3_:Eu^3+^; LaF_3_:Ce^3+^,Eu^3+^; LaF_3_:Gd^3+^,Eu^3+^; and LaF_3_:Ce^3+^,Gd^3+^,Eu^3+^ samples, the Eu^3+^ dopant exhibits similar, albeit excitation-dependent, emission spectra. In particular, the 613 nm band (hypersensitive), the 618 nm band, and, to some extent, the 583 nm band exhibit different relative intensities when excited at either 250 nm or 272 nm excitation (Figure 6). In the case of 395 nm excitation, the variation is much smaller. In other words, emission is also dependent on the sensitization path. Noteworthy, the bands in the 500–550 nm range (^5^D_1_→^7^F_0_ at 534–536 nm, ^5^D_0_→^7^F_1_ at 525.7 nm, and ^5^D_2_→7F_3_ at 509–510 nm) are much less distinct if a Ce co-dopant is present (Figure 6c).

This property is shown in Figure 6d, which shows the integrated areas of the emission spectra ranges 586–606 nm and 606–640 nm, in % of the total integrated spectrum. The very presence of such a phenomenon indicates some inhomogeneities in the structure. At least two spectroscopically different (although similar) Eu^3+^ sites are present. The sites differ not only in the first coordination sphere (judging by the shapes of the spectra) but also in their Ln^3+^ nearest neighbors (judging by the dependence on the sensitization path).

It is obvious that the excitation dependence is most prominent in samples containing Ce^3+^. Because the ionic radius of Ce^3+^ (1.196 Å, c. n. 9) is similar to that of La^3+^ (1.216 Å, c. n. 9), doping of LaF_3_ with Ce^3+^ should not result in any significant defect formation. On the other hand, the core-shell structure of TbF_3_ and CeF_3_ (instead of the mixed-lanthanide system) may form spontaneously (Grzyb et al., 2013). It is thus plausible that the introduction of Ce^3+^ somehow changes the character of the formed dopant ion clusters, resulting in increased asymmetry of the coordination geometry of some Eu^3+^ ions.

### 3.6 Curve fitting for samples containing Eu^3+^

This paper presents four kinds of Eu-containing samples and six wavelengths at which the emission temporal evolution profiles were measured. There are thus 24 rise-and-decay profiles, and each of them can tell a different story. While some fits were unambiguous and simple, others required a lot of work to complete. Only the most noteworthy and illustrative cases of fitting peculiarities are discussed below, with plots where necessary. For clarity, in many cases, only lifetimes are shown below. The respective full data for the mentioned cases are provided in the supplementary tables (Supplementary Data Sheet S1).

The profiles were fitted with the function of the following kind:

$$\begin{array}{ll} I= & I_{0}+A_{11}\;\left(1\;–\;\exp\left(\;–\;t/\tau_{1r}\right)\right)\exp\left(–\;t/\tau_{1d}\right)\\ & +A_{12}\;\left(1\;–\;\exp\left(\;–\;t/\tau_{1r}\right)\right)\exp\left(–\;t/\tau_{2d}\right)\\ & +A_{21}\;\left(1\;–\;\exp\left(\;–\;t/\tau_{2r}\right)\right)\exp\left(–\;t/\tau_{1d}\right)\\ & +A_{22}\;\left(1\;–\;\exp\left(\;–\;t/\tau_{2r}\right)\right)\exp\left(–\;t/\tau_{2d}\right)\\ & +A_{31}\;\left(1\;–\;\exp\left(\;–\;t/\tau_{3r}\right)\right)\exp\left(–\;t/\tau_{1d}\right)\\ & +A_{32}\;\left(1\;–\;\exp\left(\;–\;t/\tau_{3r}\right)\right)\exp\left(–\;t/\tau_{2d}\right)\\ & +A_{13}\;\left(1\;–\;\exp\left(\;–\;t/\tau_{1r}\right)\right)\exp\left(–\;t/\tau_{3d}\right)\\ & +A_{23}\;\left(1\;–\;\exp\left(\;–\;t/\tau_{2r}\right)\right)\exp\left(–\;t/\tau_{3d}\right)\\ & +A_{33}\;\left(1\;–\;\exp\left(\;–\;t/\tau_{3r}\right)\right)\exp\left(–\;t/\tau_{3d}\right). \end{array}$$ (39)

This function was used for all samples containing the Eu^3+^ dopant. It thus contains a total of nine rise-and-decay components (r3d3), as that was the highest complexity required. For many samples, only some of the components were used. The general form of the function remained the same to facilitate comparison. See Supplementary Data Sheet S2, Section SI.1.3 for the full list of functions. In several cases, a function with one rise and four decay components was used (r1d4, Equations 40, *A*_5_ = 0):

$$I=I_{0}+A_{0}\;\left(1\;–\;\exp\left(\;–\;t/\tau_{r}\right)\right)\;\left(\exp\left(–\;t/\tau_{1d}\right)+A_{2}\exp\left(–\;t/\tau_{2d}\right)+A_{3}\exp\left(–\;t/\tau_{3d}\right)++A_{4}\exp\left(–\;t/\tau_{4d}\right)+A_{5}\exp\left(–\;t/\tau_{5d}\right)\;\right.).$$ (40)

The functions are shown in a slightly simplified form. The actual functions used also had time offset as a variational parameter, that is, (*t*−*t*_0_), instead of only *t* in Equations 39, 40. Equation 40 has five decay components and is thus the r1d5 function. With *A*_2_-*A*_5_ set to 0, the simplest pulse function (r1d1) is obtained. With *A*_3_-*A*_5_ set to zero, the function is r1d2. With A_3_–A_5_ set to 0, the function is r1d2. With *A*_*4*_ and *A*_5_ set to zero, the function is r1d3.

Exponential decay has a general form:

$$I=I_{0}+\Sigma_{i}\;\left(\;A_{i}\exp\left(–\;t/\tau_{i}\right)\;\right),i=1,2,3,.$$ (41)

#### 3.6.1 The 694 nm emission

The pulsed-excitation emission of the samples at 694 nm can solely be attributed to the ^5^D_0_→^7^F_4_ Eu^3+^ transition. Fits were stable and easily achievable. The case is shown here to illustrate the way the fits “match the trend.” Even though transitions other than ^5^D_0_→^7^F_4_ of Eu^3+^ do not contribute to this emission (due to distinctly different transition energies), different samples exhibited different values of rise-and-decay lifetimes and different numbers of components (Table 4). However, regardless of the differences, the lifetimes are grouped quite well because the values do not differ much. For instance, rise times of 20–60 μs and 2.8–7 ms and decay times of 0.3–0.6 ms and 12–15 ms are present in all of the fits. This is a rough but distinct trend. Having several fit results that exhibit similar trends, the others are expected to fall within it. If there were several similarly good fit results with different lifetimes, the trend was used to select the “correct” option.

**TABLE 4:** Lifetimes of the 694 nm emission of the studied samples under different pulsed excitation, in μs, from the fits using Equation 39. Missing values are zero.

Sample	λ_ex._, nm	*τ*_r1_, μs	*τ*_r2_, μs	*τ*_r3_, μs	*τ*_d1_, μs	*τ*_d2_, μs	*τ*_d3_, μs
LaF_3_:Eu^3+^	394		35.8	5,408	495	13,321	1,254
LaF_3_:Ce^3+^,Eu^3+^	250		41.2	2,787	450	12,327	5,520
LaF_3_:Ce^3+^,Eu^3+^	394	6.492	46.1	3,029	449	12,773	8,629
LaF_3_:Gd^3+^,Eu^3+^	272		41.9	7,954	635	14,673	1,703
LaF_3_:Gd^3+^,Eu^3+^	394	24.767	99.5	4,751	503	12,598	1,079
LaF_3_:Ce^3+^,Gd^3+^,Eu^3+^	250	0.619	39.6	6,972	297	14,740	3,800
LaF_3_:Ce^3+^,Gd^3+^,Eu^3+^	272		21.8	5,105	527	14,195	
LaF_3_:Ce^3+^,Gd^3+^,Eu^3+^	394		63.6	2,799	406	12,670	

#### 3.6.2 LaF_3_:Ce^3+^,Gd^3+^,Eu^3+^; λ_ex._ = 250 nm, λ_em._ = 591 nm

The fit of the profile of emission at 591 nm, with 250 nm excitation of the LaF_3_:Ce^3+^,Gd^3+^,Eu^3+^ sample, turned out to be extremely troublesome and required a noteworthy decision. The perfect flat residual can be obtained with Equation 40 using five decay components. The fit resulted in a rise time of almost 16 ms (Table 5), which is approximately three times longer than expected from the appearance of the curve (maximum at approximately 5 ms).

**TABLE 5:** Lifetimes for selected fit results LaF_3_:Ce^3+^,Gd^3+^,Eu^3+^ emission temporal evolution (λ_ex._ = 250 nm, λ_em._ = 591 nm). Missing values are zero. Values are grouped by similarities.

Equation 41* (*i* = 1,2)		*τ*_1_, μs					*τ*_2_, μs
		4,804					14,688
Equation 40*		*τ*_r_, μs	*τ*_d1_, μs	*τ*_d2_, μs	*τ*_d3_, μs	*τ*_d4_, μs	*τ*_d5_, μs
		5,638					14,848
Equation 40		15,754	44	365	1,511	5,053	13,344
Equation 39	*τ*_r1_, μs	*τ*_r3_, μs			*τ*_d1_, μs	*τ*_d2_, μs	*τ*_d3_, μs
	25	4,983			333	1,756	15,284

*Late part fits, 20–50 ms range.

Moreover, the part after 20 ms can be fitted with Equation 40 with only one decay component, resulting in a rise time of 5.6 ms. Note that that part does not have any apparent rise. A similar result is obtained with two independent exponential decays (Equation 41), one of which converges with a negative amplitude, indicating a rise of 4.8 ms.

**FIGURE 7:**
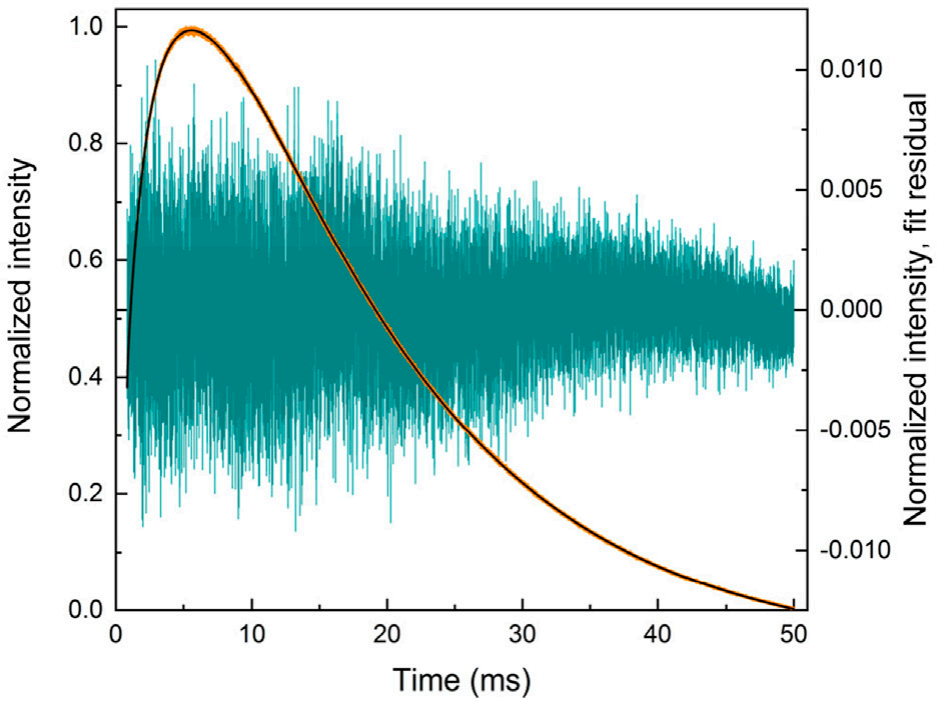
Temporal evolution of LaF_3_:Ce^3+^,Gd^3+^,Eu^3+^ emission (λ_ex._ = 250 nm, λ_em._ = 591 nm), the fit, and the residual. Notice the tail of the residual curving down at 40–50 ms.

An alternative fit was obtained with a slight curvature of the residual in the 40–50 ms region (see Figure 7), which was, however, still smaller than the noise. Consecutive refits (with a simpler function Equation 16 type, convoluted) with an increasing number of data points were barely stable, performing hundreds of function evaluations without any significant parameter changes. With the Equation 40 function (non-convoluted), a stable solution was finally found. See Tables 5, 6 for the details. This second fit matches the trend in the values of rise-and-decay times (see the supplementary tables (Supplementary Data Sheet S1)) and is actually what it should be judging by the appearance of the curve. The case described illustrates one of the principles of good fitting: it is important to select fits that make sense and correspond to other fits, even at the cost of slightly lower quality. However, there must be a reasonable limit to this “lower.”

**TABLE 6:** Coefficients for selected fit results LaF_3_:Ce^3+^,Gd^3+^,Eu^3+^ emission temporal evolution (λ_ex._ = 250 nm, λ_em._ = 591 nm). Missing values are zero.

Equation 41* (*i* = 1,2)	*A*_1_					*A*_2_
	−1.558					2.267
Equation 40*	*A*_0_		*A*_2_	*A*_3_	A_4_	A_5_
	2.134					
Equation 40	4.798		2.882	1.596	0.876	0.623
Equation 39	*A*_11_	*A*_12_	*A*_13_	*A*_31_	*A*_32_	*A*_33_
	0.044	0.003	0.391	0.800	1.303	1.624

*Late part fits, 20–50 ms range.

#### 3.6.3 LaF_3_:Ce^3+^,Eu^3+^, λ_ex._ = 250 nm, λ_em._ = 591 nm

**FIGURE 8:**
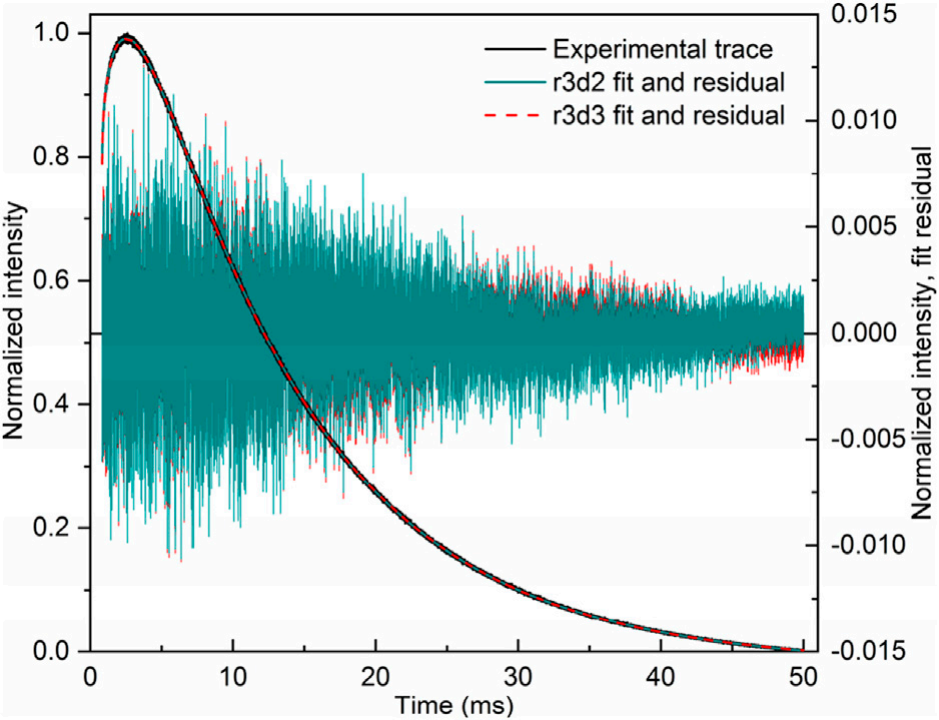
Temporal evolution of LaF_3_:Ce^3+^,Eu^3+^ emission (λ_ex._ = 250 nm, λ_em._ = 591 nm), the fits, and the residuals. Notice the tail of the residual at 40–50 ms: one of the data sets curves slightly upward, while the other does not.

Figure 8 presents the decay profile of the 591 nm emission under 250 nm excitation of the LaF_3_:Ce^3+^,Eu^3+^ sample. At the later part of the decay, in the 5–50 ms part, there was still some rise present. The decay-only pattern is apparent only after 10 ms. The 10–50 ms part can be fitted with a monoexponential decay with a lifetime of approximately 12 ms. Alternatively, biexponential decay can be obtained with the same quality, with lifetimes of 11.5 ms and 14.3 ms.

The residuals of the fits are very similar, and both are not essentially flat. For the 12 ms solution, the residual curves upward slightly in the 40–50 ms part, indicating a probable presence of another (much longer) decay component. In the case of the second solution, this curvature does not occur (almost identically to the rightmost part of the plots in Figure 8). The conclusion is ambiguous: two sites with lifetimes of 11.5 ms and 14.3 ms can be assumed, although, at the same time, this result may simply be an overfit. According to the principle of Occam’s razor, this case should be interpreted as a noisy monoexponential decay rather than a biexponential decay with similar lifetimes. Such a conclusion cannot be safely made for the whole range of rise-and-decay fit.In this example, interesting behavior was observed for the 15–50 ms range (values in *italics* in Table 7). A stable fit with a flat residual can be obtained with decay times of 11.6 ms and 28.8 ms. Now, take the following procedure: construct a guess using the coefficients from the previous result and lifetimes of 11.6 ms and 13 ms; the fit will converge with several new coefficients and lifetimes of 11.6 ms and 16–28 ms. Repetition of this procedure results in many values of the minor lifetime between 16 ms and 28 ms, all of which are guess-dependent. The fit as a whole is also range dependent; see Table 7. This example clearly illustrates that **cropping out the rise** (or, in general, cropping some part of the decay profile) **is a foul practice** that can lead to false data, erroneous data, moderately incorrect data, or data manipulation, depending on the specific case and the intention of the experimenter.

**TABLE 7:** Exponential decay fits (Equation 41, i = 1, i = 1,2) of the decay-only part of the LaF_3_:Ce^3+^,Eu^3+^ emission profile (λ_ex._ = 250 nm; λ_em._ = 591 nm).

Range, ms	*A*_1_	*τ*_d1_, μs	*A*_2_	*τ*_d2_, μs	*τ*_d-av_, μs
20–50	1.458	12,117			
15–50	1.476	12,029			
10–50	1.478	12,017			
20–50	1.239	11,110	0.2700	16,085	12,000
20–50	1.464	11,737	0.0341	34,791	12,262
15–50	1.478	11,710	0.0331	53,686	12,631
15–50	1.458	11,600	0.0513	*28,793*	12,184
15–50	1.419	11,465	0.0911	*21,575*	12,075
15–50	1.388	11,380	0.1226	*19,574*	12,045
15–50	1.317	11,215	0.1958	*17,340*	12,008
15–50	1.284	11,151	0.2285	*16,758*	11,998
15–50	1.280	12,590	0.2507	7,902*	11,822
10–50	1.215	11,504	0.2716	14,348	12,024
10–50	1.472	11,891	0.0161	42,477	12,223

* Moderately stable fit.

$\tau_{d-\text{av}}=\left(A_{1}\tau_{d1}+A_{2}\tau_{d2}\right)/\left(A_{1}+A_{2}\right)$

Good full-range fits can be achieved with either r3d2 (Fit 1, Fit 2, Table 8) or r3d3 (Fit 3, Table 8) kinetics. Fit 3 and Fit 1 result in a flat residual, while Fit 2 does not. Depending on the guess, the decay lifetimes may be: 9.8 ms, 13.6 ms; 1.2 ms, 12 ms; or 1.1 ms, 11.7 ms, 39.8 ms. In other words, the solutions are still inconclusive: Fit 1 falls off the trend with its 9.8 ms decay component, Fit 2 shows a small signal in the residual, while Fit 3 shows a questionable decay component of 39.8 ms lifetime and an unknown nature (although its amplitude is rather low). As a result, the Fit 2 solution was accepted.

**TABLE 8:** Lifetimes of the LaF_3_:Ce^3+^,Eu^3+^ emission profile (λ_ex._ = 250, 394 nm; λ_em._ = 591 nm), from Equation 39 fits.

λ_ex._, nm		*τ*_r1_, μs	*τ*_r2_, μs	*τ*_r3_, μs	*τ*_d1_, μs	*τ*_d2_, μs	*τ*_d3_, μs	Residual
250	Fit 1	44.2	304	2,480	9,760	13,604		flat
250	Fit 2	40.2	146	1,722	1,180	11,981		a bit curved
250	Fit 3	41.2	198	2,065	1,115	11,667	39,823	flat
394		28.8	107	2,543	1909	12,152		

#### 3.6.4 LaF_3_:Ce^3+^,Eu^3+^, λ_ex._ = 394 nm, λ_em._ = 591 nm

Similarly, the 15–50 ms fragment of the temporal evolution profile of the 591 nm emission (excited at 394 nm) of the LaF_3_:Ce^3+^,Eu^3+^ sample can be fitted with either monoexponential decay (12 ms) or biexponential decay. Attempts to add a second decay component to the former solution result in lifetimes of 11 ms and 13 ms. If the result is used as a guess in a new fit, but the 13 ms lifetime is replaced with 20 ms, the fit converges with lifetimes of 12 ms and 15 ms. Repeating this procedure with a gradually increasing second lifetime eventually results in a stable solution with lifetimes of 12 ms and 37 ms, the latter having a negligible amplitude. All solutions are characterized with flat residuals, and one must be chosen based on them. The whole-range r3d2 fit shows two decay lifetimes of 2 ms and 12 ms (Table 8). The conclusion from Section 3.6.3 is also reproduced here: **it is unsafe to simply crop out the rise part** and hope for the best with the decay part fit.

#### 3.6.5 LaF_3_:Ce^3+^,Eu^3+^, λ_ex._ = 250 nm, λ_em._ = 583 nm

The temporal evolution of the 583 nm emission of the LaF_3_:Ce^3+^,Eu^3+^ sample after 250 nm pulsed excitation can be fitted using the r2d3 and r2d4 functions, with the same (good) residuals. The r2d3 solution exhibits the decay lifetimes corresponding to those from tail-only fit. Another argument toward the r2d3 result is overfitting avoidance: choose the simpler of two similarly good solutions.

**FIGURE 9:**
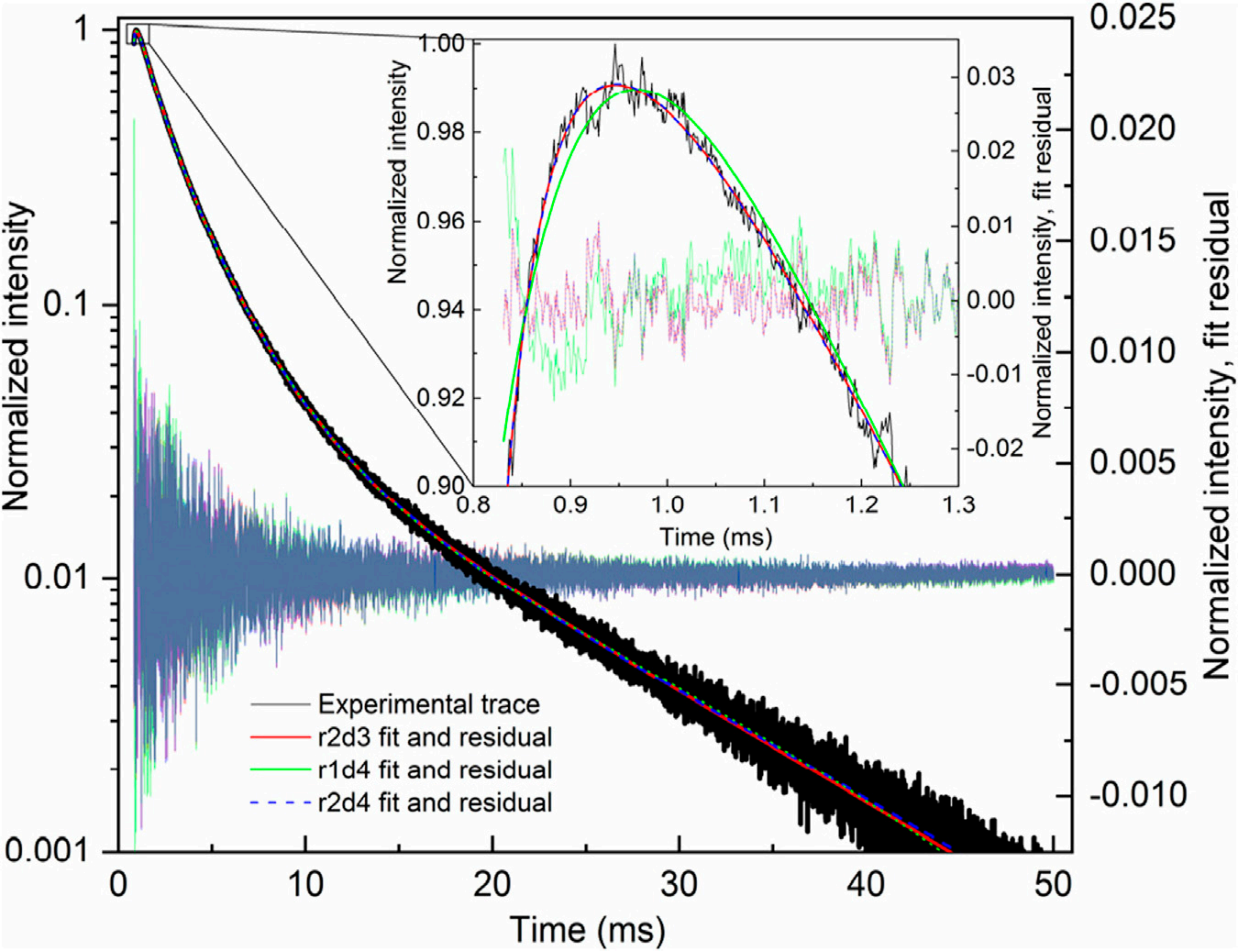
Temporal evolution of LaF_3_:Ce^3+^,Eu^3+^ emission (λ_ex_ = 250 nm, λ_em_ = 583 nm), the fits, and the residuals (semi-transparent, overlapping). The inset shows an enlarged plot of the top of the curve.

From Figure 9, it is clear that the r2d3 and r2d4 solutions mostly overlap and also perfectly match the experimental trace. The r1d4 solution has a noticeably incorrect shape at the top of the curve. The discrepancy is also visible in the residuals. However, the differences are quite subtle and barely noticeable in the whole-range plot.

#### 3.6.6 LaF_3_:Ce^3+^,Eu^3+^; λ_ex_ = 394 nm, λ_em_ = 583 nm

**FIGURE 10:**
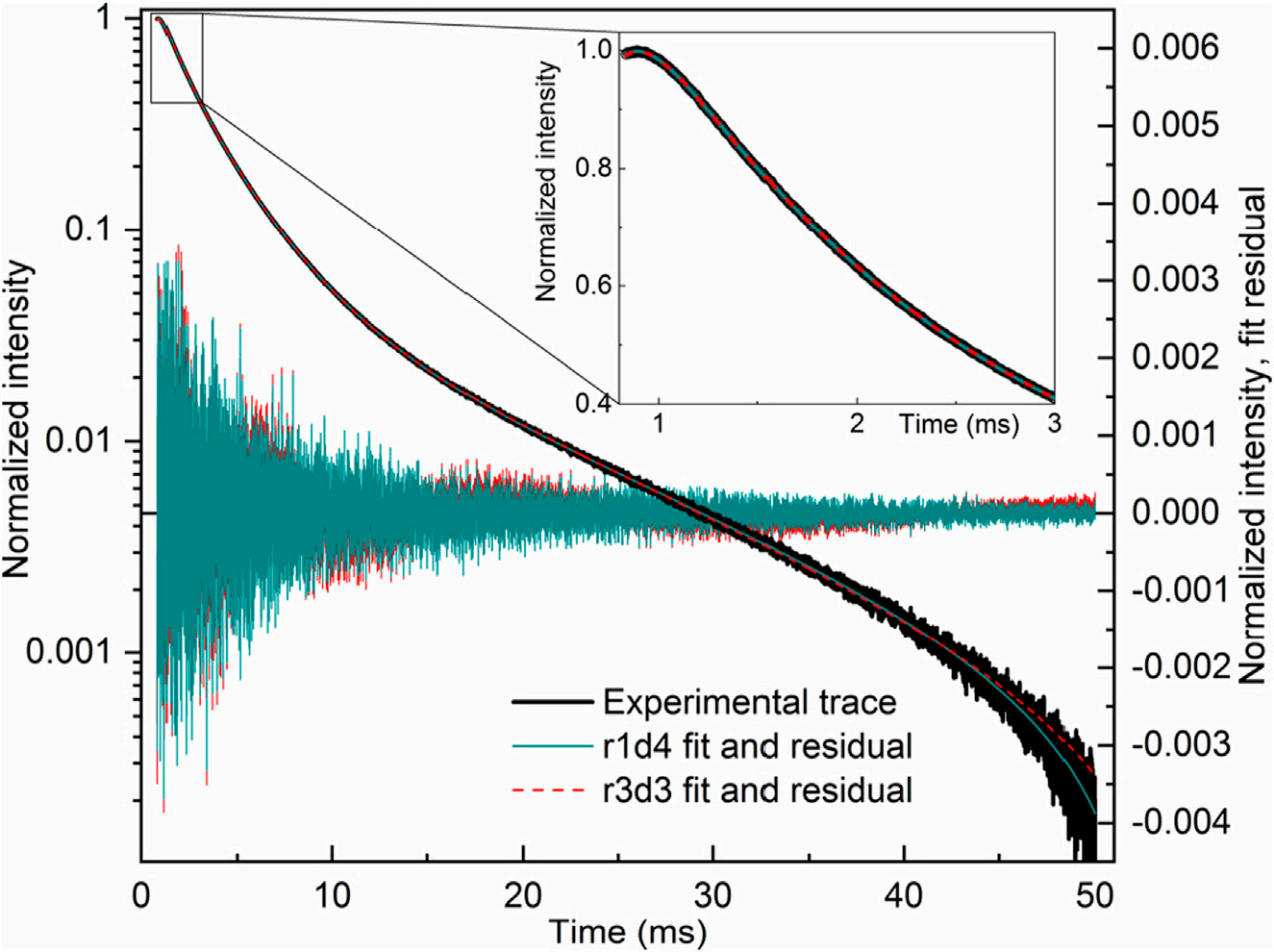
Temporal evolution of LaF_3_:Ce^3+^,Eu^3+^ emission (λ_ex._ = 394 nm, λ_em._ = 583 nm), the fits, and the residuals. Note the red/r3d3 residual oscillating up and down relative to the green one. The inset shows an enlarged plot of the top of the curve.

The temporal evolution of the 583 nm emission of the LaF_3_:Ce^3+^,Eu^3+^ sample after 394 nm pulsed excitation is characterized by a very short rise at the very top. A fit with r1d3 kinetics resulted in lifetimes more similar to those of LaF_3_:Ce^3+^,Eu^3+^, λ_ex._ = 250 nm, λ_em._ = 583 nm decay profile (Table 9), but the corresponding residual pattern was curved (similar to the r3d3 one in Figure 10). Consequently, the r1d4 fit was accepted. It is worth paying attention to the oscillating residual: it may indicate both underfitting (too few components) or overfitting (too many components, for example, r3d3).

**TABLE 9:** Lifetimes of the LaF_3_:Ce^3+^,Eu^3+^ emission profile (λ_ex._ = 250 nm, 394 nm, λ_em._ = 583 nm), from Equation 39 and Equation 40 fits.

λ_ex._, nm		*τ*_r1_, μs	*τ*_r2_, μs	*τ*_r3_, μs	*τ*_d1_, μs	*τ*_d2_, μs	*Τ*_d3_, μs	*τ*_d4_, μs
250	r2d3/Equation 39	36.7	188			1,088	2,612	10,772
250	r1d4/Equation 40		113		1,264	1,262	2,883	12,097
250	r2d4/Equation 39	40.5	219		797	1,517	2,840	11,066
394	r1d3/Equation 40		182			1,156	2,729	10,886
394	r1d4/Equation 40		194		856	1,724	3,175	12,011

#### 3.6.7 LaF_3_:Ce^3+^,Eu^3+^, λ_ex._ = 394 nm, λ_em._ = 613 nm

The 613 nm emission of the LaF_3_:Ce^3+^,Eu^3+^ sample after 394 nm pulsed excitation presents a peculiar case with similar rise-and-decay lifetimes. An intermediate solution was obtained with a rise lifetime of approximately 7 ms and a decay time of approximately 5.5 ms (Fit 1, Table 10). Alternatively, a rise time of 5.3 ms and a decay time of 6 ms can be obtained. In both cases, there is a longer decay component of approximately 12 ms. Finally, the full Equation 39 was fitted to the profile, resulting in rise lifetimes of 20.1 μs, 118 μs, and 5,269 μs, and decay lifetimes of 599 μs, 5,986 μs, and 12,431 μs (Fit 2, Table 10). Note the values in bold in Table 10: rise-and-decay components with similar lifetimes (especially with the rise time greater than the decay time) might partially cancel out, resulting in parameter dependence and instabilities in the fit procedure. It is also the probable reason for such a large difference in rise times, with other parameters almost identical.

**TABLE 10:** Lifetimes of the LaF_3_:Ce^3+^,Eu^3+^ emission profile (λ_ex._ = 394, λ_em._ = 613 nm), from Equation 39 fit.

Function	Range, ms	*A*_1_	*τ*_d1_, μs	*A*_2_	*τ*_d2_, μs	*τ*_d-av_, μs
Equation 41, *i* = 1	30–50	0.828	12,158			
Equation 41, *i* = 1,2	10–50	0.270	8,566	0.638	12,936	11,635
	15–50	0.271	8,536	0.638	12,942	11,630
	15–50	0.689	10,553	0.210	15,811	11,783
	20–50	0.684	10,633	0.210	15,608	11,803
	15–50	0.195	7,673	0.719	12,625	11,567
	15–50	0.170	6,197	0.777	12,405	11,288
	20–50	0.157	6,186	0.782	12,382	11,345
	25–50	0.258	4,685	0.807	12,273	10,432

Function	Name	Range, ms	*τ*_r1_, μs	*τ*_r2_, μs	*τ*_r3_, μs	*τ*_d1_, μs	*τ*_d2_, μs	*τ*_d3_, μs
Equation 39	Fit 1	0.8–50	20.0	119	**7,088**	596	**5,542**	12,403
Equation 39	Fit 2	0.8–50	20.1	118	**5,269**	599	**5,986**	12,431

$\tau_{d-\text{av}}=\left(A_{1}\tau_{d1}+A_{2}\tau_{d2}\right)/\left(A_{1}+A_{2}\right)$

Partial (decay-only) fits also show range-dependence and guess-dependence in this case. Some partial fits correspond well to the full fits (underlined values), but others do not (Table 10).

#### 3.6.8 LaF_3_:Ce^3+^,Eu^3+^, λ_ex._ = 250 nm, λ_em._ = 613, 618 nm

The case of the 613 nm and 618 nm emission of the LaF_3_:Ce^3+^,Eu^3+^ sample after pulsed excitation at 250 nm was very interesting. First, two transitions with apparently similar wavelengths show different kinetics: most importantly, the relatively long rise is not visible in the 613 nm profile. Accurate analysis based on the Carnall tables indicates that the 613 nm emission most likely corresponds exclusively to the ^5^D_0_→^7^F_2_ transition, while the 618 nm emission is a mix of ^5^D_0_→^7^F_2_ and ^5^D_1_→^7^F_4_ transitions. This will be discussed in more detail below.

Fitting of the 618 nm profile proved troublesome. On the one hand, the fits of the later part of the profile (starting at 10 ms, 15 ms, and 20 ms) were ambiguous (Table 11), sometimes unstable, and guess-dependent. Yet, several solutions of the same quality (i.e., same R^2^, same residual, stable) with significantly different lifetimes were obtained (bold values, Table 11).

**TABLE 11:** Lifetimes of the LaF_3_:Ce^3+^,Eu^3+^ emission profile under 250 nm excitation.

λ_em,_ nm	Name	Range, ms	Function	*τ*_r1_, μs	*τ*_r2_, μs	*τ*_d1_, μs	*τ*_d2_, μs	*τ*_d3_, μs	*τ*_d4_, μs
613		10–50	d1+d1, Equation 41					4,376	12,146
613	Fit 1	0.8–50	r1d4, Equation 40	33.2		535	**1,437**	4,759	12,256
618	Fit 2	0.8–50	r2d3, Equation 39	45.2	**2,520**	471		6,855	12,619
618	Fit 3	0.8–50	r2d3, Equation 39	47.8	**6,702**	452		4,553	12,258
618		15–50	d1, Equation 41						11,889
618		20–50	d1, Equation 41						12,046
618		20–50	r1d1, Equation 40		**1,656**				12,046
618		10–50	r1d2, Equation 40		**1,398**			8,428	13,340
618		10–50	r1d2, Equation 40		**3,608**			3,173	12,140

Given the mix of transitions, a cross-relaxation process can be assumed: ^5^D_0_→^7^F_2_ ··· ^7^F_4_ → ^5^D_1_. Therefore, there must be a decay component in the 613 nm profile with a lifetime similar to one of the rise lifetimes in the 618 nm profile; a likely candidate is the 1.4 ms decay component of Fit 1 (Table 11). In this case, the consistency condition requires Fit 2 to be accepted based on the similarity between the 1.4 ms decay of Fit 1 and the 2.5 ms component of Fit 2. However, the same condition requires selecting Fit 3 on the basis of the similarity of the 4.5–4.8 ms decay components and a nearly perfect match in the 12 ms decay component (underlined values). None of the choices are supported by partial fits.

It is crucial to emphasize that there are no numerical differences between the fits: R^2^ values are the same, the residual pattern is the same (patternless noise), and the reproducibility is the same. The choice of either option was entirely up to the researcher.

Because the excitation source in both cases is an energy transfer from Ce^3+^ (taking into account its efficient absorption at 250 nm), Fit 3 is more likely to be the correct option. The Eu-Eu cross-relaxation thus has a minor effect (which is reasonable, given that both transitions of Eu^3+^ are forbidden, while the Ce^3+^ one is allowed). It is possible that the similarity of the order of magnitude of the 1.4 ms decay in Fit 1 and the 2.5 ms rise in Fit 2 is coincidental.

#### 3.6.9 LaF_3_:Ce^3+^,Gd^3+^,Eu^3+^, λ_ex._ = 272 nm, λ_em._ = 613 nm

**FIGURE 11:**
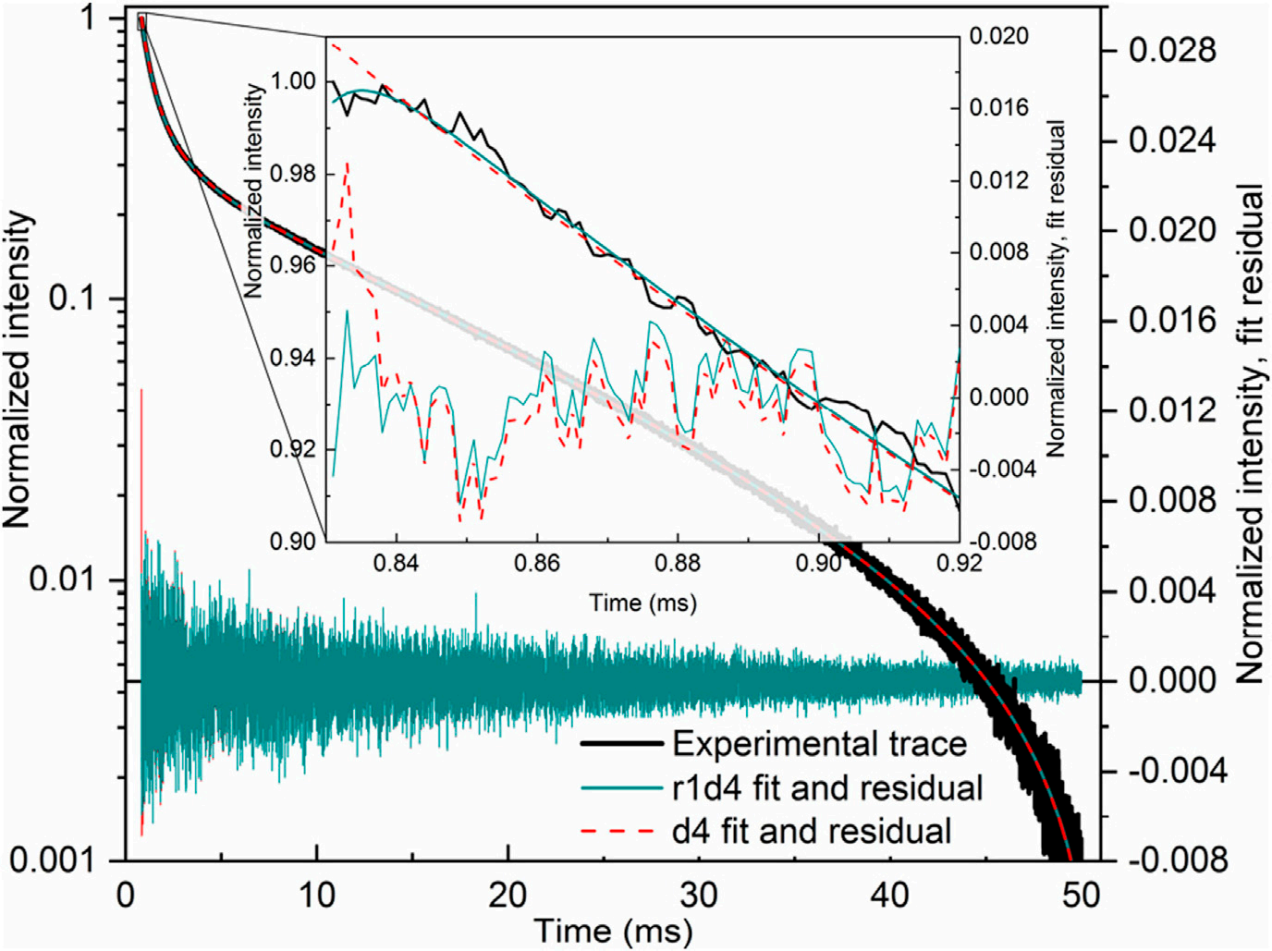
Temporal evolution of LaF_3_:Ce^3+^,Gd^3+^,Eu^3+^ emission (λ_ex._ = 272 nm, λ_em._ = 613 nm), fits, and residuals (semi-transparent, overlapping). The inset shows an enlarged plot of the top of the curve. The fits and residuals only differ in the 0.82–0.87 ms range.

In the 613 nm emission of the LaF_3_:Ce^3+^,Gd^3+^,Eu^3+^ sample with 272 nm excitation, distinguishing between r1d4 and simply d4 kinetics was based on only a few points at the beginning of the profile. However, the difference was clear enough to keep the rise in the model (Figure 11).

### 3.7 The LaF_3_:Eu^3+^ sample

The fitting results for the temporal evolution profiles of the LaF_3_:Eu^3+^ emission are shown in Tables 12, 13. To improve the readability of the tables, the coefficients have been rounded to three decimals and the lifetimes to at least three digits. Tables with more significant digits are shown in the supplementary tables (Supplementary Data Sheet S1).

**TABLE 12:** The values of *A* (amplitudes, Equation 39) for the LaF_3_:Eu^3+^ sample, λ_ex._ = 394 nm. Bold values are specifically referred to in the text.

λ_em_., nm	Transition	*A*_21_	*A*_31_	*A*_12_	*A*_22_	*A*_32_	*A*_13_	*A*_23_	*A*_33_
552	^5^D_1_→^7^F_2_			0.698	0.364	0.354			
583	^5^D_1_→^7^F_3_ ^5^D_0_→^7^F_0_ (?)			0.697	0.024	0.312	0.017	0.149	0.017
591	^5^D_0_→^7^F_1_	**0.310**	0.001		0.356	**1.976**		1.554	1.314
613	^5^D_0_→^7^F_2_	**0.310**	0.095		**0.475**	0.164		0.047	0.247
618	^5^D_0_→^7^F_2_, ^5^D_1_→^7^F_4_	**0.161**	0.035		**0.598**	0.032		0.026	0.559
694	^5^D_0_→^7^F_4_	**0.248**	0.066		0.365	**0.375**		0.100	0.550

**TABLE 13:** The values of *τ* (lifetimes, Equation 39) for the LaF_3_:Eu^3+^ sample, λ_ex._ = 394 nm.

λ_em_., nm	Transition	*τ*_1r_, μs	*τ*_2r_, μs	*τ*_3r_, μs	*τ*_1r_, μs	*τ*_2r_, μs	*τ*_3r_, μs
552	^5^D_1_→^7^F_2_	123	634	1,668		4,443	
583	^5^D_1_→^7^F_3_	129	814	2,579		4,355	12,313
591	^5^D_0_→^7^F_1_		44.9	4,684	421	1,272	13,450
613	^5^D_0_→^7^F_2_		48.2	5,269	507	1,097	13,216
618	^5^D_0_→^7^F_2_, ^5^D_1_→^7^F_4_		57.9	5,493	565	7,469	13,396
694	^5^D_0_→^7^F_4_		35.8	5,408	495	1,254	13,321

Transition manifolds were identified using the Carnall tables. The 618 nm curve has noticeably different lifetimes than other transitions from the ^5^D_0_ manifold. According to the tables, for the ^5^D_0_→^7^F_2_, transition, the wavelength is 614.7 nm, while the wavelength of the ^5^D_1_→^7^F_4_ transition is 617.1 nm. The corresponding *A*_32_ coefficient of the 618 nm fit (Table 12) is much lower than for the 591 nm, 613 nm, and 694 nm traces, which indicates a small participation of the rise-3-decay-2 component (5.5 ms rise time, 7.5 ms decay time). Therefore, it was concluded that there is an admixture of ^5^D_1_→^7^F_4_ emission in the dynamics of 618 nm photoluminescence. The resulting decay-2 component falls off the trend with its value of approximately 7.5 ms, while the remaining three are approximately 1 ms.

The 552 nm emission unambiguously corresponds to ^5^D_1_→^7^F_2_ of Eu^3+^. The 583 nm emission can be assigned to ^5^D_0_→^7^F_0_. However, Carnall’s tables show that the latter must be located at 578.3 nm. The energy of the ^5^D_1_→^7^F_3_ transition corresponds to 582.7 nm, while the dynamics of the 552 and 583 nm emission are quite similar, except for the decay-3 component of the latter. The coefficients involving decay-3 are rather small. Thus, the 583 nm emission is mainly ^5^D_1_→^7^F_3_, with some addition of ^5^D_0_ radiative decay. This decay may be due to the optical overlap of ^5^D_0_→^7^F_0_ emission from an independent site but may also originate from energy transfer interactions of a pair of neighboring Eu^3+^ ions.

One can speculate that the average lifetime of ^5^D_0_ is approximately 13 ms, while the average lifetime of ^5^D_1_ is approximately 1–5 ms. However, due to the complexity of the observed rise-and-decay kinetics and the fact that dopant-dopant interactions were observed in the LaF_3_:Gd^3+^ and LaF_3_:Ce^3+^,Gd^3+^ samples, there must be several interacting Eu^3+^ species in the studied system. The observed lifetimes may, therefore, result from the system of the interacting levels as a whole and not correspond to the actual radiative rates (*W*_*rad.*_) or total decay rates (*W*_*rad.*_ *+ W*_*nrad.*_) at the levels.

The Ce and Gd samples mentioned above could be treated using relatively simple model systems and empirical/fittable parameters. In contrast, the Eu^3+^ dopant comprised dozens of manifolds below the used excitation energies (corresponding to 272 nm for Gd-doped samples and 250 nm for Ce-doped samples) (Peijzel et al., 2005). Even a system of a single Eu^3+^ ion is a challenge, while a model system with two Eu^3+^ ions would contain many more degrees of freedom (approximately half a hundred) than can be reliably inferred from the experimental kinetics in question (in particular, six amplitudes and five lifetimes). To properly describe such a system, it is necessary to find transition dipoles for the transitions in question, calculate energy transfer rates, and estimate non-radiative transfer rates, as, for example, in Shyichuk et al. (2016b). In this paper, we thus leave the Eu^3+^ data without deeper analysis and keep the focus on temporal profile fitting.

## 4 Conclusion

In this paper, luminescence rise-and-decay profiles of a series of exemplary lanthanum fluoride (LaF_3_) systems doped with Ce^3+^, Gd^3+^, and Eu^3+^ ions were analyzed using pulsed laser excitation. We have shown that profiles of the temporal evolution of photoemission can provide many detailed insights into the underlying physics and chemistry—under the condition that they are analyzed and, more importantly, interpreted with the appropriate degree of detail. Building sets of rate equations and trying to reproduce experimental kinetics with them is crucial to achieving a clear picture of the luminescence mechanism, even in the case of seemingly simple systems such as LaF_3_:Gd^3+^.

The naive one-exponent-one-site interpretation of the lifetimes may turn out to be only partially correct, especially in cases where we are dealing with (multi-step) energy transfer. When many levels (more than three) interact in complex ways, “emergent” lifetimes may show up—that is, lifetimes that do not directly correspond to any specific rate of any particular process but result from the system of rate equations as a whole.

Finally, comprehensive interpretation relies on properly analyzed data. In this case, high-quality multiexponential fits are required to describe complex kinetics and provide a basis for further conclusions. It was shown that temporal profiles that feature a rise should not be simplified by chopping off the rise part, as such a simplification results in artifacts and data loss. The interplay between rise components and decay components is important and might strongly affect the interpretation of the experimental data. A rise-and-decay fit can provide much more information than a single rise lifetime and a single decay time that were found independently.

While the paper focuses on a specific material and a specific set of dopant ions, the conclusions regarding methodology are transferable to other materials and activators. The approaches described in this paper apply to any rise-and-decay kinetics, no matter the particular physical-chemical origin.

## Data Availability

The datasets are available in Supplementary Data Sheet S3.
