# Supplementary material for: LaF3 doped with Ce/Gd/Eu: energy transfer and excitation dependence of photoluminescence rise-and-decay kinetics
Source: Front Chem. 2025 Apr 22;13:1501039. doi: 10.3389/fchem.2025.1501039 (PMC12052759; doi:10.3389/fchem.2025.1501039)
Supplement: Supplementary file 1 [file DataSheet2.pdf]

LaF$_3$ doped with Ce/Gd/Eu: energy transfer and excitation dependence of photoluminescence rise-and-decay kinetics

Andrii Shyichuk[1], Daria Szeremeta[2], Marcin Runowski[2], Eugeniusz Zych[1,3], Stefan Lis[2]

[1] Faculty of Chemistry, University of Wroclaw, 14 F. Joliot-Curie, 50-383 Wrocław, Poland
[2] Faculty of Chemistry, Adam Mickiewicz University in Poznań, 8 Uniwersytetu Poznańskiego, 61-614 Poznań, Poland
[3] Helmholtz-Zentrum Dresden-Rossendorf, Bautzner Landstrasse 40001328 Dresden, Germany

Supplementary Information

SI.1. Rise-and-decay (pulse) functions

SI.1.1. A pulse function with initial population parameter

The simplest pulse function is described by the following equation, with rates instead of inverse lifetimes:

$$I(t) = A \exp\left(-t\, W_{\mathrm{nrad.}}\right) \left(1 - \exp\left(-2\, t\, W_{\mathrm{ET}}\right)\right) \quad \text{(S1)}$$

On certain occasions, a slightly different form of the pulse function is used, namely:

$$\begin{gathered} I = \exp\left(-t\, W_{\mathrm{rise}}\right) \left(A_1\left(1 - \exp\left(-t\, W_{\mathrm{decay}}\right)\right) + A_2 \right) = \\ A_2 \exp\left(-t\, W_{\mathrm{rise}}\right) + A_1 \exp\left(-t\, W_{\mathrm{rise}}\right) \left(1 - \exp\left(-t\, W_{\mathrm{decay}}\right)\right) \end{gathered} \quad \text{(S2)}$$

In such a function, another parameter A_2 is added, corresponding to the initial (pre-excitation) population of the emitting level. Opening the brackets clearly shows that this kind of function is a sum of a pulse and an exponential decay. For us, it seemed redundant. If the section of the experimental curve being fitted does not correspond perfectly to the time before the excitation pulse, the population of the emitting level might indeed be non-zero. However, in the pulse function fitting (Eq. S1, Eq. 11 in the main text), a temporal offset will compensate for that. Alternatively, an independent exponential decay component may be added to the solution if needed.

SI.1.2. List of multi-component rise-and-decay functions

The functions are named by the number of rise and decay components, in a rXdY fashion, where X is the number of rise components and Y is the number of decay components. Note that in the supplementary spreadsheet the variables are sometimes renamed. E.g. if a τ_{2d} value of a certain r2d2 solution is more similar to τ_{3d} from some other solutions, it will be relabeled to τ_{3d}, and the function will contain the following variables: A_{11}, A_{13}, A_{21}, A_{23}, τ_{1r}, τ_{2r}, τ_{1d}, τ_{3d}. The function is still r2d2 as it contains 2 rise components and 2 decay components.

$$I = I_0 + A_{11}(1 - \exp(-t/\tau_{1r}))\exp(-t/\tau_{1d}) \qquad \text{r1d1}$$

$$I = I_0 + A_{11}(1 - \exp(-t/\tau_{1r}))\exp(-t/\tau_{1d}) + A_{12}(1 - \exp(-t/\tau_{1r}))\exp(-t/\tau_{2d}) \qquad \text{r1d2}$$

$$\begin{aligned} I = I_0 + {} & A_{11}(1 - \exp(-t/\tau_{1r}))\exp(-t/\tau_{1d}) + A_{12}(1 - \exp(-t/\tau_{1r}))\exp(-t/\tau_{2d}) + \\ & A_{13}(1 - \exp(-t/\tau_{1r}))\exp(-t/\tau_{3d}) \end{aligned} \qquad \text{r1d3}$$

$$\begin{aligned} I = I_0 + {} & A_{11}(1 - \exp(-t/\tau_{1r}))\exp(-t/\tau_{1d}) + A_{12}(1 - \exp(-t/\tau_{1r}))\exp(-t/\tau_{2d}) + \\ & A_{13}(1 - \exp(-t/\tau_{1r}))\exp(-t/\tau_{3d}) + A_{14}(1 - \exp(-t/\tau_{1r}))\exp(-t/\tau_{4d}) \end{aligned} \qquad \text{r1d4}$$

$$\begin{aligned} I = I_0 + {} & A_{11}(1 - \exp(-t/\tau_{1r}))\exp(-t/\tau_{1d}) + \\ & A_{21}(1 - \exp(-t/\tau_{2r}))\exp(-t/\tau_{1d}) \end{aligned} \qquad \text{r2d1}$$

$$\begin{aligned} I = I_0 + {} & A_{11}(1 - \exp(-t/\tau_{1r}))\exp(-t/\tau_{1d}) + A_{12}(1 - \exp(-t/\tau_{1r}))\exp(-t/\tau_{2d}) + \\ & A_{21}(1 - \exp(-t/\tau_{2r}))\exp(-t/\tau_{1d}) + A_{22}(1 - \exp(-t/\tau_{2r}))\exp(-t/\tau_{2d}) \end{aligned} \qquad \text{r2d2}$$

$$\begin{aligned} I = I_0 + {} & A_{11}(1 - \exp(-t/\tau_{1r}))\exp(-t/\tau_{1d}) + A_{12}(1 - \exp(-t/\tau_{1r}))\exp(-t/\tau_{2d}) + \\ & A_{21}(1 - \exp(-t/\tau_{2r}))\exp(-t/\tau_{1d}) + A_{22}(1 - \exp(-t/\tau_{2r}))\exp(-t/\tau_{2d}) + \\ & A_{13}(1 - \exp(-t/\tau_{1r}))\exp(-t/\tau_{3d}) + \\ & A_{23}(1 - \exp(-t/\tau_{2r}))\exp(-t/\tau_{3d}) \end{aligned} \qquad \text{r2d3}$$

$$\begin{aligned} I = I_0 + {} & A_{11}(1 - \exp(-t/\tau_{1r}))\exp(-t/\tau_{1d}) + A_{12}(1 - \exp(-t/\tau_{1r}))\exp(-t/\tau_{2d}) + \\ & A_{21}(1 - \exp(-t/\tau_{2r}))\exp(-t/\tau_{1d}) + A_{22}(1 - \exp(-t/\tau_{2r}))\exp(-t/\tau_{2d}) + \\ & A_{13}(1 - \exp(-t/\tau_{1r}))\exp(-t/\tau_{3d}) + A_{14}(1 - \exp(-t/\tau_{1r}))\exp(-t/\tau_{4d}) + \\ & A_{23}(1 - \exp(-t/\tau_{2r}))\exp(-t/\tau_{3d}) + A_{24}(1 - \exp(-t/\tau_{2r}))\exp(-t/\tau_{4d}) \end{aligned} \qquad \text{r2d4}$$

$$\begin{aligned} I = I_0 + {} & A_{11}(1 - \exp(-t/\tau_{1r}))\exp(-t/\tau_{1d}) + \\ & A_{21}(1 - \exp(-t/\tau_{2r}))\exp(-t/\tau_{1d}) + \\ & A_{31}(1 - \exp(-t/\tau_{3r}))\exp(-t/\tau_{1d}) \end{aligned} \qquad \text{r3d1}$$

$$\begin{aligned} I = I_0 + {} & A_{11}(1 - \exp(-t/\tau_{1r}))\exp(-t/\tau_{1d}) + A_{12}(1 - \exp(-t/\tau_{1r}))\exp(-t/\tau_{2d}) + \\ & A_{21}(1 - \exp(-t/\tau_{2r}))\exp(-t/\tau_{1d}) + A_{22}(1 - \exp(-t/\tau_{2r}))\exp(-t/\tau_{2d}) + \\ & A_{31}(1 - \exp(-t/\tau_{3r}))\exp(-t/\tau_{1d}) + A_{32}(1 - \exp(-t/\tau_{3r}))\exp(-t/\tau_{2d}) + \end{aligned} \qquad \text{r3d2}$$

$$\begin{aligned} I = I_0 + {} & A_{11}(1 - \exp(-t/\tau_{1r}))\exp(-t/\tau_{1d}) + A_{12}(1 - \exp(-t/\tau_{1r}))\exp(-t/\tau_{2d}) + \\ & A_{21}(1 - \exp(-t/\tau_{2r}))\exp(-t/\tau_{1d}) + A_{22}(1 - \exp(-t/\tau_{2r}))\exp(-t/\tau_{2d}) + \\ & A_{31}(1 - \exp(-t/\tau_{3r}))\exp(-t/\tau_{1d}) + A_{32}(1 - \exp(-t/\tau_{3r}))\exp(-t/\tau_{2d}) + \\ & A_{13}(1 - \exp(-t/\tau_{1r}))\exp(-t/\tau_{3d}) + \\ & A_{23}(1 - \exp(-t/\tau_{2r}))\exp(-t/\tau_{3d}) + \\ & A_{33}(1 - \exp(-t/\tau_{3r}))\exp(-t/\tau_{3d}) \end{aligned} \qquad \text{r3d3}$$

SI.1.3. A two-level system with energy transfer.

Let us consider a simple model system of two species, both representing large groups of the same luminescent centers. Species 1 ground state is level 1, its excited state is level 2. Initially, some part of Species 1 is in its excited state, that is why there is some non-zero population at level 2; population of level 1, n_1 equals $1 - n_2$. Species 2 is in its ground state, level 3, and its excited state (level 4) population is zero. Total population of Species 1 levels is normalized to unity, and the same is total population of Species 2 levels. Energy transfer rate W_{ET} for a given system and given levels is constant, while the rate of particular level population-depopulation depends on the initial populations of both donor and acceptor levels. We would like to know functions $n_2(t)$ and $n_4(t)$ which describe the populations at time t. For simplicity, we will write simply "n_2" and "n_4", keeping in mind that those variables contain a time dependence. Their derivatives are also functions of time and are written as $\partial n_2/\partial t$ and $\partial n_4/\partial t$. We include both direct transfer from Species 1 to Species 2, as well as Species 2 to Species 1 back transfer. On the one hand, it is physically more correct to include back transfer, as it must occur due to the small (or, in this model, exactly zero) energy mismatch (i.e. a perfect resonance condition). On the other hand, such a system of differential equations is actually easier to solve, as the occasional nonlinear terms cancel out.
The dynamics of the level populations will be defined by:

$$\partial n_2/\partial t = - n_2 W_{rad} - n_2 n_3 W_{ET} + n_4 n_1 W_{ET} \quad \text{(S1)}$$

$$\partial n_4/\partial t = - n_4 W_{rad} + n_2 n_3 W_{ET} - n_4 n_1 W_{ET} \quad \text{(S2)}$$

Level 2 is depopulated by a radiative process with the rate of W_{rad}, by the energy transfer to level 4 (which also depends on population of Species 2 ground state, level 3), and is populated by back transfer from level 4 (which also depends on population of Species 1 ground state, level 1). Level 4 is affected by the same processes, but the energy transfer rates are reversed in respect to level 2.

Now, as and $n_3 = 1 - n_4$, and $n_2 = 1 - n_1$, we obtain a system of two differential equations with two unknown variables, n_2 and n_4:

$$\partial n_2/\partial t = - n_2 W_{rad} - n_2 (1 - n_4) W_{ET} + n_4 (1 - n_2) W_{ET} \quad \text{(S3)}$$

$$\partial n_4/\partial t = - n_4 W_{rad} + n_2 (1 - n_4) W_{ET} - n_4 (1 - n_2) W_{ET} \quad \text{(S4)}$$

Opening brackets gives:

$$\partial n_2/\partial t = - n_2 W_{rad} - n_2 W_{ET} + n_2 n_4 W_{ET} + n_4 W_{ET} - n_2 n_4 W_{ET} \quad \text{(S5)}$$

$$\partial n_4/\partial t = - n_4 W_{rad} + n_2 W_{ET} - n_2 n_4 W_{ET} - n_4 W_{ET} + n_2 n_4 W_{ET} \quad \text{(S6)}$$

It is clear that the non-linear terms cancel out, leaving:

$$\partial n_2/\partial t = - n_2 W_{rad} - n_2 W_{ET} + n_4 W_{ET} \quad \text{(S7)}$$

$$\partial n_4/\partial t = - n_4 W_{rad} + n_2 W_{ET} - n_4 W_{ET} \quad \text{(S8)}$$

Summing the two equations gives:

$$\partial n_2/\partial t + \partial n_4/\partial t = \partial (n_2 + n_4)/\partial t = - W_{rad} (n_2 + n_4) \quad \text{(S9)}$$

$$n_2 + n_4 = C \exp(- W_{rad}\, t) \quad \text{(S10)}$$

$$n_4 = C \exp(- W_{rad}\, t) - n_2 \quad \text{(S11)}$$

Here, Eq. S10 is a result of analytical integration of a first-order ordinary differential equation with $(n_2 + n_4)$ as the unknown (Eq. S9).

Substitution of Eq. S11 in Eq. S7:

$$\partial n_2/\partial t = -\, n_2\, W_{\mathrm{rad}} - n_2\, W_{\mathrm{ET}} + (\, C \exp(-\, W_{\mathrm{rad}}\, t) - n_2)\, W_{\mathrm{ET}} \quad \text{(S12)}$$

$$\partial n_2/\partial t = -\, n_2\, W_{\mathrm{rad}} - n_2\, W_{\mathrm{ET}} + \; C \exp(-\, W_{\mathrm{rad}}\, t)\, W_{\mathrm{ET}} - n_2\, W_{\mathrm{ET}} \quad \text{(S13)}$$

$$\partial n_2/\partial t = n_2\, (-\; W_{\mathrm{rad}} - 2\, W_{\mathrm{ET}}) + \; C \exp(-\, W_{\mathrm{rad}}\, t)\, W_{\mathrm{ET}} \quad \text{(S14)}$$

Integration of Eq. (S14), which was done using WolframAlpha, results in :

$$n_2 = c_2 \exp(\, t\; (-\; W_{\mathrm{rad}} - 2\, W_{\mathrm{ET}})\,) + \; 0.5\, C \exp(-\, W_{\mathrm{rad}}\, t) \quad \text{(S15)}$$

Similarly, for n_4, we obtain exactly the same equation. Note that switching n_2 and n_4 in Eq. S8 and Eq. S11 changes nothing, Eqs. S14 and S18 are essentially the same:

$$n_2 = C \exp(-\, W_{\mathrm{rad}}\, t) - \; n_4 \quad \text{(S16)}$$

$$\partial n_4/\partial t = -\, n_4\, W_{\mathrm{rad}} + (C \exp(-\, W_{\mathrm{rad}}\, t) - n_4)\, W_{\mathrm{ET}} \; - n_4\, W_{\mathrm{ET}} \quad \text{(S17)}$$

$$\partial n_4/\partial t = n_4\, (-\, W_{\mathrm{rad}} \; - 2\, W_{\mathrm{ET}}\,) + C \exp(-\, W_{\mathrm{rad}}\, t)\, W_{\mathrm{ET}} \quad \text{(S18)}$$

$$n_4 = c_4 \exp(\, \mathrm{t}\; (-\; W_{\mathrm{rad}} - 2\, W_{\mathrm{ET}})\,) + \; 0.5\, C \exp(-\, W_{\mathrm{rad}}\, t) \quad \text{(S19)}$$

Now, lets consider an equation (named Pulse in OriginPro), which is commonly used in fitting rise-and-decay kinetics:

$$I = I_0\, (1 - \exp(-\, t/\tau_{\mathrm{rise}}\,))\, \exp\, (-\, t/\tau_{\mathrm{decay}}) \quad \text{(S20)}$$

It can be written in terms of rates rather then lifetimes ($\tau = 1/W$). Opening brackets gives:

$$I = I_0\, (1 - \exp(-\, t\, W_{\mathrm{rise}}\,))\, \exp\, (-\, t\, W_{\mathrm{decay}}) \quad \text{(S21)}$$

$$I = I_0\; \exp\, (-\, t\, W_{\mathrm{decay}}) \; - I_0 \exp(-\, t\, W_{\mathrm{rise}}\,)\, \exp\, (-\, t\, W_{\mathrm{decay}}) \quad \text{(S22)}$$

$$I = I_0\; \exp\, (-\, t\, W_{\mathrm{decay}}) \; - I_0 \exp(\, t\, (-\; W_{\mathrm{rise}} \; - \; W_{\mathrm{decay}})) \quad \text{(S23)}$$

Eqs. S23 and S15 differ in coefficients before exponents only. While it is clear that W_{decay} in Eq. S23 is W_{rad} in Eq. S15, an essentially important notion is that Eq. S15 W_{rise} corresponds to <u>2</u> W_{ET} in Eq. S23. (Note the different order of terms in Eqs. S23 and S15). In other words, the rise time corresponds to <u>double</u> the intrinsic energy transfer rate. Given different rates for direct and back transfers, the rise lifetime corresponds to a sum of the two rates.

At $t = 0$, the populations n_2 and n_4 are equal to their initial values n_{20} and n_{40}, respectively, of which n_{40} is zero. From Eq. S10:

$$n_{20} + n_{40} = n_{20} \; = C \exp(0) = C \quad \text{(S24)}$$

Next, from Eqs. S15 and S19:

$$n_2(0) = n_{20} = c_2\, \mathrm{e}^0 + 0.5\, C\, \mathrm{e}^0 = \; c_2 + 0.5\, C = \mathrm{c}_2 + 0.5\, n_{20} \quad \text{(S25)}$$

$$c_2 = 0.5\, n_{20} \quad \text{(S26)}$$

$$n_4(0) = n_{40} = c_4\, \mathrm{e}^0 + 0.5\, C\, \mathrm{e}^0 = \; c_4 + 0.5\, C = 0 \quad \text{(S27)}$$

$$-\, c_4 = 0.5\, C = -\, 0.5\; n_{20} \quad \text{(S28)}$$

Consequently:

$$n_2 = 0.5\, n_{20} \exp\, (-\, t\, W_{\mathrm{rad}}) \; + \; 0.5\, n_{20} \exp\, (\, t\, (-\, W_{\mathrm{rad}} \; - 2\, W_{\mathrm{ET}})) \quad \text{(S29)}$$

$$n_4 = 0.5\, n_{20} \exp\, (-\, t\, W_{\mathrm{rad}}) \; - \; 0.5\, n_{20} \exp\, (\, t\, (-\, W_{\mathrm{rad}} \; - 2\, W_{\mathrm{ET}})) \quad \text{(S30)}$$

Eq. S29 is two-exp decay with lifetimes of $1/W_{rad}$ and $1/(W_{rad} + 2\,W_{ET})$, while Eq. S30 is rise-and-decay with decay lifetime of $1/W_{rad}$ and rise lifetime of $1 / (2\,W_{ET})$.
In other words, while the overall kinetics of n_2 and n_4 is algebraically the same, the initial values of those two populations produce different actual kinetics, namely two-component decay and rise-and-decay, respectively. Numerical simulations confirm this conclusion. Plots of simulated n_2 and n_4 dynamics can be fit with exactly the same equation (the sum of two exponential decays), where the absolute values of the coefficients before the exponents are half of the initial population of level 2, n_{20}; the only difference is sign before the $(W_{rad} + 2\,W_{ET})$ part.

SI.2. Overfitting in multiexponential decay

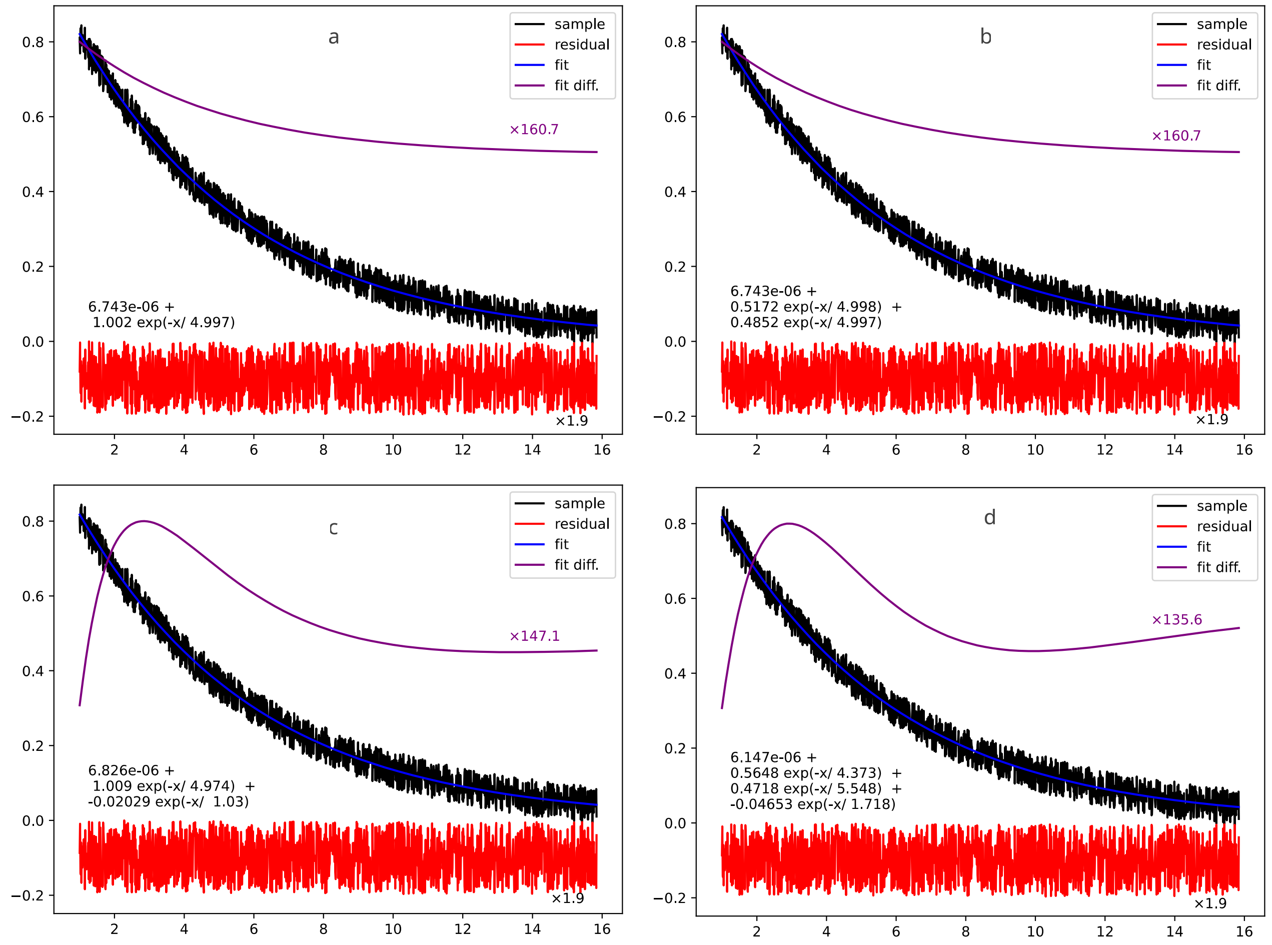

Fig. S1. A noisy monoexponential decay curve fitted with one, two or three components. Sample: A=1, τ=5, random noise amplitude ±0.05. “Fit” is the fitted curve. “Fit diff.” is the difference between the idealized $y = A\exp(-x/\tau)$ noise-free curve and the fitted cure. The multipliers used to get the visible proportions are specified. The fitted equations are specified.

In Figure S1, the same idealized monoexponential decay sample was fitted with different number of components. The true values are: A=1, τ=5, random noise amplitude ±0.05. The random noise spanned below and above the ideal curve, meaning that vertical offset should have been zero. While it was indeed small (about 6e-6 – 7e-6), it was much larger than the numerical precision “zero”, which was in the order of 1e-16.

From Fig. S1 a and b, it is clear that, in this particular example, overfitting with positive components is easily identified. The two components in panel b have comparable amplitudes and

basically the same lifetime. The differences between the fitted curve and the ideal noise-free curve ("fit diff.") in panels a and b are of the same shape. I.e. both fitted curves are identical.

The fit result in Fig. S1 c was produced from the fit result in Fig. S1 a: the guess contained the *A*=1.009, τ=4.974 decay from panel a, and another component with *A*=1 and τ=2. The respective fitting completed with negative amplitude at the second component, while the lifetime in the first component deviated more from the ideal τ=5. In Fig. S1 d, with three components (one negative), the deviations are even larger. In other words, despite the example clearly looking as a decay profile (no need to add negative components manually), the negative component showed up anyway.

It must be pointed out that the lower is the noise, the less likely are the described effects to show up.

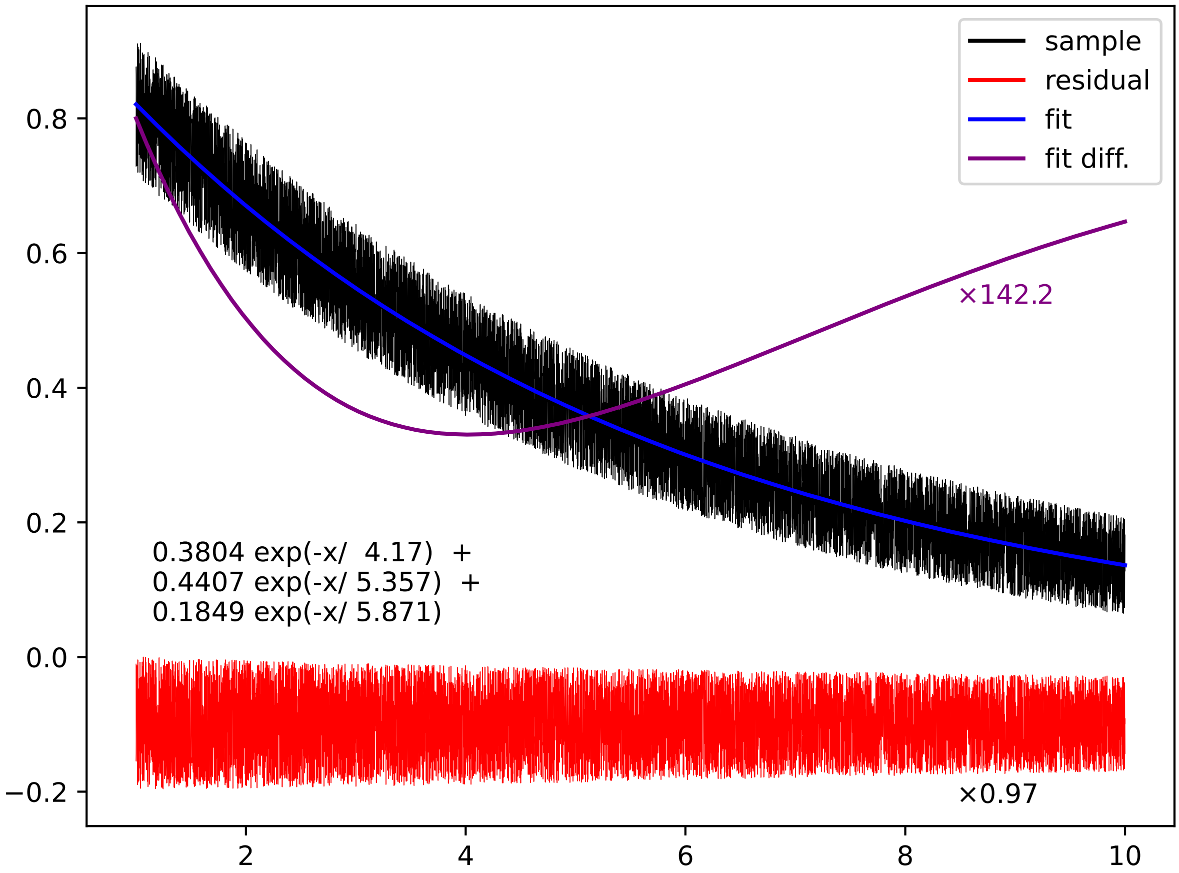

Fig. S2. A noisy monoexponential decay curve fitted with three components. Sample: *A*=1, τ=5, noise amplitude decays monoexponentially with τ=27.

Under some less ideal conditions, with a real sample, the convergence of lifetimes seen in Fig. S1 b is less pronounced. The spare components do approach each other, but might as well differ by 10-20% and more in terms of their lifetimes. Such conditions were approximated in with a changing noise amplitude. Namely, in Fig. S2, the noise amplitude decays monoexponentially with τ=27. As a result, the fit with three components resulted in distinctly different lifetimes, despite the fact that the true (noise-free) curve was still monoexponential.

SI.3. Structural information

This section contains the coordination geometry of the La site, the X-ray diffraction (XRD) patterns of the samples, and the selected transmission electron microscopy (TEM) images.

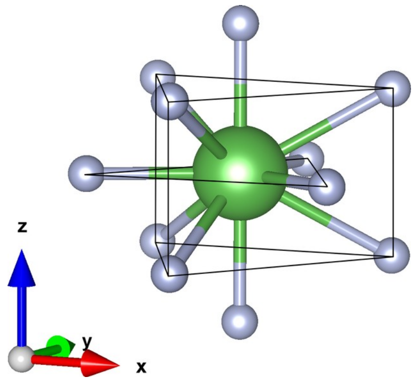

Figure SI.3. Coordination geometry of the La site in $P\,6_3/\,m\,m\,c$ (space group nr. 194) LaF_3. Traced are the triangular prism and the equatorial triangle formed by the F atoms of the surround. The C_3 rotation axis coincides with the *z* coordinate axis.

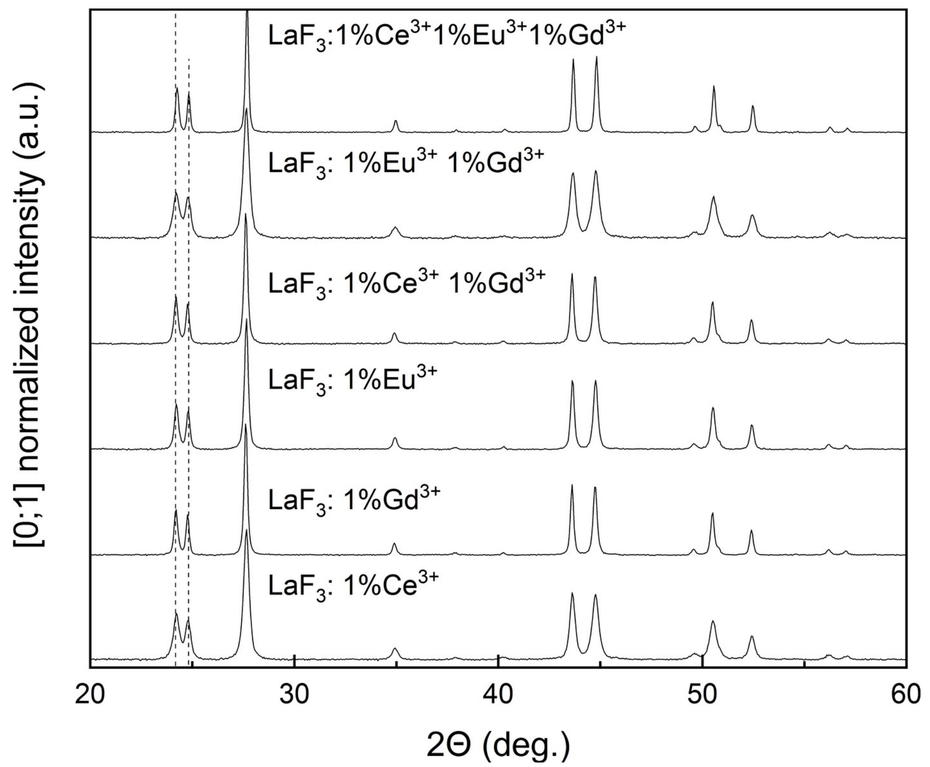

Figure SI.4. X-ray diffraction (XRD) patterns of the prepared samples.

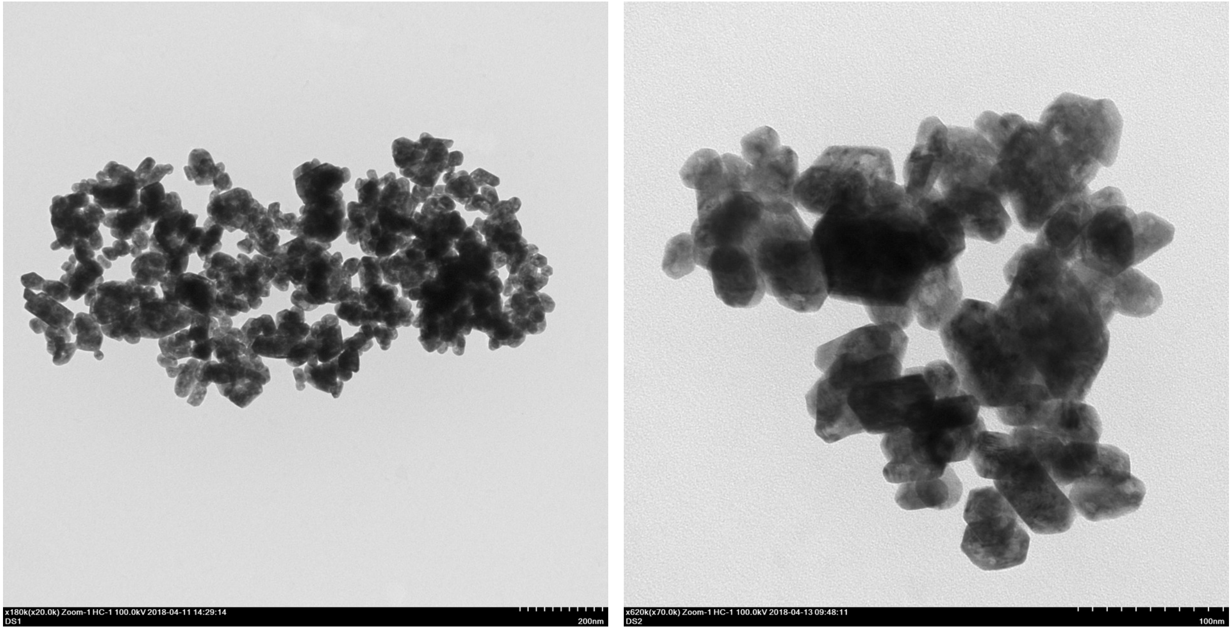

Figure SI.5. Transmission electron microscopy (TEM) images of LaF_3: 1% Gd^{3+} (left) and LaF_3: 1% Eu^{3+} (right).

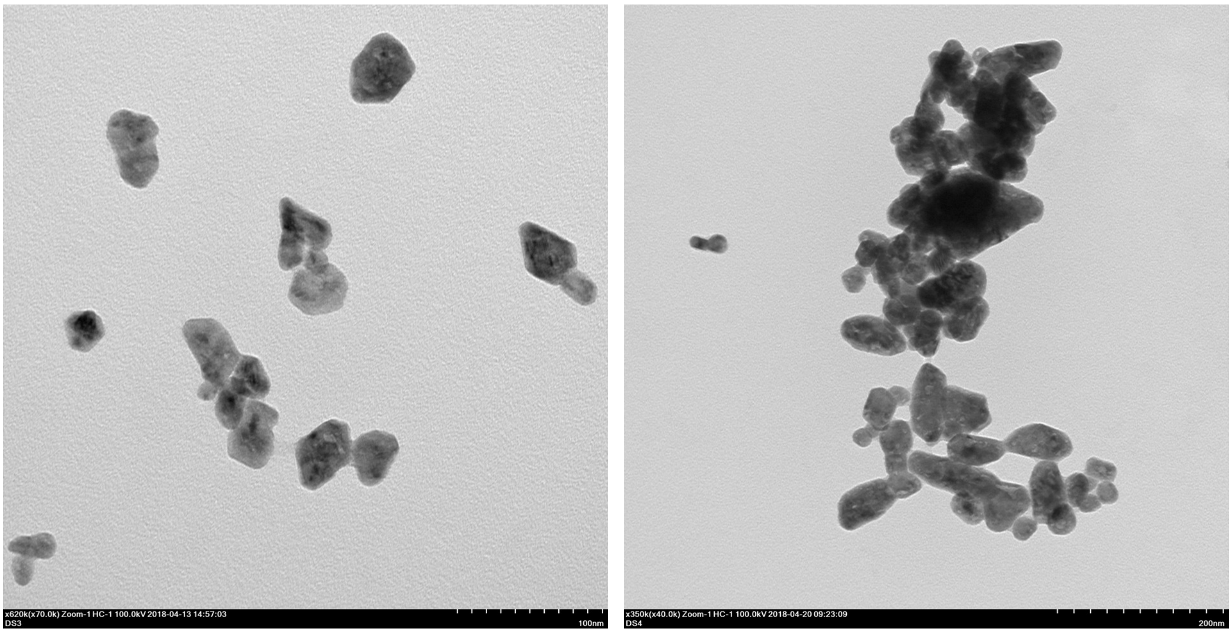

Figure SI.6. Transmission electron microscopy (TEM) images of LaF_3: 1% Gd^{3+}, 1% Eu^{3+} (left) and LaF_3: 1% Ce^{3+}, 1% Gd^{3+}.

SI.4. Alleged Ce^{3+} contamination

Alternative explanation of the additional rise component in the LaF^3:Gd^{3+} emission of Gd^{3+} would be presence of small amount of Ce^{3+} in the samples. In 99.99% La_2O_3, Ce is the most likely contaminant at less than 0.01%.

In a LaF_3: 1%Eu, 1%Ce sample obtained in the same series of samples as the LaF_3: 1%Gd in question, a broad emission band of Ce^{3+} f-d emission is observed in the 280-320 nm range, peaking at 303 nm. Consequently, strong overlap of Ce^{3+} f-d excitation band with the 272 nm $^6I \leftrightarrow {}^8S_{7/2}$ band of Gd^{3+} is expected. Ce^{3+} f-d emission overlaps with the 312 nm $^6P \leftrightarrow {}^8S_{7/2}$ band of Gd^{3+}. Thus, Gd^{3+} $^6I \rightarrow Ce^{3+}$ f-d $\rightarrow Gd^{3+}$ ^6P energy transfer is possible. Such mechanism is supported by the fact that the 272 / 312 nm decay profiles of Gd^{3+} in LaF_3: 1%Gd, 1%Ce do not show any rise, indicating involvement of Ce^{3+} in Gd^{3+} ^6P dynamics. Energy transfer processes with Ce^{3+} as one of the parts must be very fast due to the allowed f-d transitions of the latter. However, the concentration of Ce^{3+} in allegedly contaminated LaF_3: 1%Gd is at least two or three orders of magnitude lower than that in LaF_3: 1%Gd, 1%Ce, meaning that the rates of energy transfer processes involving Ce^{3+} must be proportionally lower.

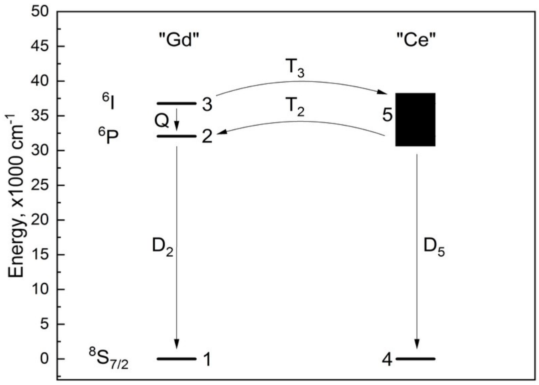

Fig. SI.7. Gd^{3+}-Gd^{3+} energy transfer scheme

SI.4.1 The model system: Gd → Ce → Gd energy transfer

In order to describe the interaction of Gd^{3+} ions with the contaminant Ce^{3+}, the energy level scheme shown in Fig. SI.7 was established. Here, excited manifold of Ce^{3+} is represented as a single level 5, which is involved in two energy transfers with Gd^{3+}. Level 5 decay rate to level 4 is D_5, while Gd^{3+} radiative decay rate is D_2.The initial values of $n_{1\text{-}3}$ were 0, 0, 1.

$$W_{T3} = T_3 \,(\, n_3\, n_4 - \delta_3\, n_5\, n_1 \,) \tag{S31}$$
$$W_{T2} = T_2 \,(\, n_5\, n_1 - \delta_2\, n_2\, n_4 \,) \tag{S32}$$
$$\partial n_1/\partial t = D_2\, n_2 - W_{T2} + W_{T3} \tag{S33}$$
$$\partial n_2/\partial t = -D_2\, n_2 + Q\, n_3 + W_{T2} \tag{S34}$$
$$\partial n_3/\partial t = -Q\, n_3 - W_{T3} \tag{S35}$$
$$\partial n_4/\partial t = D_5\, n_5 + W_{T2} - W_{T3} \tag{S36}$$
$$\partial n_5/\partial t = -D_5\, n_5 - W_{T2} + W_{T3} \tag{S37}$$

Numerous options have been tested, namely unidirectional (3 → 5 → 2) and bidirectional (3 ↔ 5 ↔ 2) energy transfers (controlled by the respective δ parameters in Eq. S31-S32, set to either 1 or 0) , D_5 equal zero and not equal zero, T_3 equal to T_2 or not equal to T_2. The initial population of levels n_4 and n_5 was either x, 0 (ground state “Ce”) or 0, x (excited state “Ce”), were x was 0, 0.1, 0.01 or variational. Total number of variational parameters changed accordingly. In most of the cases, despite continuous efforts and hundreds of initial guesses, we were not able to obtain a good fit. The calculations converged with $^6I \rightarrow {}^6P$ lifetime of 330-370 μs, many of them reducing the

concentration of "Ce" to very small values. The overall fit quality was similar to Fig. 2 a of the main text, or worse.

The summary of the few successful fits is given in table below, in the form of lifetimes. Although the fits converged with values of Gd^{3+} decay lifetimes similar to those from the multiexponential fits, they assume large content of "Ce" and no emission from it, which is rather not feasible, and illustrate inconsistency of the Ce^{3+} contamination model. Thinking simply, an empty excited level of allowed d-f transition of Ce^{3+} at energy similar to that of Gd^{3+} $^6P \leftrightarrow {}^8S_{7/2}$ transition should rather quench the former, not sensitize it.

Contamination with Ce^{3+} cannot explain the 700 μs rise component of the Gd^{3+} $^6P \rightarrow {}^8S_{7/2}$ emission kinetics under 272 nm excitation.

Table SI.1. Results of successful fits from the Ce contaminant model

$n_{4\text{-}0}$, dim.less	τ_{D2}, μs	τ_Q, μs	τ_{D5}, μs	τ_{T2}, μs	τ_{T3}, μs
0.100	12652	157	∞	14306	61.7
0.189	12592	99.0	∞	9691	43.8
0.289	12639	70.0	∞	17168	38.8

SI.5. A model with two Gd species and one Ce species

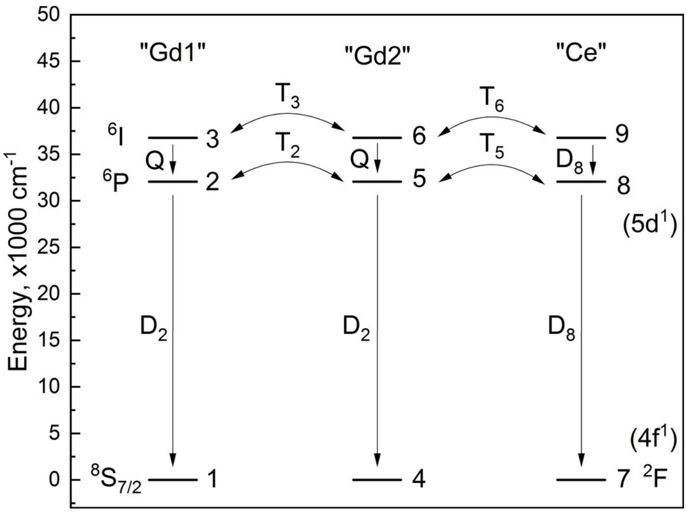

Fig. SI.8. The Gd-Gd-Ce system of levels and transitions

This model build upon an assumption that, in LaF_3:Ce^{3+},Gd^{3+} some Gd-Gd interaction must come in play, similarly to the LaF_3:Gd^{3+} case described in the main text. The respective system of interactions is visualized in Fig. SI.8 and comprises the following equations:

$$W_{T3} = T_3\,(\,n_3\,n_4 - n_6\,n_1\,) \quad \text{(S38)}$$
$$W_{T2} = T_2\,(\,n_2\,n_4 - n_5\,n_1\,) \quad \text{(S39)}$$
$$W_{T6} = a_8\,\mathrm{T}_3\,(\,n_6\,n_7 - n_8\,n_4\,) \quad \text{(S40)}$$
$$W_{T5} = a_8\,\mathrm{T}_2\,(\,n_5\,n_7 - n_8\,n_4\,) \quad \text{(S41)}$$
$$\partial n_1/\partial t = D_2\,n_2 + W_{T2} + W_{T3} \quad \text{(S42)}$$
$$\partial n_2/\partial t = -D_2\,n_2 + Q\,n_3 - W_{T2} \quad \text{(S43)}$$
$$\partial n_3/\partial t = -Q\,n_3 - W_{T3} \quad \text{(S44)}$$
$$\partial n_4/\partial t = D_2\,n_5 - W_{T2} - W_{T3} + W_{T5} + W_{T6} \quad \text{(S45)}$$
$$\partial n_5/\partial t = -D_2\,n_5 + Q\,n_6 + W_{T2} - W_{T5} \quad \text{(S46)}$$
$$\partial n_6/\partial t = -Q\,n_6 + W_{T3} - W_{T6} \quad \text{(S47)}$$

$$\partial n_7/\partial t = \quad D_8\, n_8 \qquad\qquad - W_{T5} \qquad (-P\, n_7) \qquad \text{(S48)}$$
$$\partial n_8/\partial t = -D_8\, n_8 \quad + \delta_9\, D_8\, n_9 \quad + W_{T5} \quad - W_{T6} \quad (+P\, n_7) \qquad \text{(S49)}$$
$$\partial n_9/\partial t = \qquad - \delta_9\, D_8\, n_9 \qquad + W_{T6} \quad (+P\, n_7) \qquad \text{(S50)}$$

Sum of n_2 and n_5 populations multiplied by an amplitude and shifted by an intensity offset was the output signal, fitted to the experimental decay profile. The Ce^{3+} decay rate was kept constant, $D_8 = 1/0.0292$ (μs^{-1}). The Gd-Ce energy transfer rates were considered linearly proportional to the respective Gd-Gd decay rates. In a virtual case of the Gd-Gd and Gd-Ce pairs of atoms interacting with each other at the same distance, the rates would have been proportional to the ratio of the electric dipoles of the participating transitions. It is a bit of a stretch to assume the same coefficient for the two rates. However, it is still a feasible approximation, as we were not looking for exact values of the rates. D_2, Q, T_2, T_3 and a_8 were fit parameters. The ratio between Gd1 and Gd2 was another fit parameter. With total amplitude, intensity offset and time offset, the total number of parameters in this model was 9, the same as in a 4-exp decay (4x lifetime, 4x amplitude, plus intensity offset).

The problem of initial values in this model was also solved using solution of a differential equation system, as described in the main text. Namely, given the fact that level 9 is the one mostly populated upon excitation (due to huge difference in oscillator strengths of Ce^{3+} and Gd^{3+} transitions), a pump was added at Ce species (the terms in brackets in Eq. S48-S50). The pump final state was either level 8 or level 9, depending on the assumed excitation wavelength. At every step of the main fitting, a subroutine was initialized with the same parameters, in which several solutions of the system with the pump were performed (at a 0 to 2 μs range, with 0.01 ns step), fitting the value of the pump in such a way that sum of the excited state populations was 1 (in other words, the residual function to be minimized was $n_{2e}+n_{3e}+n_{5e}+n_{6e}+n_{8e}+n_{9e}-1$, the "e" subscripts indicating the end of the intermediate solution). Thus, the subroutine produced the level populations in a system of ODE that was continuously excited for 2 μs, such that total population of the excited states was one. Next, this system was solved in pulsed mode, without the pump. Initially, the sum of n_1 and n_4 populations was 224288, and n_7 was 224288.

While such a model was able to provide a series of good fits with reasonable parameters, it had a significant flaw. It was impossible to achieve a set of parameters that would satisfy both the 250 nm excitation case and the 272 nm excitation. In that regards, the model with two kinds of independent Ce-Gd pairwise interactions was much more successful, and was selected as the main model.

SI.6. The effect of temporal offset on the fits of LaF_3: 1%Gd^{3+}, 1%Ce^{3+} emission kinetics.

In the rise-and-decay fitting, there is an essential variable parameter – the temporal offset. The post-pulse evolution of the system by definition starts at zero of its internal (model) time. However, this zero time point does not necessarily correspond to the experimental $t = 0$ point. The temporal offset parameter t_{0i} basically says that, from the point of view of this particular ODE system, the experimental data points start at t_{0i} model time. In Table SI.2, the solutions labeled as "shifted" correspond to the whole data sets (starting at the experimental time 831 μs), shifted along time axis so that they start at experimental time zero ($t_0 = 0$). The solutions marked as "cropped" kept the original experimental axis, but the first 500 μs of the curve were discarded, and the data points started at $t_0 = 1231$ μs experimental time. For the shifted (whole) data sets, additional exponential decay was added.

For the 250 nm excitation, the cropped sample converged with t_{0i} of about –100 µs, indicating that the that the process represented by the experimental data points starts at $t_0 + t_{0i} = 1231–100 = 1131$ µs of the model time. The shifted counterpart converged with t_{0i} of about 1169 µs, indicating in this case the process represented by the experimental data points started at $t_0 + t_{0i} = 0+1169 = 1169$ µs of the model time. Thus, both fits agree well on the fact that the process represented by the experimental data points began after about 1.1 ms of the model time.

For the 272 nm excitation, the cropped sample converged with t_{0i} of about –148 µs ($t_0 + t_{0i} =$ 1083 µs), while the shifted sample converged with t_{0i} of about 1080 µs ($t_0 + t_{0i} = 1080$ µs). The two $t_0 + t_{0i}$ values agree even better in this case, indicating that the process represented by the experimental data points began after about 1.08 ms of the model time.

The fits of the cropped and shifted samples exhibit quite similar final parameters (Table SI.2).

Table SI.2. Fitting results for the LaF_3: 1%Gd^{3+}, 1%Ce^{3+} sample. The parameters correspond to the model mentioned in Section 3.4.4 of the main text.

$\lambda_{ex.}$, nm	Exp. data type	ODE set								Exponential decay	
		t_{0i}, µs	A_{ODE}	X_{12}	τ_{nrad}, µs	τ_{rad}, µs	T_2, s^{-1}	T_3, s^{-1}	X_{P2}	A_{decay}	τ_{decay}, µs
250	cropped	-100	6735	0.0485	100	12982	$7.40\cdot10^{-5}$	$2.79\cdot10^{-1}$	0.0150		
250	shifted	1182	7384	0.0527	107	13008	$7.34\cdot10^{-5}$	$2.50\cdot10^{-1}$	0.0161	0.0368	114
272	cropped	-102	800	0.492	104	12799	$1.37\cdot10^{-4}$	$1.90\cdot10^{-1}$	0.0128		
272	shifted	1158	817	0.553	101	12831	$1.26\cdot10^{-4}$	$2.09\cdot10^{-1}$	0.0131	0.0818	105
250, 272	cropped	-100	6944	0.0430	104	13094	$8.94\cdot10^{-5}$	$2.21\cdot10^{-1}$	0.0189		
	cropped	-100	677	0.574							

In Table SI.2, both A_{decay} and A_{ODE} are amplitudes, but they have different units and meanings, and are incomparable.
