# Supplementary material for: LaF3 doped with Ce/Gd/Eu: energy transfer and excitation dependence of photoluminescence rise-and-decay kinetics
Source: Front Chem. 2025 Apr 22;13:1501039. doi: 10.3389/fchem.2025.1501039 (PMC12052759; doi:10.3389/fchem.2025.1501039)
Supplement: Supplementary file 3 [file DataSheet1.pdf]

Each row in a the tables corresponds to a single fit result.
The trace files are attached as a separate archive file.

Some of the traces where not fitted from zero time, the initial part was cropped off.
The respective value is t_crop.

Cells contain raw 64 bit floats; the number of displayed digits is reduced.

Emission at 694 nm	exc. wl. (nm)	t_crop	t_offset (t_0)	vertical offset	function	Ar1d1	Ar1d2	Ar1d3	Ar2d1	Ar2d2	Ar2d3	Ar3d1	Ar3d2	Ar3d3
LaF3Eu394_694.trace.txt	394	830	760.196	-0.0210301	r2d3				0.24775	0.0662461	0.364964	0.374915	0.0998716	0.549658
LaF3CeEu250_694.trace.txt	250		719.561	-0.0144601	r2d3				0.163124	0.154729	0.752809	0.0300842	0.0303388	0.164311
LaF3CeEu394_694.trace.txt	394		807.984	-0.0191782	r3d3	0.166226	0.152643	0.546338	0.0491867	0.0378966	0.113058	0.378858	0.0619405	0.238344
LaF3GdEu272_694.txt	272		732.167	-0.0295936	r2d3				0.450218	0.171981	0.527633	0.319076	0.121768	0.37164
LaF3GdEu394_694.trace.txt	394		779.454	-0.0240818	r3d3	0.351669	0.136477	0.490265	0.0638799	0.0234722	0.0860684	0.512374	0.185894	0.677255
LaF3CeGdEu250_694.trace.txt	250		773.638	-0.0656983	r3d3	0.060364	0.160208	0.329469	0.015816	0.0415865	0.0854813	0.294606	0.781247	1.59134
LaF3CeGdEu272_694.trace.txt	272		760.73	-0.0273682	r2d2				0.360338		0.729068	0.12549		0.253903
LaF3CeGdEu394_694.trace.txt	394	40	-144.292	-0.0283649	r2d2				0.135258		0.867918	0.0968681		0.62591

Emission at 618 nm	exc. wl. (nm)	t_crop	t_offset (t_0)	vertical offset	function	Ar1d1	Ar1d2	Ar1d3	Ar2d1	Ar2d2	Ar2d3	Ar3d1	Ar3d2	Ar3d3
LaF3Eu394_618.trace.txt	394	830	681.163	-0.0272927	r2d3				0.16084	0.0351763	0.598046	0.0320485	0.026429	0.559041
LaF3Eu394_618.trace.txt	394	830	673.742	-0.0271427	r2d3				0.149553	0.0327133	0.6179	0.137768	0.0287737	0.566137
LaF3CeEu250_618.trace.txt	250		687.315	-0.0139697	r2d3				0.240854	0.174007	0.693931	0.0209515	0.0149372	0.0602783
LaF3CeEu394_618.trace.txt	394		764.947	-0.0192913	r3d3	0.0955359	0.118328	0.736187	0.0112411	0.0139281	0.0866756	0.0225081	0.0279402	0.173714
LaF3GdEu272_618.trace.txt	272		619.731	-0.0411272	r2d3				0.361746	0.138663	0.714168	0.264776	0.0964146	0.510708
LaF3GdEu394_618.trace.txt	394		727.439	-0.027629	r2d3	0.0429227	0.101433	0.859129				2.53413	0.513594	0.562609
LaF3CeGdEu250_618.trace.txt	250		721.465	-0.0641342	r2d3	0.0982171	0.245634	0.532855				0.255238	0.638335	1.38475
LaF3CeGdEu272_618.trace.txt	272		512.94	-0.0186021	r2d3	0.478135	0.473001	0.612141				0.0901093	0.0891417	0.115364
LaF3CeGdEu394_618race.txt	394		-198.83	-0.027224	r2d3				0.844331	0.0631504	0.859135	0.551074	0.0418389	0.55914

Below, there are cases where two columns are labeled “τd2” or “τd3”
The lifetimes were grouped together by the similarities in their values.
A lifetime that is τd2 (in the formula/fit) can be more suited in different columns – depending on its value.

							Emission at 694 nm	
τr1	τr2	τr3	τd1	τd2	τd2	τd3		exc. wl. (nm)
	35.8	5408	495	1254		13321	LaF3Eu394_694.trace.txt	394
	41.2	2787	450		5520	12327	LaF3CeEu250_694.trace.txt	250
6.492	46.1	3029	449		8629	12773	LaF3CeEu394_694.trace.txt	394
	41.9	7954	635	1703		14673	LaF3GdEu272_694.txt	272
24.767	99.5	4751	503	1079		12598	LaF3GdEu394_694.trace.txt	394
0.619	39.6	6972	297		3800	14740	LaF3CeGdEu250_694.trace.txt	250
	21.8	5105	527			14195	LaF3CeGdEu272_694.trace.txt	272
	63.6	2799	406			12670	LaF3CeGdEu394_694.trace.txt	394

							Emission at 618 nm	
τr1	τr2	τr3	τd1	τd2	τd2	τd3		exc. wl. (nm)
	57.891	5492.59	565.043		7469.33	13396	LaF3Eu394_618.trace.txt	394
	61.5883	5106.82	477.926	1173.17		13305.5	LaF3Eu394_618.trace.txt	394
	45.1864	2520.18	471.208		6855.08	12618.9	LaF3CeEu250_618.trace.txt	250
19.4415	94.9804	2520.81	561.969		8023.58	12509.6	LaF3CeEu394_618.trace.txt	394
	74.0912	7439.95	555.834	1241.13		14735.8	LaF3GdEu272_618.trace.txt	272
40.6163		4586.02	255.377	670.114		12597.7	LaF3GdEu394_618.trace.txt	394
32.5677		6869.28	389.032		3651.51	14651.8	LaF3CeGdEu250_618.trace.txt	250
13.4681		9527.82	234.761	659.503		13799.8	LaF3CeGdEu272_618.trace.txt	272
	174.971	2611.33	154.626		1992.5	12646.2	LaF3CeGdEu394_618race.txt	394

Emission at 613 nm														
	exc. wl. (nm)	t_crop	t_offset (t_0)	vertical offset		Ar1d1	Ar1d2	Ar1d3	Ar1d4					
LaF3CeEu250_613.trace.txt	250		716.085	-0.00594227	r1d4	0.421081	0.39552	0.378722	0.917938					
LaF3CeGdEu272_613.trace.txt	272		807.722	-0.00802701	r1d4	0.185009	2.22349	0.831199	1.6104					
						Ar1d1	Ar1d2	Ar1d3	Ar2d1	Ar2d2	Ar2d3	Ar3d1	Ar3d2	Ar3d3
LaF3Eu394_613.trace.txt	394	830	715.767	-0.0165097	r2d3	0.309979	0.0948509	0.475392				0.16447	0.0474935	0.246642
LaF3CeEu394_613.trace.txt	394		772.169	-0.0135897	r3d3	0.271241	0.142109	0.570015	0.0384402	0.0201522	0.080782	0.0364172	0.0191081	0.0767196
LaF3GdEu272_613.trace.txt	272		603.929	-0.0174246	r2d3	0.559401	0.282143	0.468336				0.240125	0.119472	0.21883
LaF3GdEu394_613.trace.txt	394		762.435	-0.0156609	r2d3	0.00181941	0.383922	0.642994				3.39108	0.0448271	0.215585
LaF3CeGdEu250_613.trace.txt	250		800.687	-0.0435901	r2d2	0.323238		0.741808				0.025487		0.483177
LaF3CeGdEu394_613race.txt	394		-176.378	-0.019046	r2d2	0.304477		0.830345				0.089318		0.244568

Emission at 519 nm														
	exc. wl. (nm)	t_crop	t_offset (t_0)	vertical offset		Ar1d1	Ar1d2	Ar1d3	Ar2d1	Ar2d2	Ar2d3	Ar3d1	Ar3d2	Ar3d3
LaF3Eu394_591.trace.txt	394	830	740.001	-0.0399877	r2d3	0.30992	0.00136298	0.355581				1.97613	1.55382	1.3143
LaF3CeEu250_591.trace.txt	250		772.576	-0.0210495	r3d2		0.661458	0.0989682		0.0598823	0.0464116		0.286182	1.24519
LaF3CeEu394_591.trace.txt	394		754.726	-0.0245624	r3d2		0.0750918	0.680314		0.12211	0.0145299		0.0729117	0.754592
LaF3GdEu272_591.trace.txt	272		735.62	-0.0846696	r2d3	0.289384	0.0959269	0.360634				2.62041	1.69972	1.90532
LaF3GdEu394_591.trace.txt	394		754.649	-0.0389249	r2d3	0.00192584	0.200785	0.694754				2.48244	0.337836	1.20937
LaF3CeGdEu250_591.trace.txt*	250		753.89	-0.078312	r2d3	0.0438426	0.00276676	0.391246				0.800332	1.30327	1.62426
LaF3CeGdEu272_591.trace.txt	272		735.689	-0.0577067	r2d3	0.207096	0.306377	0.32144				0.987958	1.46343	1.51849
LaF3CeGdEu394_591race.txt	394		-162.585	-0.0304156	r2d3	0.128496	0.156547	0.363825				0.41059	0.499555	1.16723

							Emission at 613 nm	
τr1			τd1	τd2	τd3	τd4		exc. wl. (nm)
33.197			535.28	1437.36	4758.95	12256.3	LaF3CeEu250_613.trace.txt	250
5.47238			273.661	731.413	2314.51	13914.8	LaF3CeGdEu272_613.trace.txt	272
τr1	τr2	τr3	τd1	τd2	τd2	τd3		
48.1869		5269.22	507.484	1096.71		13215.7	LaF3Eu394_613.trace.txt	394
20.053	118.496	5269.15	599.296		5986.25	12430.6	LaF3CeEu394_613.trace.txt	394
79.0178		23143.1	548.163	1425.18		13757.4	LaF3GdEu272_613.trace.txt	272
29.2655		4585.26	224.264	800.974		12418.7	LaF3GdEu394_613.trace.txt	394
6.51408		6543.18	507.08			14984.5	LaF3CeGdEu250_613.trace.txt	250
70.1448		2432.91	574.745			12531.6	LaF3CeGdEu394_613race.txt	394

							Emission at 519 nm	
τr1	τr2	τr3	τd1	τd2		τd3		exc. wl. (nm)
44.9245		4684.34	420.776	1271.75		13450.1	LaF3Eu394_591.trace.txt	394
16.1188	83.4101	1721.72		1156.95		11981.9	LaF3CeEu250_591.trace.txt	250
28.8065	107.087	2542.83		1909.49		12152.3	LaF3CeEu394_591.trace.txt	394
51.4668		6058.98	477.41	1641.09		15042	LaF3GdEu272_591.trace.txt	272
38.6826		4567.12	257.824	1102.35		12697.1	LaF3GdEu394_591.trace.txt	394
24.9419		4983.27	333.311	1756.34		15283.7	LaF3CeGdEu250_591.trace.txt*	250
33.8859		4084.41	447.22	1519.4		14308.9	LaF3CeGdEu272_591.trace.txt	272
78.4746		2461.45	737.304	1590.14		12706.8	LaF3CeGdEu394_591race.txt	394

Emission at 583 nm														
	exc. wl. (nm)	t_crop	t_offset (t_0)	vertical offset		Ar1d1	Ar1d2	Ar1d3	Ar1d4		Ar2d1	Ar2d2	Ar2d3	Ar2d4
LaF3CeGdEu394_583race.txt	394		-49.6667	-0.00310678	r2d4	0.454353	0.156515	0.232334	0.0395883		0.0120804	0.429407	0.399074	0.00670664
LaF3CeEu394_583.trace.txt	394		438.224	-0.000782776	r1d4	0.298142	2.00754	1.59717	0.19882					
						Ar1d1	Ar1d2	Ar1d3	Ar2d1	Ar2d2	Ar2d3	Ar3d1	Ar3d2	Ar3d3
LaF3CeEu250_583.trace.txt	250		749.297	-2.39E-06	r2d3	0.373531	0.54254	0.0153488	0.147529	0.095558	0.0427951			
LaF3CeGdEu272_583.trace.txt	272	850	742.831	-0.00350531	r2d3	0.272016	0.452488	0.166101	0.188669	0.139368	0.0488193			
LaF3GdEu394_583.trace.txt	394	860	530.286	-0.00064988	r2d2		0.953464	0.044327		0.345248	0.0200223			
LaF3Eu394_583.trace.txt	394	860	694.578	-0.000642712	r3d2		0.696954	0.0238		0.311984	0.0169722		0.149102	0.016775
LaF3GdEu272_583.trace.txt	272	850	746.967	-0.00501835	r3d2		0.56323	0.0970462		0.25023	0.0780534		0.397561	0.102329
LaF3CeGdEu250_583.trace.txt	250	860	772.269	-0.00404314	r3d3	0.242935	0.377507	0.156832	0.128396	0.135312	0.0445899	0.0653935	0.0646507	0.0616045

Emission at 552 nm														
	exc. wl. (nm)	t_crop	t_offset (t_0)	vertical offset		Ar1d1	Ar1d2	Ar1d3	Ar2d1	Ar2d2	Ar2d3	Ar3d1	Ar3d2	Ar3d3
LaF3Eu394_552.trace.txt	394	10000		0.00038825	d1*	1.41063								
LaF3Eu394_552.trace.txt	394	860	719.226	0.000377026	r3d1		0.697608			0.364469			0.354269	
LaF3GdEu272_552.trace.txt	272		763.777	-0.00274026	r3d2		0.569541	0.0437117		0.319154	0.0403992		0.420576	0.115715
LaF3GdEu394_552.trace.txt	394	860	643.938	1.35E-07	r3d2		0.861241	0.0142824		0.331497	0.0140987		0.135834	0.0222186
						Ar1d1	Ar1d2	Ar1d3	Ar1d4		Ar2d1	Ar2d2	Ar2d3	Ar2d4
LaF3CeEu250_552.trace.txt	250		754.437	0.000260621	r2d4	0.255364	0.484418	0.192564	0.00177771		0.135347	0.0932157	0.0864319	0.00176139
LaF3CeEu394_552.trace.txt	394		739.506	4.65E-05	r2d4	0.18096	0.497335	0.233544	0.00160863		0.158668	0.00362547	0.144559	0.000657163
LaF3CeGdEu250_552.trace.txt	250		777.87	-0.00139389	r2d4	0.812654	0.747188	0.154761	0.0743287		0.462736	0.0264279	0.245004	0.00937364
LaF3CeEu250_552.trace.txt	250		756.492	0.000561623	r2d3	0.330468	0.580983	0.013277			0.194017	0.109695	0.0102731	
LaF3CeGdEu272_552.trace.txt	272	840	745.667	-0.000269776	r2d3		0.36631	0.466904	0.0512983			0.175783	0.193913	0.0521996
LaF3CeGdEu394_552race.txt	394		-39.0768	0.000381789	r2d2		0.400926	0.422082				0.242938	0.210789	
LaF3CeGdEu394_552race.txt	394	5000		0.000196027	d1+d1*		0.70979	0.381443						

* There are two solutions here that contain no rise. The respective amplitudes are put in the Ar1XX columns.

							Emission at 583 nm	
τr1	τr2		τd1	τd2	τd3	τd4		exc. wl. (nm)
52.9558	374.115		409.299	1538.92	3059.22	11839.8	LaF3CeGdEu394_583race.txt	394
194.062			856.159	1723.74	3174.68	12010.6	LaF3CeEu394_583.trace.txt	394
τr1	τr2	τr3	τd1	τd2	τd2	τd3		
36.6724	188.06		1088.04	2612.36		10771.5	LaF3CeEu250_583.trace.txt	250
68.7695	356.244		1758.61		3506.12	12492.9	LaF3CeGdEu272_583.trace.txt	272
189.806	790.436				3888.69	11441.4	LaF3GdEu394_583.trace.txt	394
128.964	814.404	2578.83			4355.05	12312.8	LaF3Eu394_583.trace.txt	394
93.7487	347.712	1569.25			4691.58	12514.7	LaF3GdEu272_583.trace.txt	272
48.8123	205.375	792.347	2088.52		4027.93	12324.8	LaF3CeGdEu250_583.trace.txt	250

							Emission at 552 nm	
τr1	τr2	τr3	τd1	τd2	τd3	τd3		exc. wl. (nm)
				4441.31			LaF3Eu394_552.trace.txt	394
123.08	633.822	1668.06		4442.95			LaF3Eu394_552.trace.txt	394
87.4419	334.957	1523.87		4747.25		11880.5	LaF3GdEu272_552.trace.txt	272
122.067	529.884	1266.15		3837.96	6479.4		LaF3GdEu394_552.trace.txt	394
τr1	τr2		τd1	τd2	τd3	τd4		
36.9908	212.692		948.068	1991.62	3316.66	17802.4	LaF3CeEu250_552.trace.txt	250
35.0901	186.447		1050.84	2022.05	3279.16	10305.5	LaF3CeEu394_552.trace.txt	394
94.6395	506.248		68.4438	2658.39	5710.43	12883.3	LaF3CeGdEu250_552.trace.txt	250
35.4394	202.309		1078.91	2540.29	6959.62		LaF3CeEu250_552.trace.txt	250
78.8142	373.564			1832.85	3531.48	9979	LaF3CeGdEu272_552.trace.txt	272
46.1687	232.01			1510.71	3146.29		LaF3CeGdEu394_552race.txt	394
				2005.58	3512.65		LaF3CeGdEu394_552race.txt	394
